# Catalytic vinylogous cross-coupling reactions of rhenium vinylcarbenoids†
†Electronic supplementary information (ESI) available: Experimental details and characterization of all new compounds, selected 2D-NOESY, 2D-COSY, HMBC, HMQC data. See DOI: 10.1039/c7sc05477g


**DOI:** 10.1039/c7sc05477g

**Published:** 2018-01-30

**Authors:** Ji Chen, Jimmy Wu

**Affiliations:** a Department of Chemistry , Dartmouth College , Hanover , New Hampshire 03755 , USA . Email: jimmy.wu@dartmouth.edu

## Abstract

We report the first example of the rhenium-catalyzed allylation reaction of indolyl compounds by means of cross-coupling with propargyl ethers as non-obvious allylating reagents. Data from isotope-labeling and kinetic isotopic studies are consistent with a mechanism that proceeds by vinylidene formation as the rate determining step, followed by 1,5-hydride shift to generate a key rhenium vinylcarbenoid complex. Bond formation occurs at the vinylogous site and the reaction is conveniently carried out in air.

## Abstract 2

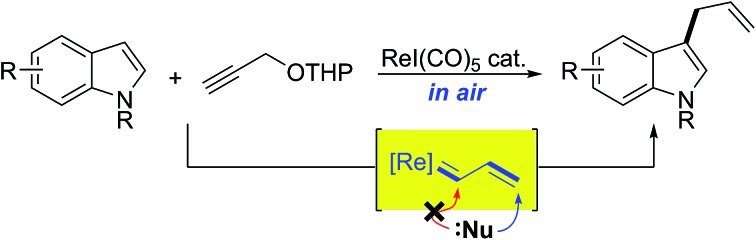
We report the first example of the rhenium-catalyzed allylation reaction of indolyl compounds by means of cross-coupling with propargyl ethers as non-obvious allylating reagents.

## Introduction

**Fig. 1:**
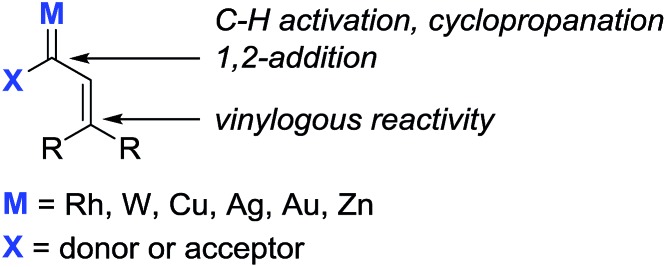
Transition metal vinylcarbenoids.

Transition metal vinylcarbenoids are multifaceted species that can engage in either C–H activation^1^,^2^ or cyclopropanation^3^,^4^ processes (Fig. 1). In the presence of nucleophiles, addition usually occurs at the carbenoid center; however, in recent years, several groups have reported on the vinylogous electrophilicity of these complexes.^5^–^11^ Although the most common metal vinylcarbenoids are those derived from rhodium, tungsten, and copper, complexes involving silver, gold, and zinc are also known. These species are often stabilized by a flanking acceptor (*i.e.* carbonyl) or donor group (*i.e.* alkoxy) and are typically prepared by diazoacetate decomposition,^1^,^3^,^12^ metal insertion into cyclopropenes,^13^,^14^ rearrangement of propargyl carbonates,^15^ or metathesis reactions.^16^,^17^

**Scheme 1:**
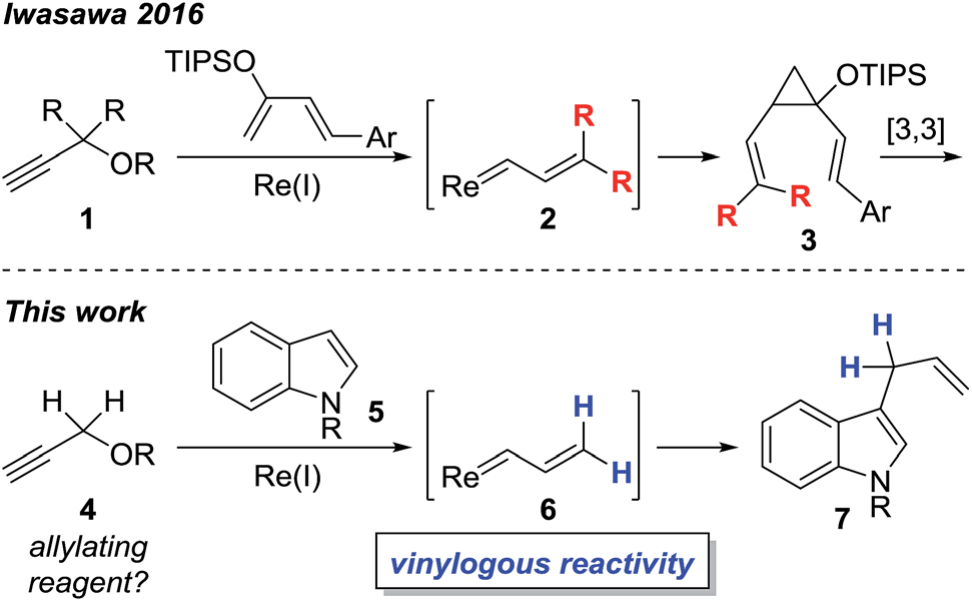
Reactivity of rhenium vinylcarbenoids.

In comparison, the synthesis, physical properties, and reactivity, of rhenium vinylcarbenoids are considerably less well-studied.^18^–^23^ Iwasawa recently reported a novel method for generating rhenium vinylcarbenoids **2** starting from propargyl ethers.^24^ This process is thought to proceed through vinylidene formation and 1,5-hydride transfer. In the presence of siloxybutadienes, cyclopropanation occurs at the carbenoid center to give **3** followed by [3,3]-sigmatropic rearrangement to produce cycloheptadiene derivatives (Scheme 1). Inspired by their work, we wondered whether it would be possible to selectively access the electrophilic nature of rhenium vinylcarbenoids, thereby allowing propargyl ethers to be used as non-obvious synthons for allylating reagents (Scheme 1). The paucity of precedents for this type of strategy likely reflects the challenge that propargyl ethers are in the wrong oxidation state for allylation and would require some form of reductive step.

Herein, we report the first example of a rhenium-catalyzed, vinylogous, allylation of indoles with propargyl ethers. The transformation is complementary to Pd-, Ir-, Rh-based protocols that make use of other types of allyl precursors.^25^–^28^ Moreover, instances of completely unsubstituted vinylcarbene complexes, as exemplified by **6**, are quite unusual as only a handful of examples, even of other metals, have been previously reported.^16^,^17^,^24^,^29^–^34^

We began by examining the reaction between *N*-methylindole **5a** and propargyl tetrahydropyranyl ether **4a** in the presence of various rhenium catalysts. A few representative reactions from the optimization study are summarized in Table 1. The use of Re_2_(CO)_10_ gave no reaction (entry 1), but we were pleased that a range of Re(i) species, including the commercially-available compound ReBr(CO)_5_ efficiently catalyzed the formation of the desired product **7a** in good yield (entry 2). After some experimentation, we identified the use of ReI(CO)_5_ in 1,4-dioxane at 100 °C to be optimal. The reaction requires only a 1 : 1 stoichiometry of indole *versus* propargyl ether, and is carried out in air with no precautions necessary for the removal of oxygen or water.

**Table 1:** Optimization studies^*a*^

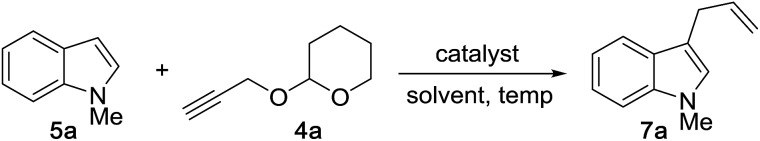				
Entry	Catalyst	Solvent	*T* [°C]	Yield^*b*^ (%)
1	Re_2_(CO)_10_	1,4-Dioxane	100	0
2	ReBr(CO)_5_	1,4-Dioxane	100	81
3	ReI(CO)_5_	1,4-Dioxane	100	91
4	[ReBrCO_3_thf]_2_	1,4-Dioxane	100	65
5	ReI(CO)_5_	THF	60	0
6	ReI(CO)_5_	Toluene	80	0
7	ReI(CO)_5_	DMF	100	0
8	ReI(CO)_5_	1,4-Dioxane	60	0
9	ReI(CO)_5_	1,4-Dioxane	80	Trace
10	ReI(CO)_5_	1,4-Dioxane	100	90^*c*^

^*a*^Reaction conditions: 1 equiv. of **4a**, 1 equiv. of **5a**, and 5 mol% of catalyst (0.2 M) for 10 h in air.

^*b*^Isolated yield.

^*c*^With activated 4 Å MS.

We then explored the reaction scope with respect to the indole substrates which exhibited good compatibility with a range of indoles bearing substituents such as halogen, amide, ester, nitro, boronate ester, and alkoxy (Table 2). The efficiency of the reaction is diminished somewhat when electron-deficient indoles are used (products **7e–g**). Both N–Me and N–Bn derivatives were amenable; however, indoles bearing substituents at the C3 position were found to be inert, indicating a likely steric bias. The compatibility of this reaction with a range of functional groups makes it competitive as a new type of catalytic cross-coupling protocol.

**Table 2:** Scope of indoles^*a*^

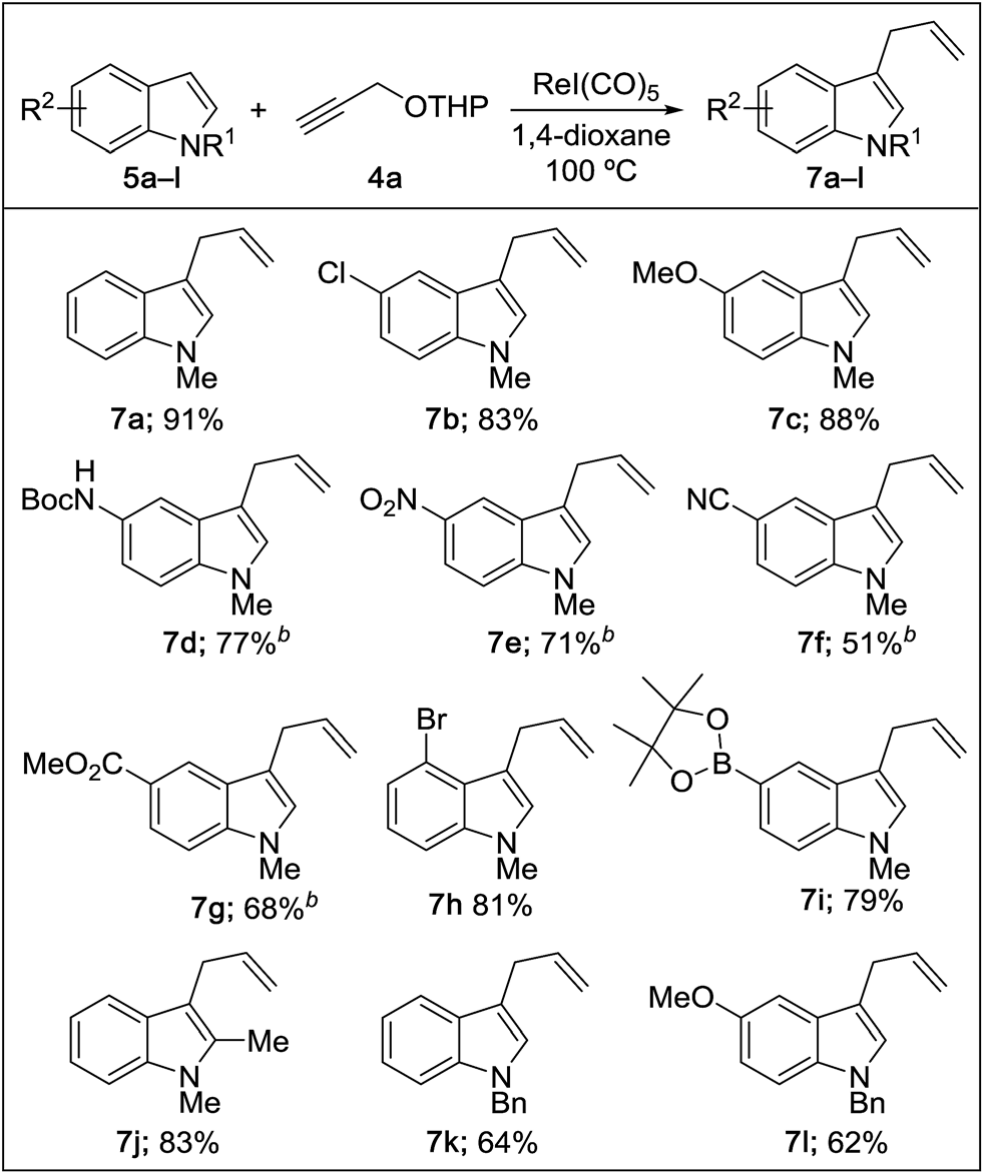

^*a*^Reaction conditions: 1 equiv. of **4a**, 1 equiv. of **5**, and 5 mol% of catalyst (0.2 M) for 10 h in air; isolated yields.

^*b*^1.5 equiv. of **4a** used.

Next, we examined the reaction scope with respect to the propargyl ether (Table 3). The acetal type precursors **4a** and **4b** did not exhibit much difference in either reactivity or reaction rate. However, the benzyl ether precursor **4c** proved to be less efficient and required 1.5 equiv. of **4c**. We believe that the reason for this difference is that the use of **4c** forms benzaldehyde as a side-product, which can react with indole in unproductive pathways. This may be aided by the rhenium catalyst which is able to function as a hard Lewis acid.^35^,^36^ Intriguingly, we found that substituents at the propargylic position have profound effects on the title reaction. The use of monosubstituted acetal **4d** furnished no desired product, whereas the geminally disubstituted acetal **4e** gave an unexpected product assigned as the bis-prenylated compound **8**. That excess amounts of **4a–c** did not lead to the corresponding bis-allylated products suggests a fundamental difference in the reactivity profile of **4e**. Our group is presently studying this reaction in greater detail.

**Table 3:** Scope of propargyl ether^*a*^

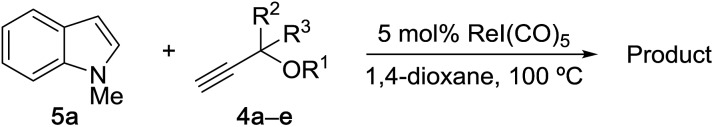		
Propargyl ether	Product	Yield^*b*^/%
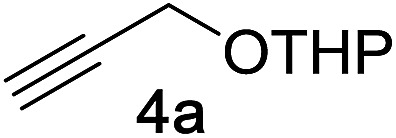	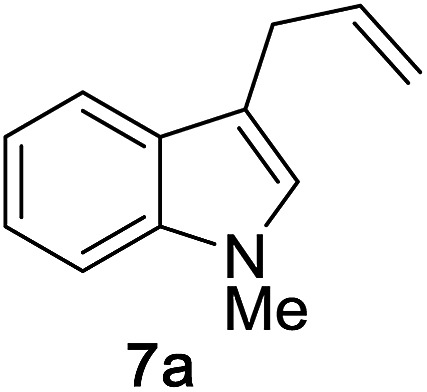	91%
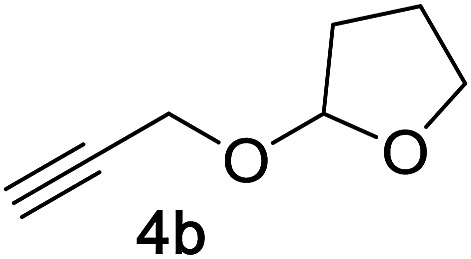		86%
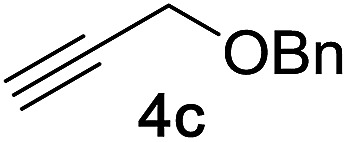		81%^*c*^
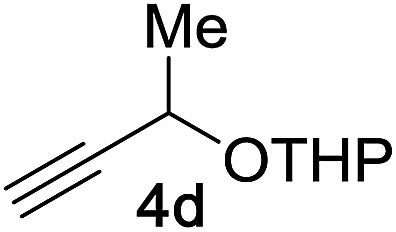	**4d** consumed; no rxn of indole	0%
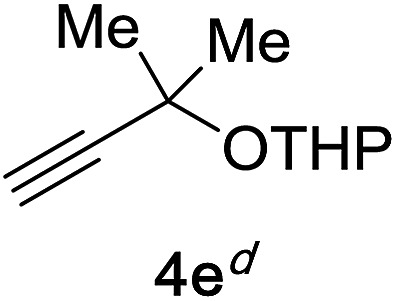	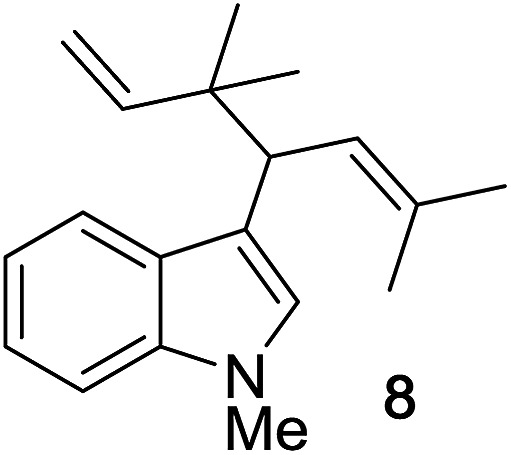	90%

^*a*^Reaction conditions: 1 equiv. of **4a–e**, 1 equiv. of **5a**, and 5 mol% of catalyst (0.2 M) for 10 h in air.

^*b*^Isolated yield.

^*c*^1.5 equiv. of **5a**.

^*d*^2.0 equiv. of **4e**.

**Fig. 2:**
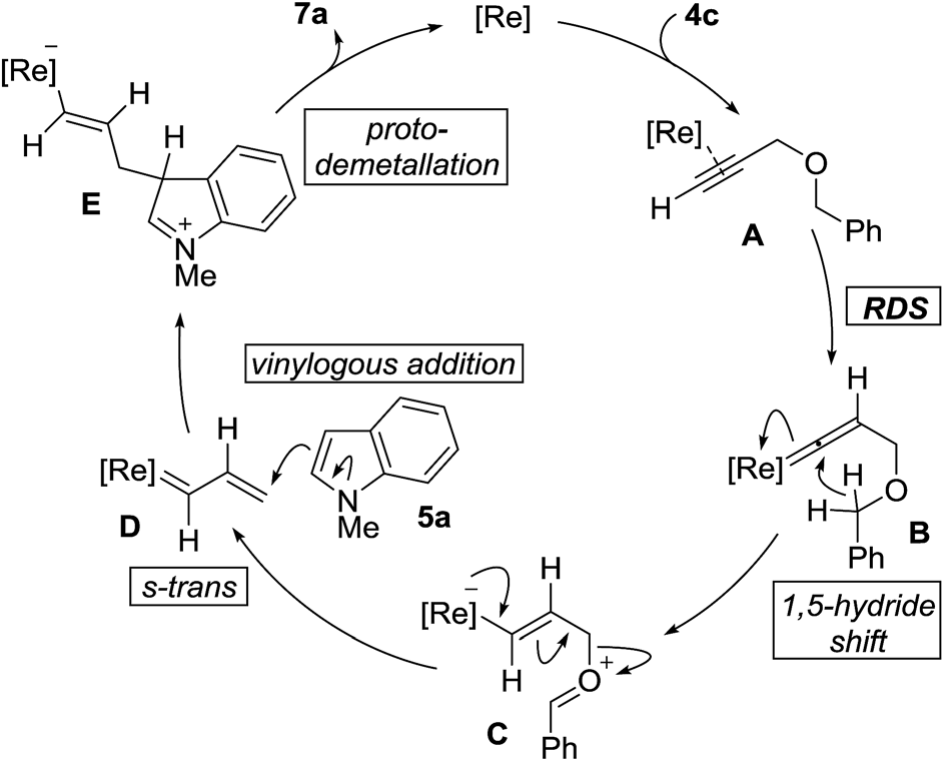
Proposed mechanism.

The proposed mechanism of the cross-coupling, along with salient features, is outlined in Fig. 2. The reaction begins with coordination of the propargyl ether **4c** with the rhenium center followed by vinylidene formation as the rate-determining-step to give **B**. 1,5-Hydride shift generates the zwitterionic species **C**. Elimination of benzaldehyde generates rhenium carbenoid **D**, in the *s-trans* conformation, which can react at the vinylogous position with indole **5a** to give **E**. It should be noted that when using **4a**, δ-valerolactone is produced as the reaction by-product instead of benzaldehyde. Stereospecific protodemetallation followed by rearomatization of **E** would provide the observed product **7a** and regenerate the rhenium catalyst.

**Scheme 2:**
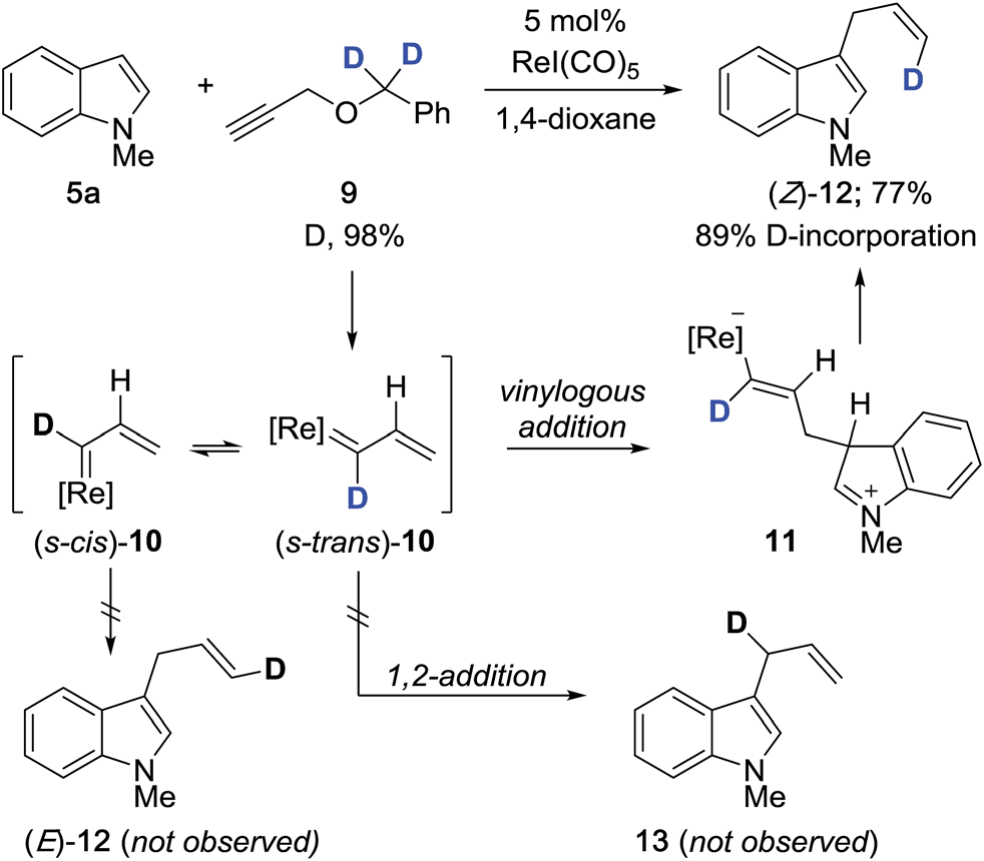
Regioselective and stereoselective cross-coupling with isotopically-labeled propargyl ether **9**.

We carried out a series of isotope-labeling and kinetic isotope studies designed to support our hypotheses and establish a clearer understanding of the reaction mechanism. We began by preparing doubly deuterated propargyl ether **9** which, under the reaction conditions, should generate vinylcarbenoid **10** that is isotopically-labeled at the carbene center (Scheme 2). This intermediate will preferentially adopt the lower energy *s-trans* conformation, to which vinylogous addition by indole leads to formation of terminally-labeled (*Z*)-**12** in 77% yield with 89% deuterium incorporation. The reduced level of deuterium incorporation of the product vis-à-vis **9** (D, 98%) may be attributable in part to a primary kinetic isotope effect (KIE) incurred due to the presence of monodeuterated compound **17** (see Scheme 4). We did not observe the formation of configurational isomer (*E*)-**12** which may form by reaction with (*s-cis*)-**10**, or evidence of regioisomer **13** which may arise by 1,2-addition to **10**.

**Scheme 3:**
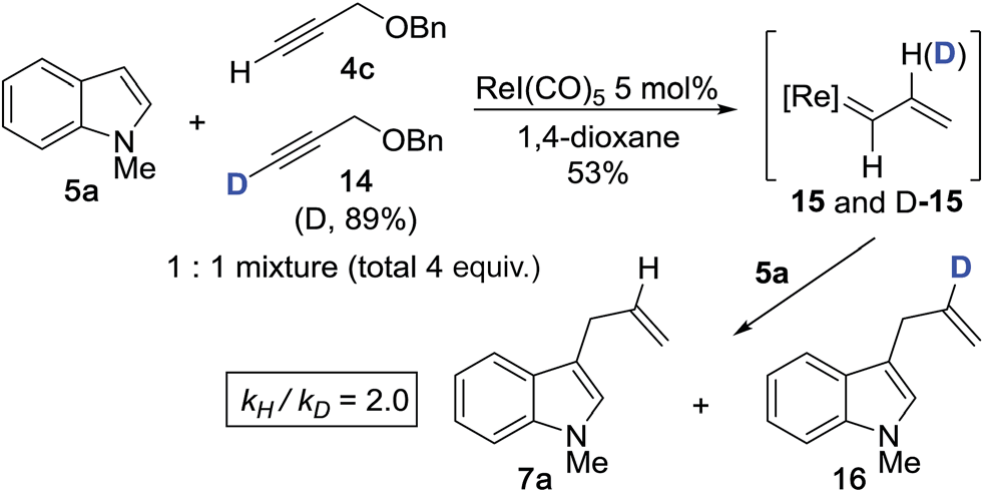
Kinetic isotope effect with isotopically-labeled propargyl ether **14**.

We then performed the cross-coupling reaction between 1 equiv. of **5a** and a 1 : 1 mixture of **4c** and isotopically-labeled propargyl ether **14** (total 4 equiv.) (Scheme 3). Here, we expect formation of both protiated and deuterated vinylcarbenoids, **15** and D-**15**, which will react with indole **5a** to give **7a** and **16**. The reaction was purposefully quenched at partial completion (∼53%), at which point we measured a product ratio of **7a***vs.***16** of 2.0. We interpreted this value as the magnitude of the primary KIE, *k*_H_/*k*_D_ (corrected for % D of **14**), thereby implicating vinylidene formation as the rate determining step (RDS). The extent of this KIE is consistent with an expected non-linear trajectory for the 1,2-hydride shift during vinylidene formation.^37^ The magnitude of the measured KIE is also comparable to values reported for the formation of Ru- and Mo-based vinylidene complexes starting from terminal alkynes.^38^,^39^

**Scheme 4:**
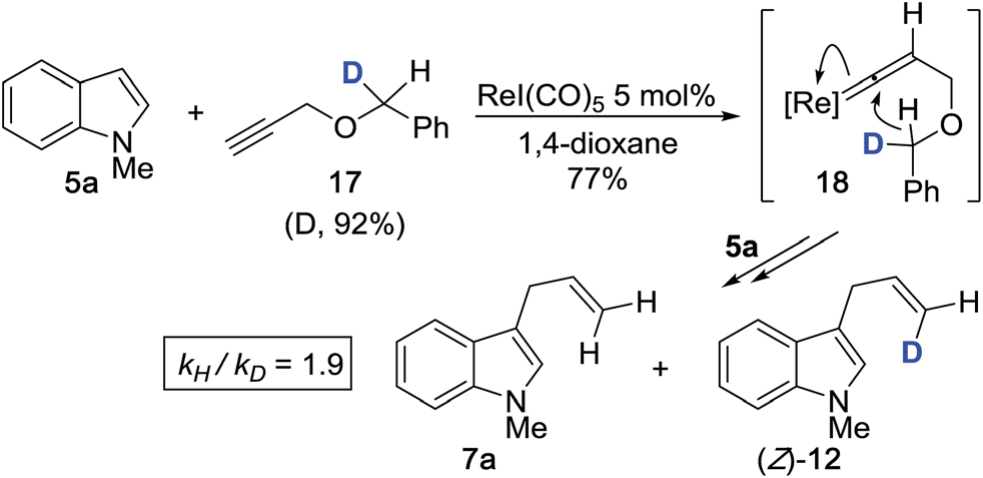
Kinetic isotope effect with monodeuterated propargyl ether **17**.

Next, based on the product ratio of **7a***vs.* (*Z*)-**12** for the reaction between **5a** and monodeuterated propargyl ether **17**, we measured an additional primary KIE of *k*_H_/*k*_D_ = 1.9 (corrected for % D of **17**) (Scheme 4). We expect that treating **17** with ReI(CO)_5_ should induce the formation of **18** which will result in 1,5-transfer of either hydrogen or deuterium. The presence of a discernible primary KIE for this step, which ostensibly occurs after the rate limiting step, suggests that 1,5-hydride transfer is irreversible and product-determining.

## Conclusions

In conclusion, we have reported the first instance of the rhenium catalyzed cross-coupling of indoles with propargyl ethers to give allylated products. The reaction is compatible with a range of functional groups on the indole component. Isotopic labeling studies and KIE measurements establish a mechanistic framework by which vinylidene formation occurs as the rate limiting step followed by product-determining 1,5-hydride shift. Vinylogous attack on the resultant rhenium vinylcarbenoid by indole then furnishes the desired cross-coupled product.

## Conflicts of interest

There are no conflicts to declare.

## Supplementary Material

Supplementary information
