# Supplementary material for: Catalytic vinylogous cross-coupling reactions of rhenium vinylcarbenoids
Source: Chem Sci. 2018 Jan 30;9(9):2489–92. doi: 10.1039/c7sc05477g (PMC5909671; doi:10.1039/c7sc05477g)

Electronic Supplementary Material (ESI) for Chemical Science.
This journal is © The Royal Society of Chemistry 2018

Catalytic Vinylogous Cross-Coupling of Rhenium Vinylcarbenoids

Supporting Information

Ji Chen and Jimmy Wu*

Department of Chemistry, Dartmouth College, Hanover, New Hampshire 03755, United States

General Information 1

Experimental Procedures and Characterization 3

Isotope Labeling and KIE Studies 9

References 10

NMR Spectra 11

General Information

^{1}H-NMR data were recorded on a Bruker Avance III 500 MHz spectrometer (TBI probe) and a Bruker Avance III 600 MHz spectrometer (BBFO probe) with calibration of spectra to $CHCl_3$ (7.26 ppm). ^{13}C-NMR data were recorded at 150 MHz on a Bruker Avance III 600 MHz spectrometer (BBFO probe) at ambient temperature (unless otherwise stated) and are expressed in ppm using solvent as the internal standard ($CDCl_3$ at 77.16 ppm, CD_2Cl_2 at 53.84 ppm). Two-dimensional NMR spectra, including COSY, HSQC, HMBC and NOESY were recorded on a Bruker Avance III 600 MHz spectrometer (BBFO probe). Infrared spectra were recorded on a JASCO FT/IRM4100 Fourier Transform Infrared Spectrometer. Electronic Paramagnetic Resonance spectra was recorded on an Elexsys E500 CW-EPR spectrometer. Chemical shift values (δ) are expressed in ppm downfield relative to internal standard (tetramethylsilane at 0 ppm). Multiplicities are indicated as s (singlet), d (doublet), t (triplet), q (quartet), m (multiplet) and br s (broad singlet). Coupling constants are reported in hertz (Hz). Analytical thin layer chromatography (TLC) was performed on SILICYCLE pre-coated TLC plates (silica gel 60 F-254, 0.25 mm). Visualization was accomplished with UV light and/or with ceric ammonium molybdate (CAM) or $KMnO_4$ staining solutions. Flash column chromatography was performed using Biotage® Isolera System on Biotage® SNAP Ultra columns (part No. FSUL-0442-0010 and FSUL-0442-0025). High resolution mass spectra were acquired from the Mass Spectrometry Laboratory of University of Illinois (Urbana-Champaign, IL).

Reagents were used as received without purification unless stated otherwise. THF, methylene chloride and DMF were dried and purified by solvent system using the Glass Contour Solvent Purification System® (from Pure Process Technology, LLC) by passing the solvents through two drying columns. 1,4-dioxane was purchased from TCI Chemical and dried over activated molecular sieves.

$ReI(CO)_5$ and $[ReBr(CO)_3thf]_2$ were prepared according to literature procedure.[1-2] Starting material compounds indoles **5a–l** and precursor compounds **4a–e** were prepared according to literature procedure.[3-8]

General Procedure for the Re-catalyzed Allylic Alkylation of Indole

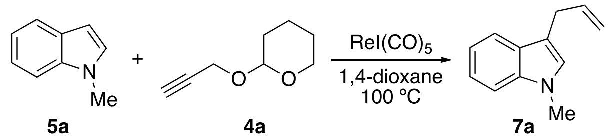

To a screw-capped vial, $ReI(CO)_5$ powder (0.015 mmol, 6.7 mg) and 1.5 mL of 1,4-dioxane were added. The mixture was subjected to sonication for 1 min, followed by the addition of 1-methyl-1-*H*-indole **5a** (0.3 mmol, 37 µL) and 2-(prop-2-yn-1-yloxy)tetrahydro-2*H*-pyran **4a** (0.3 mmol, 42.1 mg). The reaction mixture was stirred in an oil bath at 100 °C until the indole was completely consumed as judged by TLC analysis. The reaction was quenched by diluting with 30 mL of ethyl acetate, then washed with 2 x 10 mL water and 3 x 10 mL brine. The organic phase was then dried over anhydrous Na_2SO_4, and the crude product was obtained by the removal of the volatiles via rotary evaporation. The crude residue was then purified by flash silica chromatography (gradient from 100% hexanes to 95:5 Hexanes/EtOAc) on a Biotage Isolera system to obtain pure product **7a** as a colorless oil in 91% isolated yield.

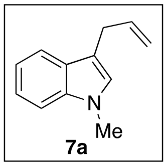

3-allyl-1-methyl-1*H*-indole (**7a**): colorless oil in 91% yield. R_f 0.21 in 2% EtOAc/Hexanes.
^{1}H NMR (500 MHz, $CDCl_3$) δ, ppm 7.60 (1H, td, *J* = 0.6, 8.0 Hz), 7.30 (1H, d, *J* = 7.2 Hz), 7.23 (1H, td, *J* = 7.1, 1.1 Hz), 7.11 (1H, td, J = 7.5, 0.9 Hz), 6.86 (1H, s), 6.12–6.04 (1H, m), 5.17 (1H, ddd, *J* = 1.8, 3.5, 17.1 Hz), 5.07 (1H, ddd, J = 1.9, 3.4, 11.8 Hz), 3.76 (3H, s), 3.53 (2H, ddd, 1.3, 2.4, 6.5 Hz);
^{13}C NMR (150 MHz, $CDCl_3$): 137.5, 137.2, 127.8, 126.5, 121.5, 119.2, 118.7, 115.0, 112.9, 109.1, 32.6, 29.8.
This is a known compound and the NMR spectra are consistent with literature data.[9]

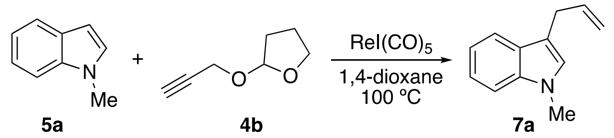

By applying the general allylic alkylation procedure to 1-methyl-1-*H*-indole **5a** (0.3 mmol, 37 µL) and 2-(prop-2-yn-1-yloxy)tetrahydro-2*H*-furan **4b** (0.3 mmol, 37.9 mg), **7a** was isolated in 86% yield.

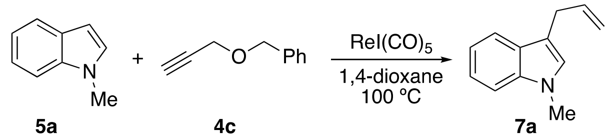

By applying the general allylic alkylation procedure to 1-methyl-1-*H*-indole **5a** (0.45 mmol, 56 µL) and ((prop-2-yn-1-yloxy)methyl)benzene **4c** (0.3 mmol, 43.8 mg), **7a** was isolated in 81% yield.

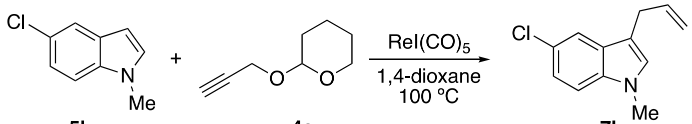

5b **4a** **7b**

By applying the general allylic alkylation procedure to 5-chloro-1-methyl-1*H*-indole **5b** (0.3 mmol, 49.5 mg) and 2-(prop-2-yn-1-yloxy)tetrahydro-2*H*-pyran **4a** (0.3 mmol, 42.2 mg), product **7b** was isolated in 83% isolated yield.

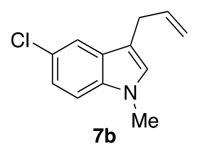

7b

3-allyl-5-chloro-1-methyl-1*H*-indole (**7b**): pale yellow oil. R_f 0.31 in 20% EtOAc/Hexanes.
^{1}H NMR (500 MHz, $CDCl_3$) δ, ppm 7.55 (1H, d, *J* = 1.8 Hz), 7.20–7.15 (2H, m), 6.86 (1H, s), 6.07–6.00 (1H, m), 5.16 (1H, ddd, *J* = 1.8, 3.6, 17.0 Hz), 5.08 (1H, ddd, *J* = 1.3, 2.9, 10.1 Hz), 3.73 (3H, s), 3.47 (2H, dd, *J* = 0.8, 6.4 Hz);
^{13}C NMR (150 MHz, $CDCl_3$): 137.1, 135.6, 128.8, 127.9, 124.6, 121.8, 118.7, 115.4, 112.6, 110.2, 32.8, 29.6.

IR (film, cm^{-1}): 2915, 1478, 1422, 1283, 1143, 913, 791.

HRMS (ESI) calcd. for $C_{12}H_{13}NCl$ (m/z $M+H^+$): 206.0737, found: 206.0736.

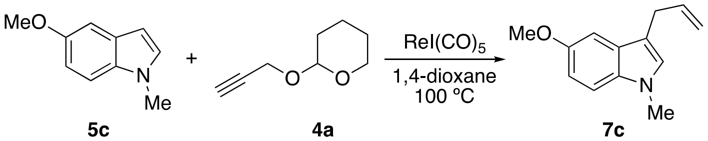

5c **4a** **7c**

By applying the general allylic alkylation procedure to 5-methoxy-1-methyl-1*H*-indole **5c** (0.3 mmol, 48.3 mg) and 2-(prop-2-yn-1-yloxy)tetrahydro-2*H*-pyran **4a** (0.3 mmol, 42.2 mg), product **7c** was isolated in 88% isolated yield.

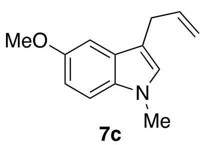

7c

3-allyl-5-methoxy-1-methyl-1*H*-indole (**7c**): colorless oil. R_f 0.28 in 10% EtOAc/Hexanes.
^{1}H NMR (500 MHz, $CDCl_3$) δ, ppm 7.19 (1H, d, *J* = 8.7 Hz), 7.04 (1H, d, *J* = 2.5 Hz), 6.90 (1H, d, *J* = 2.5, 8.7 Hz), 6.83 (1H, s), 6.10–6.04 (1H, m), 5.17 (1H, ddd, *J* = 1.9, 3.6, 17.0 Hz), 5.08 (1H, dd, *J* = 1.7, 10.0 Hz), 3.87 (3H, s), 3.72 (3H, s), 3.49 (2H, dd, *J* = 1.0, 6.7 Hz);
^{13}C NMR (150 MHz, $CDCl_3$): 153.6, 137.4, 132.6, 128.0, 127.2, 115.0, 112.3, 111.7, 109.9, 101.1, 56.0, 32.8, 29.8.

IR (film, cm^{-1}): 2943, 2830, 2040, 1578, 1491, 1226, 1037, 911, 792.

HRMS (ESI) calcd. for $C_{13}H_{16}NO$ (m/z $M+H^+$): 202.1232, found: 202.1226.

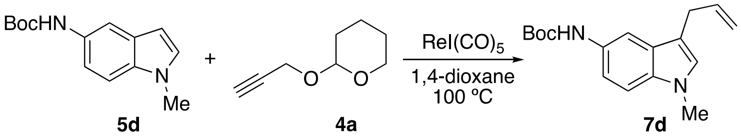

5d **4a** **7d**

By applying the general allylic alkylation procedure to *tert*-butyl (1-methyl-1*H*-indol-5-yl)carbamate **5d** (0.3 mmol, 73.8 mg) and 2-(prop-2-yn-1-yloxy)tetrahydro-2*H*-pyran **4a** (0.45 mmol, 63.3 mg), product **7d** was isolated in 77% isolated yield.

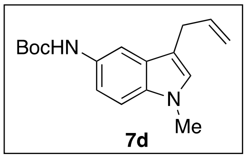

tert-butyl (3-allyl-1-methyl-1*H*-indol-5-yl)carbamate (**7d**): colorless oil. R_f 0.36 in 30% EtOAc/Hexanes.

^{1}H NMR (500 MHz, $CDCl_3$) δ, ppm 7.58 (1H, br s), 7.19–7.16 (2H, m), 6.82 (1H, s), 6.46 (1H, br s), 6.08–6.02 (1H, m), 5.14 (1H, ddd, J = 1.8, 3.5, 17.0 Hz), 5.12 (1H, dd, J = 1.8, 10.0 Hz), 3.48 (2H, d, J = 5.8 Hz), 1.54 (9H, s);
^{13}C NMR (150 MHz, $CDCl_3$): 153.5, 137.4, 134.2, 130.1, 127.9, 127.3, 115.2, 115.0, 112.8, 109.7, 109.3, 79.9, 32.7, 29.6, 28.5.

IR (film, cm^{-1}): 3338, 2976, 2920, 1698, 1540, 1493, 1360, 1240, 1163.

HRMS (ESI) calcd. for $C_{17}H_{23}N_2O_2$ (m/z $M+H^+$): 287.1760, found: 287.1766.

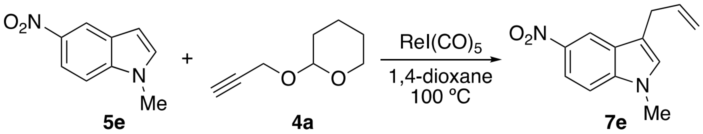

By applying the general allylic alkylation procedure to 5-nitro-1-methyl-1*H*-indole **5e** (0.3 mmol, 52.8 mg) and 2-(prop-2-yn-1-yloxy)tetrahydro-2*H*-pyran **4a** (0.45 mmol, 63.3 mg), product **7e** was isolated in 71% isolated yield.

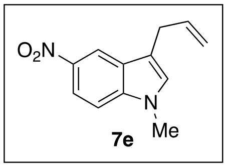

3-allyl-1-methyl-5-nitro-1*H*-indole (**7e**): bright yellow oil. R_f 0.28 in 20% EtOAc/Hexanes.

^{1}H NMR (500 MHz, $CDCl_3$) δ, ppm 8.55 (1H, d, J = 2.2 Hz), 8.12 (1H, dd, J = 3.0, 9.8 Hz), 7.29 (1H, d, J = 9.0 Hz), 6.99 (1H, s), 6.08–5.99 (1H, m), 5.17 (1H, ddd, J = 1.6, 3.3, 17.0 Hz), 5.12 (1H, ddd, J = 1.4, 3.1, 10.2 Hz), 3.81 (3H, s), 3.53 (2H, dd, J = 1.1, 6.4 Hz);
^{13}C NMR (150 MHz, $CDCl_3$): 141.1, 139.9, 136.3, 129.6, 127.1, 117.4, 116.7, 116.1, 116.0, 109.0, 33.1, 29.3.

IR (film, cm^{-1}): 3082, 2910, 2987, 1619, 1513, 1089, 922, 894, 806.

HRMS (ESI) calcd. for $C_{12}H_{13}N_2O_2$ (m/z $M+H^+$): 217.0977, found: 217.0983.

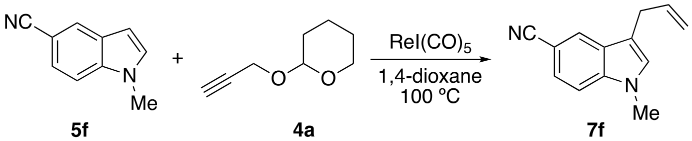

By applying the general allylic alkylation procedure to 1-methyl-1*H*-indole-5-carbonitrile **5f** (0.3 mmol, 47.8 mg) and 2-(prop-2-yn-1-yloxy)tetrahydro-2*H*-pyran **4a** (0.45 mmol, 63.3 mg), product **7f** was isolated in 51% isolated yield.

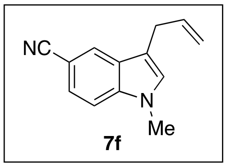

3-allyl-1-methyl-1*H*-indole-5-carbonitrile (**7f**): yellow oil. R_f 0.33 in 30% EtOAc/Hexanes.

^{1}H NMR (500 MHz, $CDCl_3$) δ, ppm 7.93 (1H, d, J = 0.8 Hz), 7.44 (1H, dd, J = 1.4, 8.7 Hz), 7.32 (1H, d, J = 8.3 Hz), 6.96 (1H, s), 6.05–5.97 (1H, m), 5.15 (1H, ddd, J = 1.6, 3.3, 16.8 Hz), 5.10 (1H, ddd, J = 1.2, 3.0, 10.0 Hz), 3.78 (3H, s), 3.54 (2H, dd, J = 1.0, 6.4 Hz);
^{13}C NMR (150 MHz, $CDCl_3$): 138.6, 136.5, 128.7, 125.0, 124.5, 121.0, 115.9, 114.2, 110.0, 101.7, 32.8, 29.4.

IR (film, cm^{-1}): 2921, 2220, 1619, 1491, 1385, 995, 912, 800.

HRMS (ESI) calcd. for $C_{13}H_{13}N_2$ (m/z M+H$^+$): 197.1079, found: 197.1075.

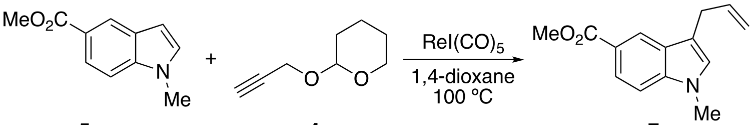

5g **4a** **7g**

By applying the general allylic alkylation procedure to methyl 1-methyl-1*H*-indole-5-carboxylate **5g** (0.3 mmol, 56.8 mg) and 2-(prop-2-yn-1-yloxy)tetrahydro-2*H*-pyran **4a** (0.45 mmol, 63.3 mg), methyl 3-allyl-1-methyl-1*H*-indole-5-carboxylate **7g** was isolated along with an unknown impurity, the yield was adjusted to 68% based on an estimate of approximately 90% purity of **7g**

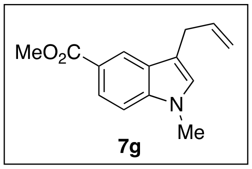

Methyl 3-allyl-1-methyl-1*H*-indole-5-carboxylate (**7g**): colorless oil. R_f 0.29 in 15% EtOAc/Hexanes.
^{1}H NMR (500 MHz, $CDCl_3$) δ, ppm 8.36 (1H, d, *J* = 0.6 Hz), 7.93 (1H, dd, *J* = 1.2, 8.2 Hz), 7.28 (1H, d, *J* = 8.8 Hz), 6.90 (1H, s), 6.11–6.03 (1H, m), 5.17 (1H, dd, *J* = 2.5, 17.6 Hz), 5.09 (1H, dd, *J* = 1.2, 10.0 Hz), 3.94 (3H, s), 3.76 (3H, s), 3.54 (2H, d, *J* = 6.4 Hz);
^{13}C NMR (150 MHz, $CDCl_3$): 168.3, 139.6, 137.0, 128.4, 127.9, 123.0, 122.2, 120.7, 115.5, 114.9, 108.8, 51.8, 32.8, 29.5.

IR (film, cm^{-1}): 2949, 1709, 1615, 1435, 1247, 1104, 769.

HRMS (ESI) calcd. for $C_{14}H_{16}NO_2$ (m/z M+H$^+$): 230.1181, found: 230.1180.

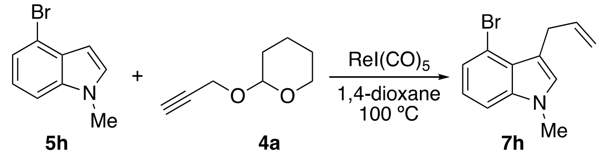

5h **4a** **7h**

By applying the general allylic alkylation procedure to 4-bromo-1-methyl-1*H*-indole **5h** (0.3 mmol, 63.0 mg) and 2-(prop-2-yn-1-yloxy)tetrahydro-2*H*-pyran **4a** (0.3 mmol, 42.2 mg), product **7h** was isolated in 81% isolated yield.

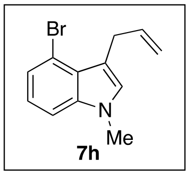

3-allyl-4-bromo-1-methyl-1*H*-indole (**7h**): colorless oil. R_f 0.30 in 10% EtOAc/Hexanes.
^{1}H NMR (500 MHz, $CDCl_3$) δ, ppm 7.26 (1H, dd, *J* = 0.5, 7.4 Hz), 7.22 (1H, dd, *J* = 0.8, 8.3 Hz), 7.02 (1H, t, *J* = 8.1 Hz), 6.87 (1H, s), 6.20–6.12 (1H, m), 5.17 (1H, ddd, *J* = 1.8, 3.7, 8.6 Hz), 5.12 (1H, apparent t, *J* = 1.2 Hz), 3.82 (2H, dd, *J* = 1.2, 6.4 Hz), 3.73 (3H, s);
^{13}C NMR (150 MHz, $CDCl_3$): 138.4, 138.3, 128.3, 125.7, 123.3, 122.3, 115.2, 114.5, 114.2, 108.5, 32.8, 30.6.

IR (film, cm^{-1}): 3071, 2909, 2035, 1540, 1451, 1412, 1257, 1012, 912.

HRMS (ESI) calcd. for $C_{12}H_{13}NBr$ (m/z M+H$^+$): 250.0231, found: 250.0226.

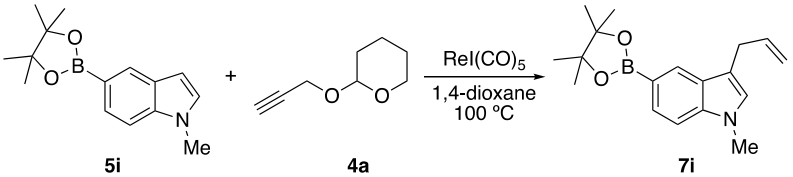

5i 4a 7i

By applying the general allylic alkylation procedure to 1-methyl-5-(4,4,5,5-tetramethyl-1,3,2-dioxaborolan-2-yl)-1*H*-indole **5i** (0.3 mmol, 77.1 mg) and 2-(prop-2-yn-1-yloxy)tetrahydro-2*H*-pyran **4a** (0.3 mmol, 42.2 mg), **7i** was isolated with an unknown impurity, the yield was adjusted to 79% based on the assumption of 90% purity of **7i**.

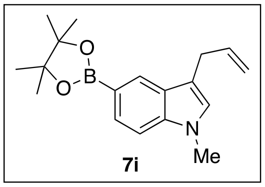

3-allyl-1-methyl-5-(4,4,5,5-tetramethyl-1,3,2-dioxaborolan-2-yl)-1*H*-indole (**7i**): light yellow oil. R_f 0.21 in 10% EtOAc/Hexanes.
^{1}H NMR (500 MHz, $CDCl_3$) δ, ppm 8.11 (1H, s), 7.68 (1H, dd, *J* = 1.2, 8.0 Hz), 7.28 (1H, d, *J* = 8.8 Hz), 6.82 (1H, s), 6.14–6.05 (1H, m), 5.15 (1H, ddd, *J* = 1.7, 3.3, 17.1 Hz), 5.06 (1H, ddd, *J* = 1.4, 3.3, 10.0 Hz), 3.74 (3H, s), 3.54 (2H, dd, *J* = 1.2, 6.6 Hz), 1.38 (12H, s);
^{13}C NMR (150 MHz, $CDCl_3$): 139.1, 137.4, 127.9, 127.5, 127.1, 126.8, 126.6, 115.1, 114.0, 108.5, 83.4, 32.6, 29.5, 24.9.

IR (film, cm^{-1}): 2981, 2931, 2041, 1985, 1613, 1378, 1346, 1139, 962, 689.

HRMS (ESI) calcd. for $C_{18}H_{25}NO_2B$ (m/z $M+H^+$): 298.1978, found: 298.1971.

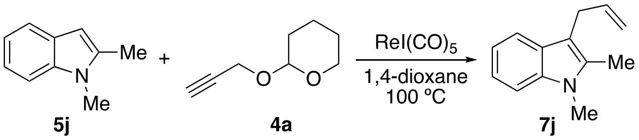

5j 4a 7j

By applying the general allylic alkylation procedure to 1,2-dimethyl-1*H*-indole **5j** (0.3 mmol, 43.5 mg) and 2-(prop-2-yn-1-yloxy)tetrahydro-2*H*-pyran **4a** (0.3 mmol, 42.2 mg), product **7j** was isolated with inseparable starting material 1,2-dimethyl-1*H*-indole. The yield is adjusted to 83% based on H-NMR integrations.

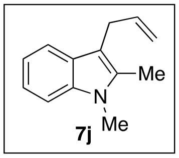

3-allyl-1,2-dimethyl-1*H*-indole (**7j**): colorless oil. R_f 0.30 in 5% EtOAc/Hexanes.
^{1}H NMR (500 MHz, $CDCl_3$) δ, ppm 7.51 (1H, d, *J* = 8.0 Hz), 7.26 (1H, d, *J* = 8.0 Hz), 7.16 (1H, t, *J* = 7.4 Hz), 7.07 (1H, t, *J* = 7.4 Hz), 6.00–5.94 (1H, m), 5.06 (1H, ddd, *J* = 1.6, 3.7, 17.0 Hz), 4.99 (1H, dd, *J* = 1.7, 9.9 Hz), 3.66 (3H, s), 3.49 (2H, d, *J* = 6.8 Hz), 2.36 (3H, s);
^{13}C NMR (150 MHz, $CDCl_3$): 137.7, 136.6, 133.2, 127.7, 120.5, 118.7, 118.1, 114.2, 108.6, 108.5, 29.5, 28.9, 10.2.

IR (film, cm^{-1}): 3059, 2915, 1474, 1368, 1252, 1184, 990, 906, 739.

HRMS (ESI) calcd. for $C_{13}H_{16}N$ (m/z $M+H^+$): 186.1283, found: 186.1287.

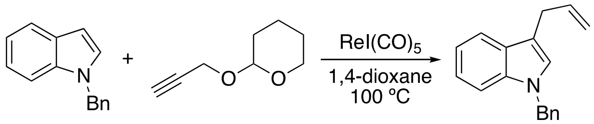

5k **4a** **7k**

By applying the general allylic alkylation procedure to 1-benzyl-1*H*-indole **5k** (0.3 mmol, 62.1 mg) and 2-(prop-2-yn-1-yloxy)tetrahydro-2*H*-pyran **4a** (0.45 mmol, 63.3 mg), product **7k** was isolated with inseparable starting material 1-benzyl-1H-indole **5k**. The yield is adjusted to 64% based on H-NMR integrations.

3-allyl-1-benzyl-1*H*-indole (**7k**): a yellow oil. R_f 0.35 in 5% EtOAc/Hexanes.

This is a known compound and the ^{1}H-NMR and ^{13}C-NMR spectra of the isolated mixture contains compound **7k** according to the literature data.[10]

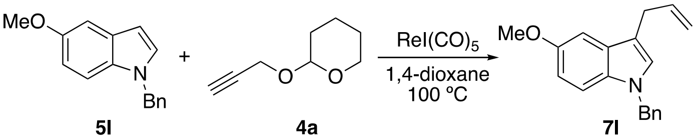

5l **4a** **7l**

By applying the general allylic alkylation procedure to 1-benzyl-5-methoxy-1*H*-indole **5l** (0.3 mmol, 71.1 mg) and 2-(prop-2-yn-1-yloxy)tetrahydro-2*H*-pyran **4a** (0.45 mmol, 63.3 mg), product **7l** was isolated with inseparable unknown impurity in 71% yield. The actual yield of **7l** was estimated at around 62% based on the 90% purity.

3-allyl-1-benzyl-5-methoxy-1*H*-indole (**7l)**: yellow oil. R_f 0.32 in 15% EtOAc/Hexanes.

This is a known compound and the 1H-NMR and 13C-NMR spectra of the isolated mixture contains compound **3l** according to the literature data.[11]

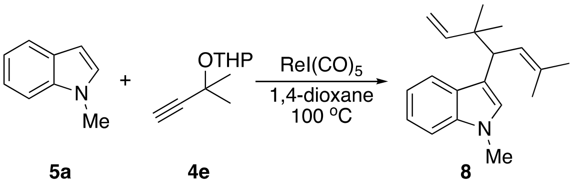

5a **4e** **8**

By applying the general allylic alkylation procedure to 1-methyl-1*H*-indole **5a** (0.3 mmol, 39.3 mg) and 2-((2-methylbut-3-yn-2-yl)oxy)tetrahydro-2*H*-pyran **4e** (0.6 mmol, 100.8 mg), product **8** was isolated with 90% yield.

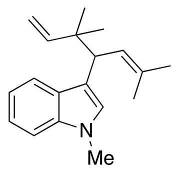

8

1-methyl-3-(3,3,6-trimethylhepta-1,5-dien-4-yl)-1*H*-indole (**8**): yellow oil. R_f 0.36 in 5% EtOAc/Hexanes

^{1}H NMR (600 MHz, $CDCl_3$) δ, ppm 7.67 (1H, d, *J* = 7.5 Hz), 7.27 (1H, d, *J* = 10.0 Hz), 7.20 (1H, t, *J* = 8.4 Hz), 7.10 (1H, t, *J* = 6.7 Hz), 6.85 (1H, s), 6.01 (1H, dd, *J* = 11.1, 17.8 Hz), 5.56 (1H, dd, *J* = 2.2, 10.4 Hz), 4.96 (2H, ddd, *J* = 2.2, 11.0, 18.6 Hz), 3.75 (4H, m), 1.73 (3H, s), 1.64 (3H, s), 1.08 (3H, s), 1.02 (3H, s).

^{13}C NMR (150 MHz, $CDCl_3$) δ, ppm 147.2, 136.3, 130.7, 128.7, 126.8, 126.2, 121.1, 119.9, 118.5, 116.3, 111.1, 109.0, 45.0, 41.2, 32.7, 26.1, 25.6, 25.5, 18.1.

IR (film, cm^{-1}): 3053, 2961, 2924, 2901, 1468, 1373, 1328, 1154, 909, 738.

HRMS (ESI) calcd. for C19H26N2 (m/z $M+H^+$): 268.2065, found: 268.2066.

Isotopic Labeling and KIE Experiments

Deuterated compound **9**, **14** and **17** were prepared according to the literature procedure.[12-14]

1. Isotopic Labeling Experiment

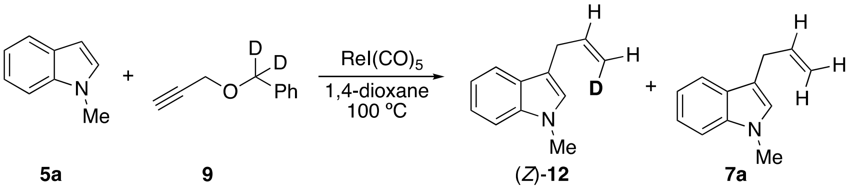

5a **9** (*Z*)-**12** **7a**

By applying the general allylic alkylation procedure to 1-methyl-1*H*-indole (0.3 mmol, 39.3 mg) **5a** and ((prop-2-yn-1-yloxy)methyl-d_2)benzene **9** (D, 98%) (0.3 mmol, 44.5 mg), deuterio product (*Z*)-**12** and protio product **7a** were isolated as an inseparable 9:1 mixture, respectively, with a combined yield of 77%.

2. Determination of Rate Determining Step by Kinetic Isotopic Effect

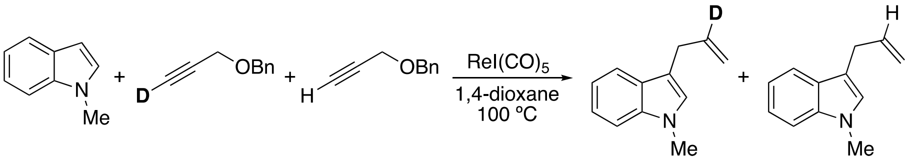

5a **14** **4c** **16** **7a**

To a screw capped vial, ReI(CO)$_5$ powder (0.05 mmol, 22.4 mg) and 8 mL of 1,4-dioxane were added. The mixture was subjected to sonication for 1 min, followed by the addition of 1-methyl-1-*H*-indole **5a** (1 mmol, 131.7 mg), deuterio propargyl ether **14** (D, 89%) (2 mmol, 294.4 mg) and protio propargyl ether **4c** (2 mmol, 292.0 mg). The reaction mixture was stirred in an oil bath which was preheated to 100 ºC. Aliquots of 0.5 mL of the reaction mixture were taken respectively 0.5 h, 1 h, 2 h and 3 h after the reaction started. The residue of the reaction mixture was collected by the removal of organic solvent via rotary evaporation and then subjected to ^{1}H-NMR spectroscopy. The residue of the 3 h aliquot was purified by flash silica chromatography (gradient from 100% hexanes to 95:5 Hexanes/EtOAc) on a Biotage Isolera system to obtain an inseparable mixture of both the deuterio and protio allylic alkylation products **16** and **7a**, along with unreacted starting materials. After the mass of the starting material contaminants was subtracted from the total mass of the purified sample (as judged by 1H NMR), we calculated a 53% combined yield of **16** and **7a** based on the 0.5 mL volume of the 3 h aliquot. The ratio between **16** and **7a** was found to be 1:2.4, respectively. This ratio, which was corrected to 1:2 to account for the starting deuterium level of **14** (D, 89%), was similar to the ratio observed in the unpurified 3 h sample and was interpreted to represent the KIE for this reaction.

3. Determination of the Product Distribution Step by Kinetic Isotopic Effect.

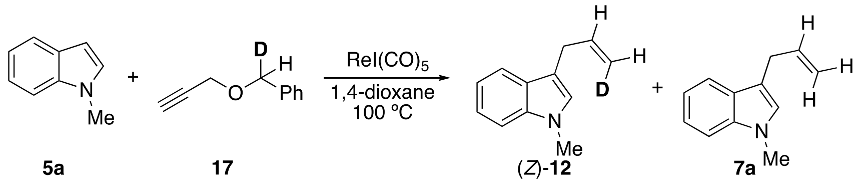

By applying the general allylic alkylation procedure to 1-methyl-1*H*-indole **5a** (0.3 mmol, 39.3 mg) and ((prop-2-yn-1-yloxy)methyl-*d*)benzene **17** (D, 92%) (0.3 mmol, 44.1 mg), deuterio product (*Z*)-**12** and protio product **7a** were isolated as an inseparable 1:2.1 mixture, respectively, with a combined yield of 77%. This ratio was corrected to 1:1.9 to account for the starting deuterium level of **17** (D, 91.5%) and interpreted to represent the KIE for this reaction,

References

1. Darst, K. P.; Lukehart, C. M., *J. Organomet. Chem.*, **1979,** *171*, 65-71.
2. Kuninobu, Y. K., A.; Takai, K., *J. Am. Chem. Soc.,* **2005,** *127*, 13498–13499.
3. Klare, H. F. T.; Oestreich, M.; Ito, J.-i.; Nishiyama, H.; Ohki, Y.; Tatsumi, K., *J. Am. Chem. Soc.,* **2011,** *133*, 3312-3315.
4. Stadlwieser, J. F.; Dambaur, M. E., *Helv. Chim. Acta,* **2006,** *89*, 936-946.
5. Lane, B. S.; Brown, M. A.; Sames, D., *J. Am. Chem. Soc.*, **2005,** *127*, 8050-8057.
6. Wang, X.; Lane, B. S.; Sames, D., *J. Am. Chem. Soc.*, **2005,** *127*, 4996-4997.
7. Farran, D.; Slawin, A. M. Z.; Kirsch, P.; O'Hagan, D., *J. Org. Chem.,* **2009,** *74*, 7168-7171.
8. Velasco, B. E.; Fuentes, A.; Gonzalez, C.; Corona, D.; García-Orozco, I.; Cuevas-Yañez, E., *Synth. Commun.,* **2011,** *41*, 2966-2973.
9. Bissember, A. C.; Levina, A.; Fu, G. C., *J. Am. Chem. Soc.*, **2012,** *134*, 14232-14237.
10. Zhang, Y.; Stephens, D.; Hernandez, G.; Mendoza, R.; Larionov, O. V., *Chem. Eur. J.,* **2012,** *18*, 16612-16615.
11. Xiong, H.; Xu, H.; Liao, S.; Xie, Z.; Tang, Y., *J. Am. Chem. Soc.*, **2013,** *135*, 7851-7854.
12. Kwart, H.; Slutsky, J.; Sarner, S. F., *J. Am. Chem. Soc.*, **1973,** *95,* 5242-5245.
13. Hanzlik, R. P.; Hogberg, K.; Moon, J. B.; Judson, C. M., *J. Am. Chem. Soc.*, **1985,** *107,* 7164-7167.
14. Yamada, T.; Park, K.; Monguchi, Y.; Sawama, Y.; Sajiki, H., *RSC Advances,* **2015,** *5,* 92954-92957.

NMR Spectra

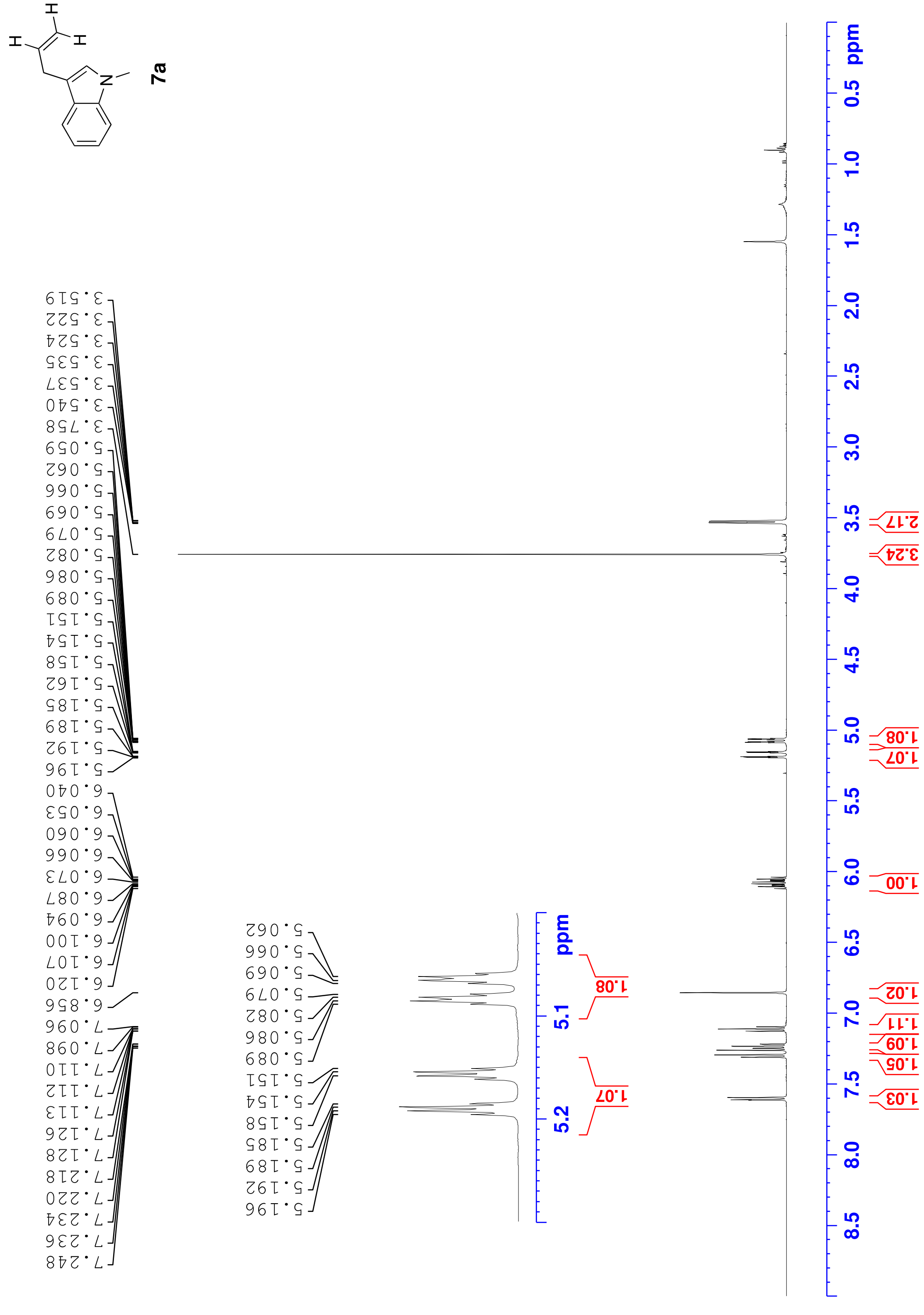
7a
3.519
3.522
3.524
3.535
3.537
3.540
3.758
5.059
5.062
5.066
5.069
5.079
5.082
5.086
5.089
5.151
5.154
5.158
5.162
5.185
5.189
5.192
5.196
6.040
6.053
6.060
6.066
6.073
6.087
6.094
6.100
6.107
6.120
6.856
7.096
7.098
7.110
7.112
7.113
7.126
7.128
7.218
7.220
7.234
7.236
7.248
2.17
3.24
1.08
1.07
1.00
1.02
1.11
1.09
1.05
1.03
ppm

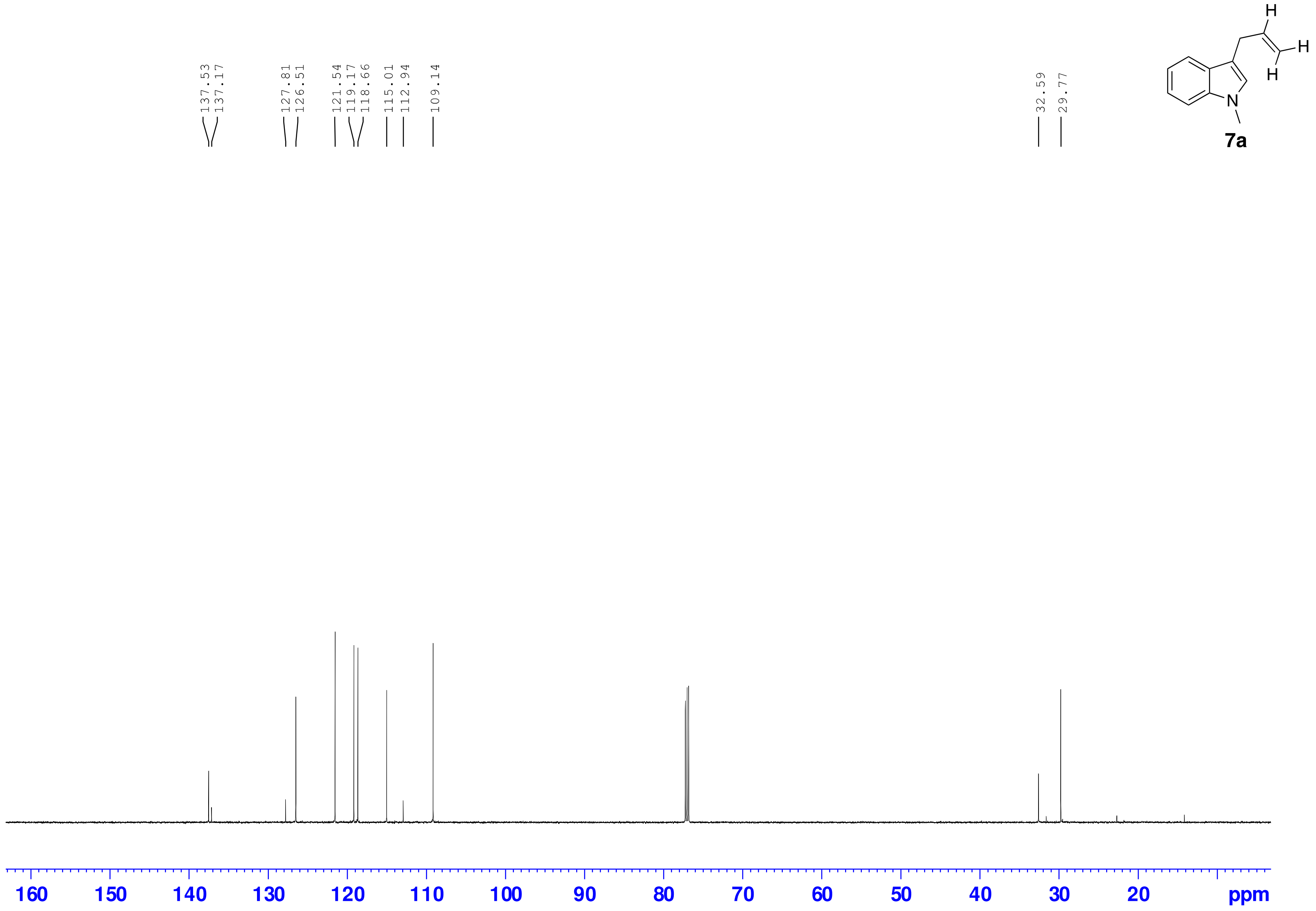
7a
137.53
137.17
127.81
126.51
121.54
119.17
118.66
115.01
112.94
109.14
32.59
29.77
160
150
140
130
120
110
100
90
80
70
60
50
40
30
20
ppm

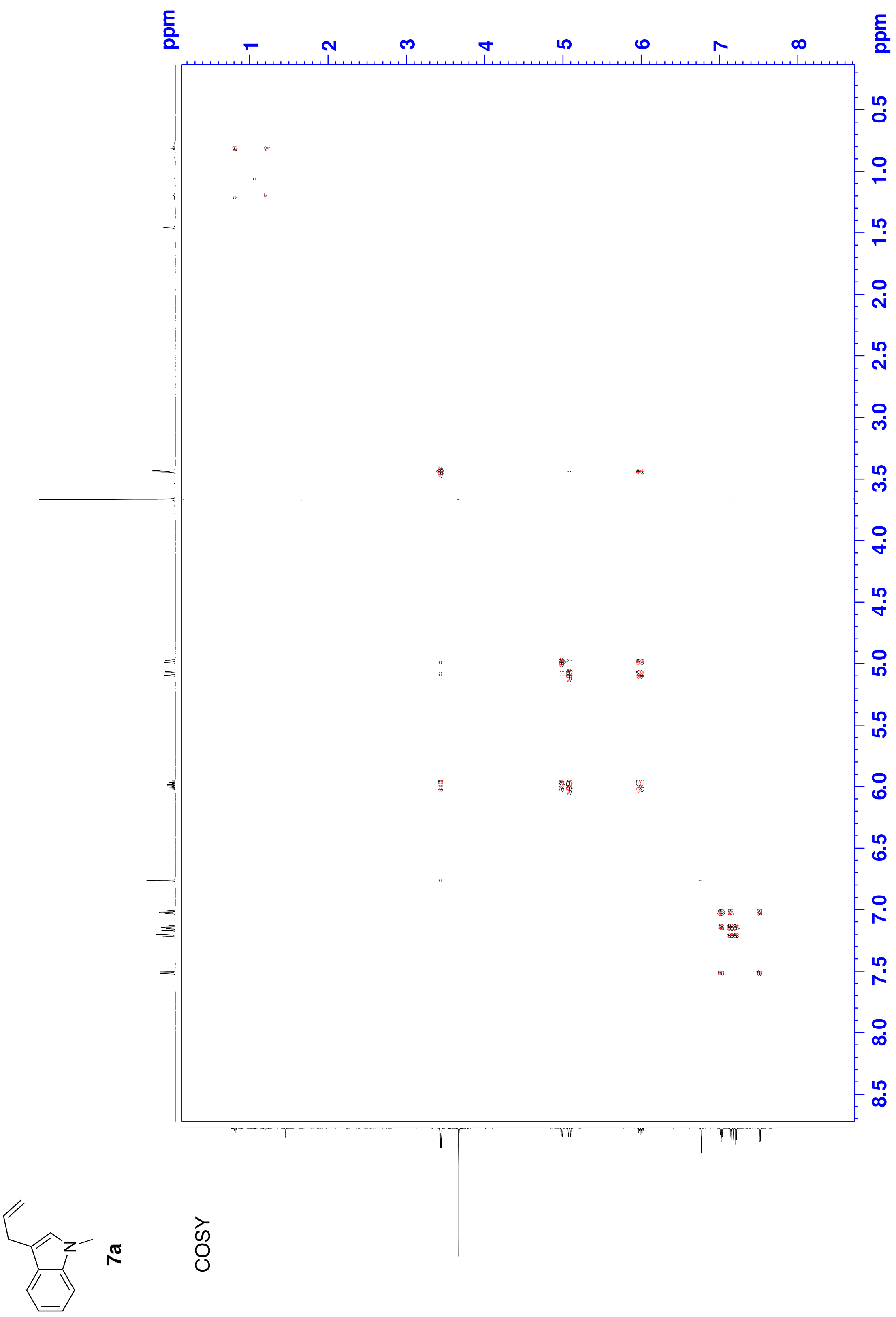
7a
COSY
ppm
0.5
1.0
1.5
2.0
2.5
3.0
3.5
4.0
4.5
5.0
5.5
6.0
6.5
7.0
7.5
8.0
8.5
1
2
3
4
5
6
7
8

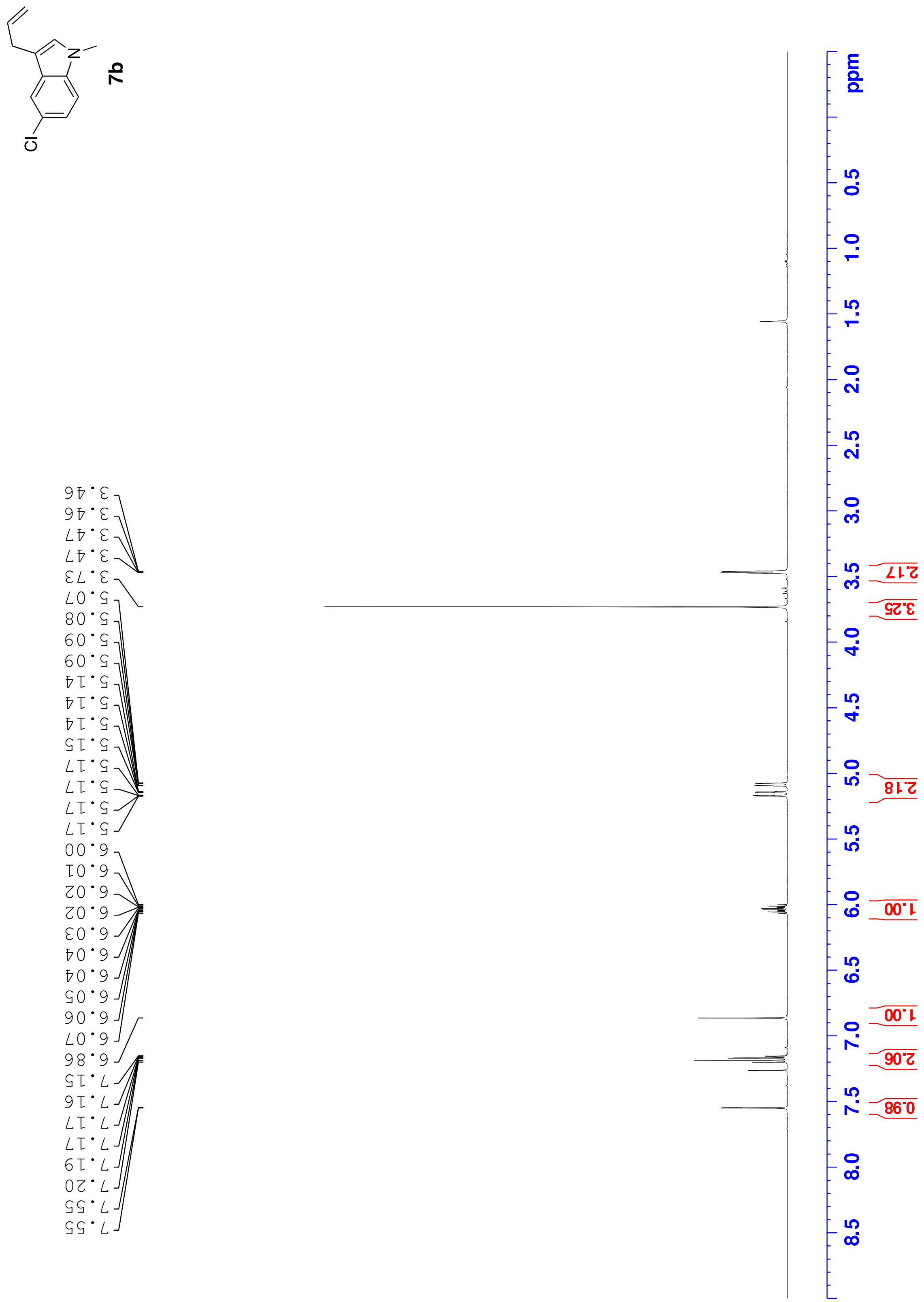
Cl
N
7b
3.46
3.46
3.47
3.47
3.73
5.07
5.08
5.09
5.09
5.14
5.14
5.14
5.15
5.17
5.17
5.17
5.17
6.00
6.01
6.02
6.02
6.03
6.04
6.04
6.05
6.06
6.07
6.86
7.15
7.16
7.17
7.17
7.19
7.20
7.55
7.55
ppm
0.5
1.0
1.5
2.0
2.5
3.0
3.5
4.0
4.5
5.0
5.5
6.0
6.5
7.0
7.5
8.0
8.5
2.17
3.25
2.18
1.00
1.00
2.06
0.98

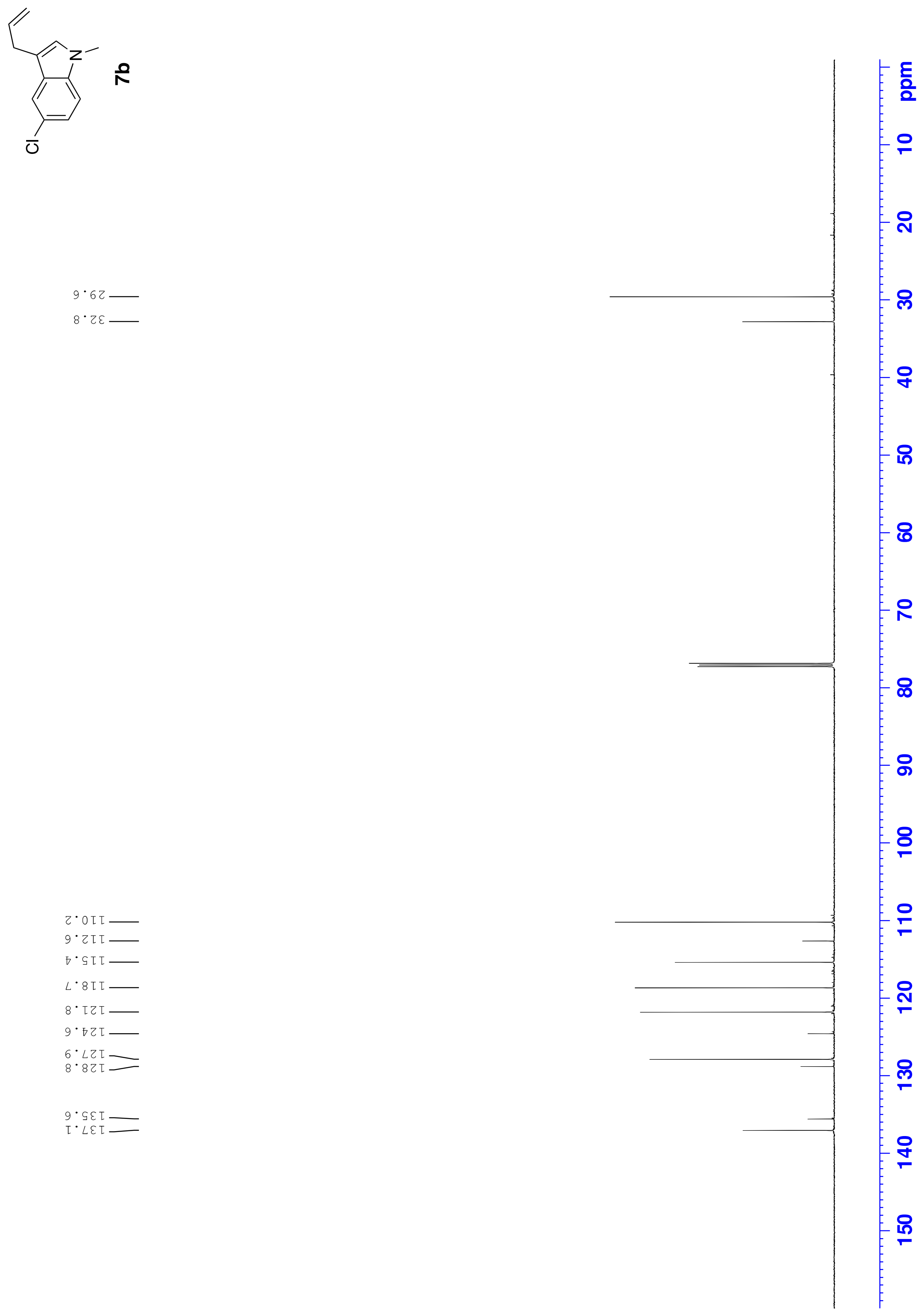
7b
Cl
N
29.6
32.8
110.2
112.6
115.4
118.7
121.8
124.6
127.9
128.8
135.6
137.1
ppm
10
20
30
40
50
60
70
80
90
100
110
120
130
140
150

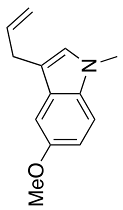
MeO
N

7c

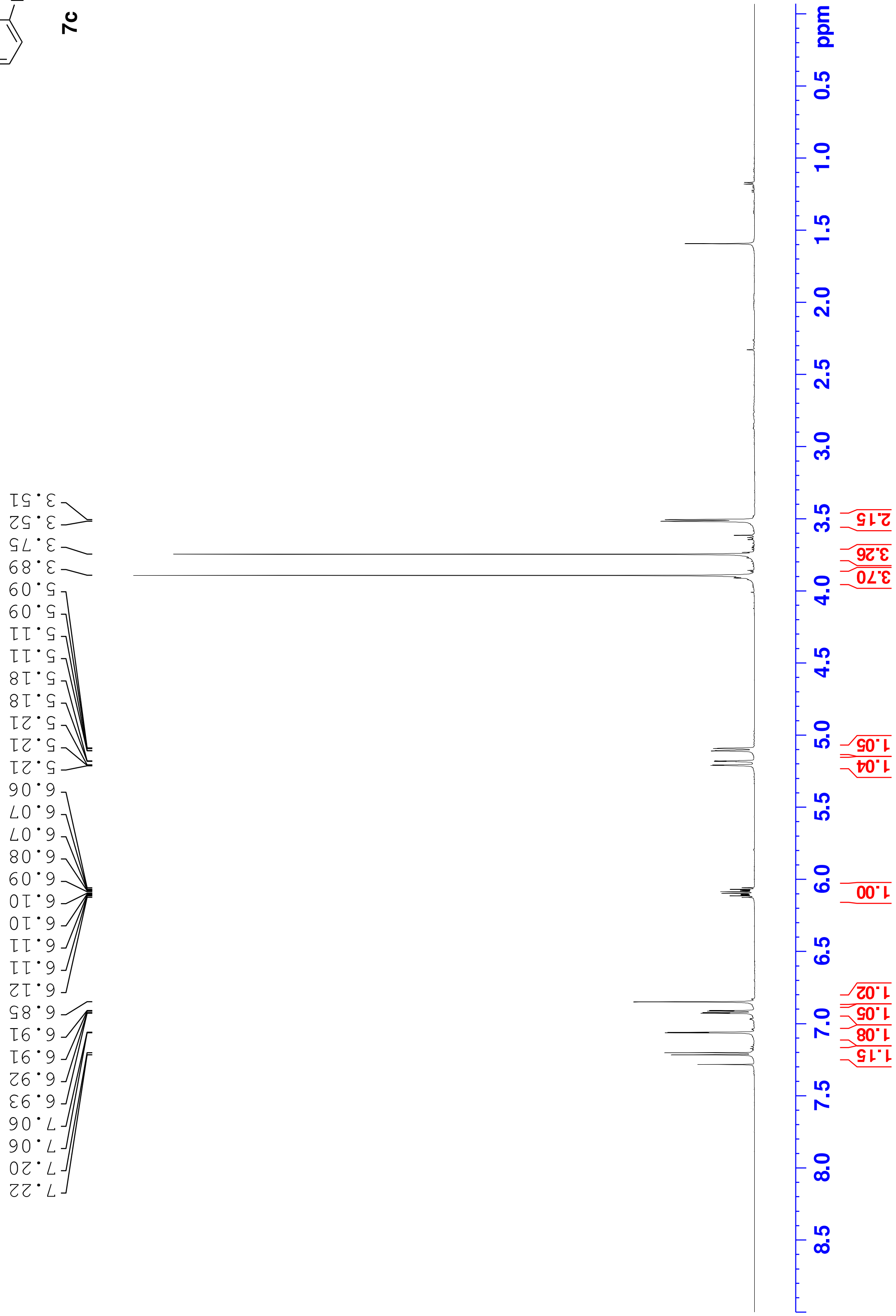
3.51
3.52
3.75
3.89
5.09
5.09
5.11
5.11
5.18
5.18
5.21
5.21
5.21
6.06
6.07
6.07
6.08
6.09
6.10
6.10
6.11
6.11
6.12
6.85
6.91
6.91
6.92
6.93
7.06
7.06
7.20
7.22
2.15
3.26
3.70
1.05
1.04
1.00
1.02
1.05
1.08
1.15
ppm
0.5
1.0
1.5
2.0
2.5
3.0
3.5
4.0
4.5
5.0
5.5
6.0
6.5
7.0
7.5
8.0
8.5

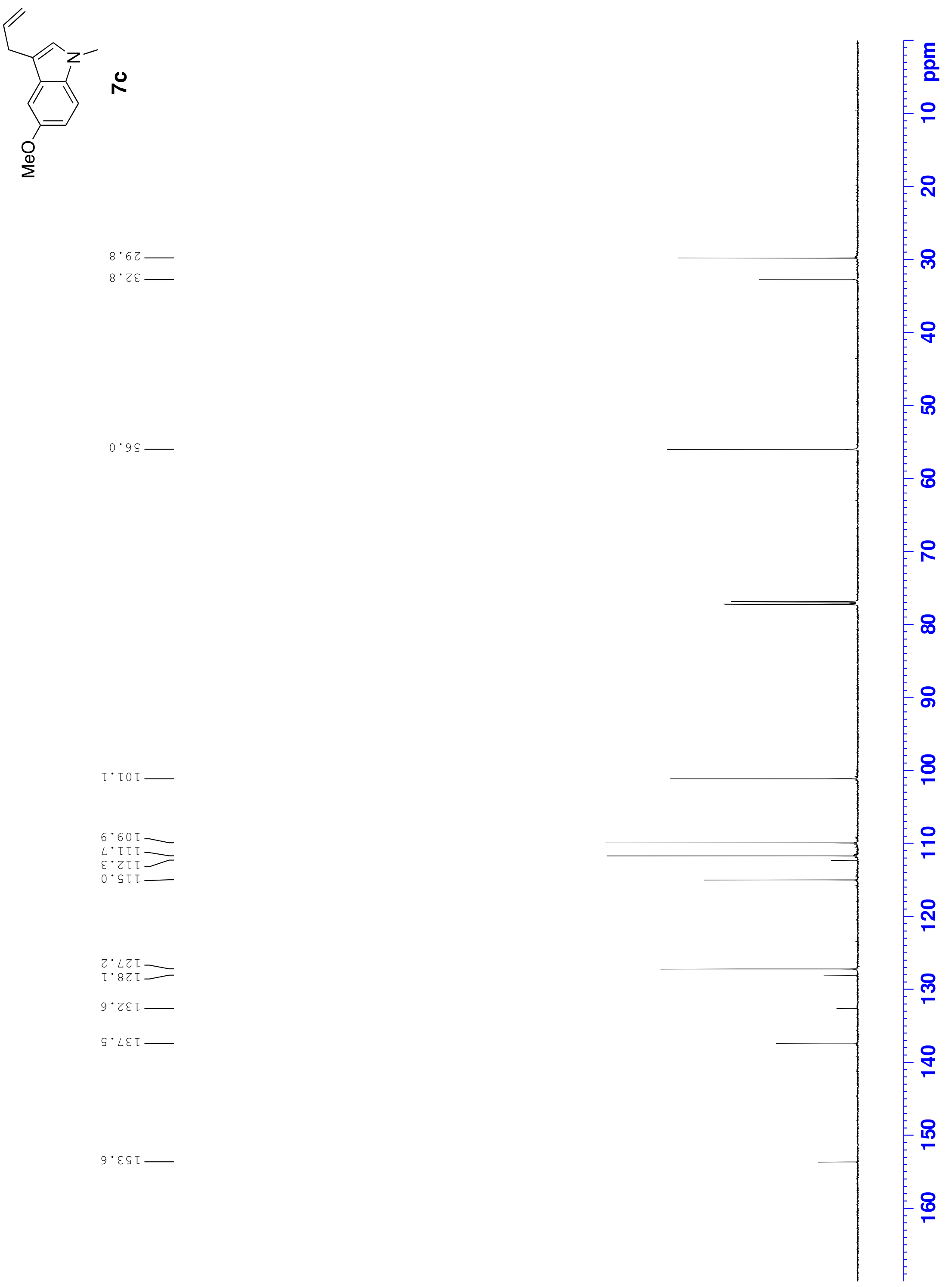

MeO
N
7c
29.8
32.8
56.0
101.1
109.9
111.7
112.3
115.0
127.2
128.1
132.6
137.5
153.6
ppm
10
20
30
40
50
60
70
80
90
100
110
120
130
140
150
160

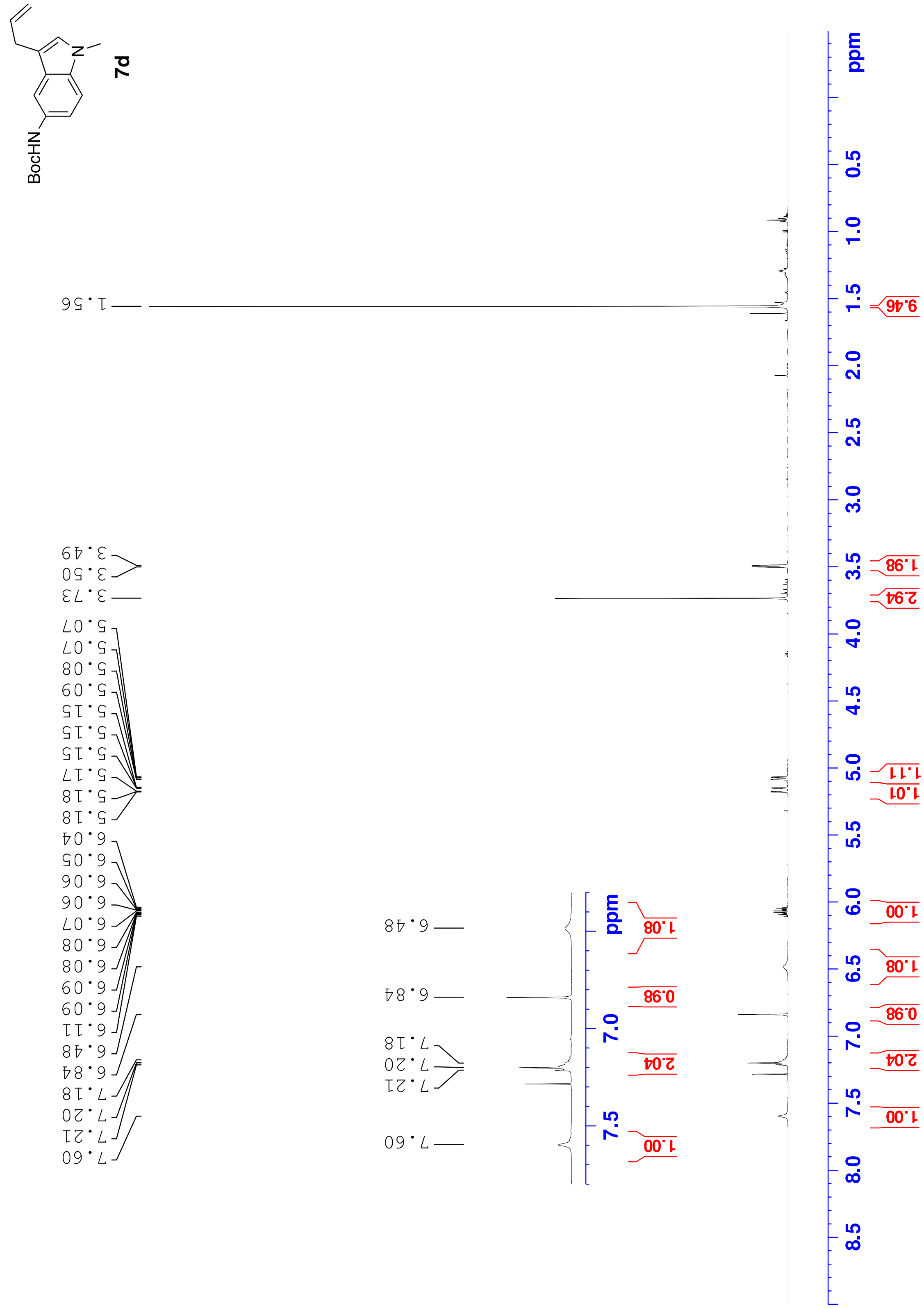
BocHN
N
7d
1.56
3.49
3.50
3.73
5.07
5.07
5.08
5.09
5.15
5.15
5.15
5.17
5.18
5.18
6.04
6.05
6.06
6.06
6.07
6.08
6.08
6.09
6.09
6.11
6.48
6.84
7.18
7.20
7.21
7.60
9.46
1.98
2.94
1.11
1.01
1.00
1.08
0.98
2.04
1.00
ppm

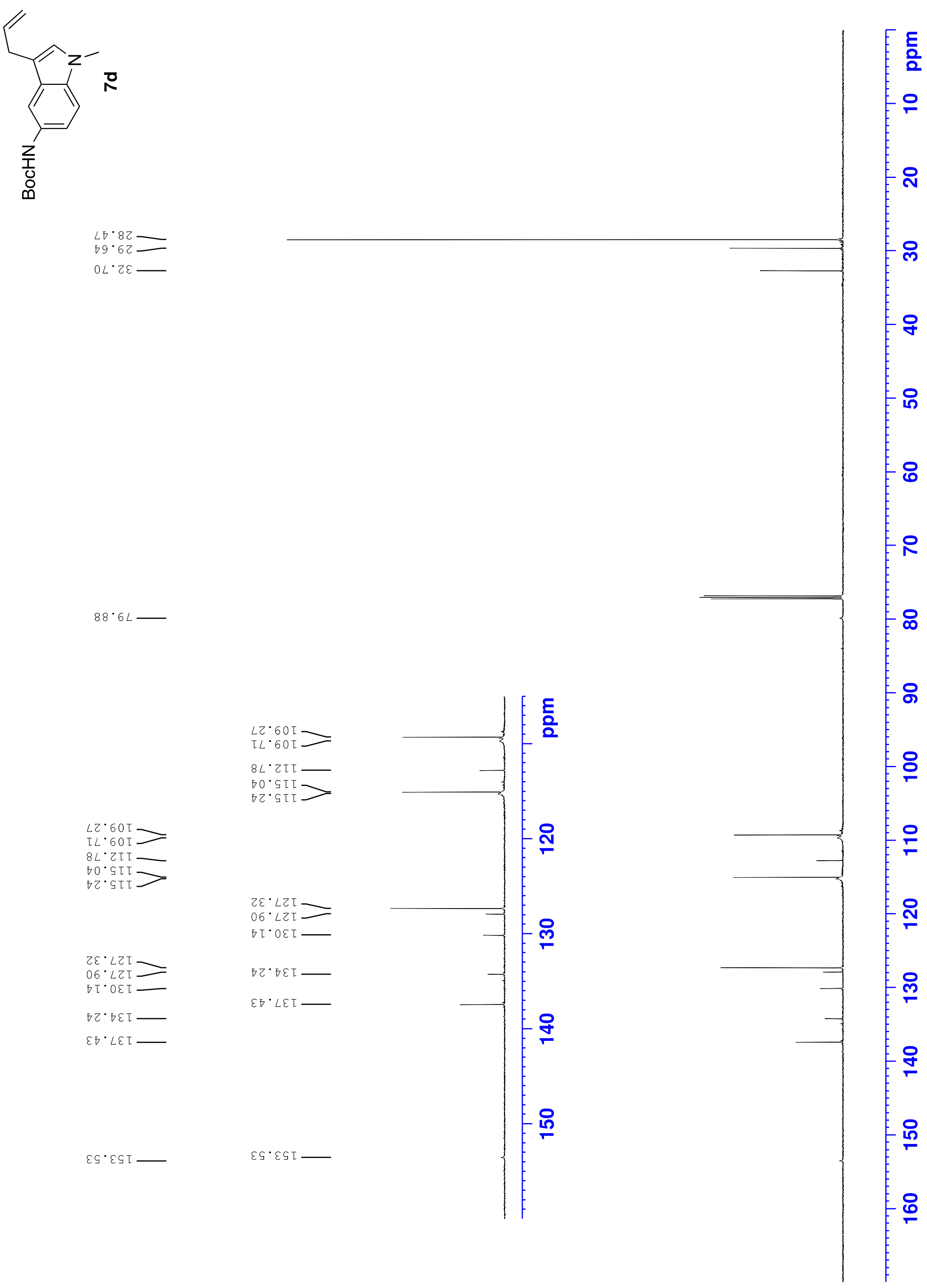
BocHN
N
7d
28.47
29.64
32.70
79.88
109.27
109.71
112.78
115.04
115.24
127.32
127.90
130.14
134.24
137.43
153.53
ppm

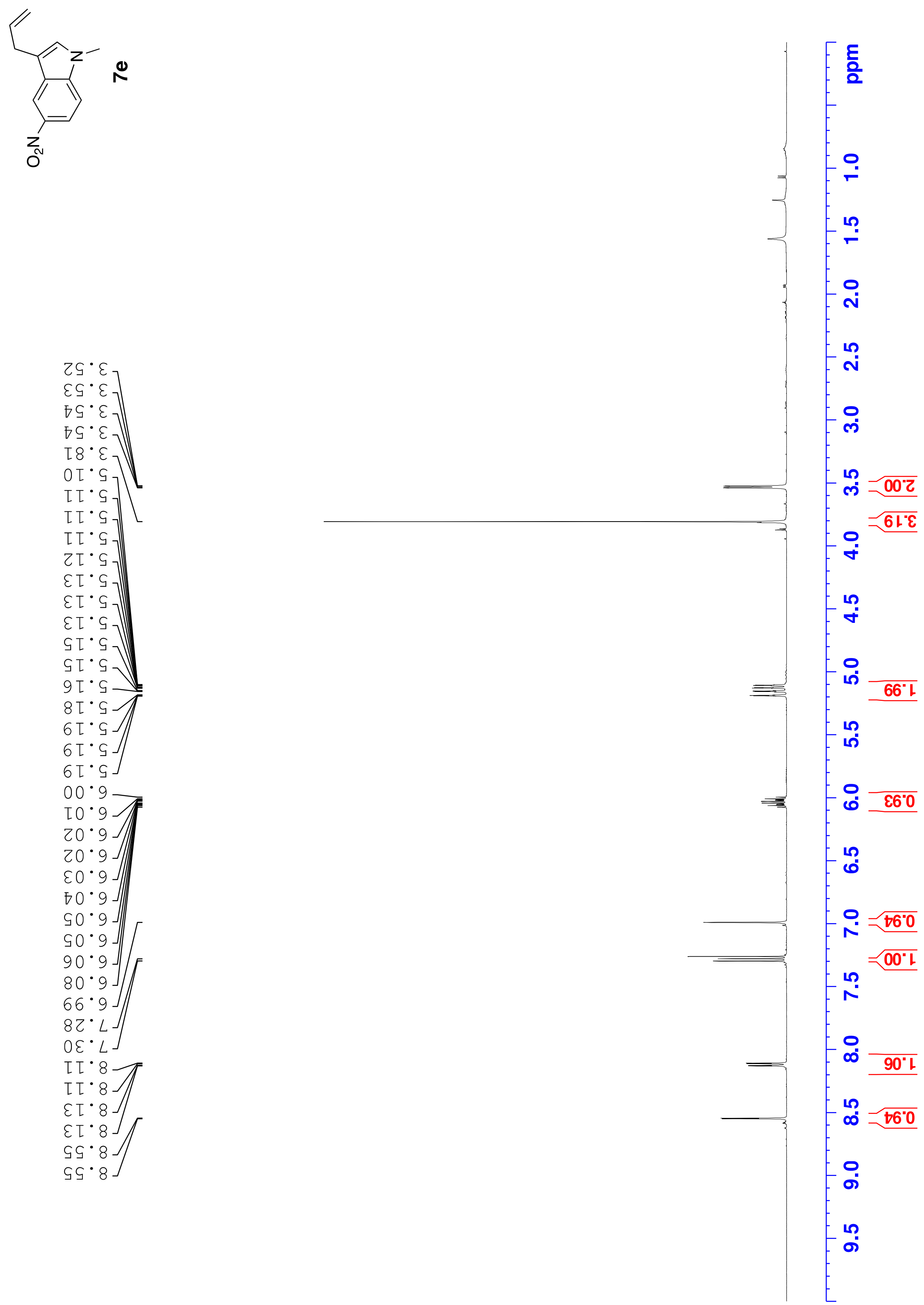
O2N
N
7e
3.52
3.53
3.54
3.54
3.81
5.10
5.11
5.11
5.11
5.12
5.13
5.13
5.13
5.15
5.15
5.16
5.18
5.19
5.19
5.19
6.00
6.01
6.02
6.02
6.03
6.04
6.05
6.05
6.06
6.08
6.99
7.28
7.30
8.11
8.11
8.13
8.13
8.55
8.55
2.00
3.19
1.99
0.93
0.94
1.00
1.06
0.94
ppm
1.0
1.5
2.0
2.5
3.0
3.5
4.0
4.5
5.0
5.5
6.0
6.5
7.0
7.5
8.0
8.5
9.0
9.5

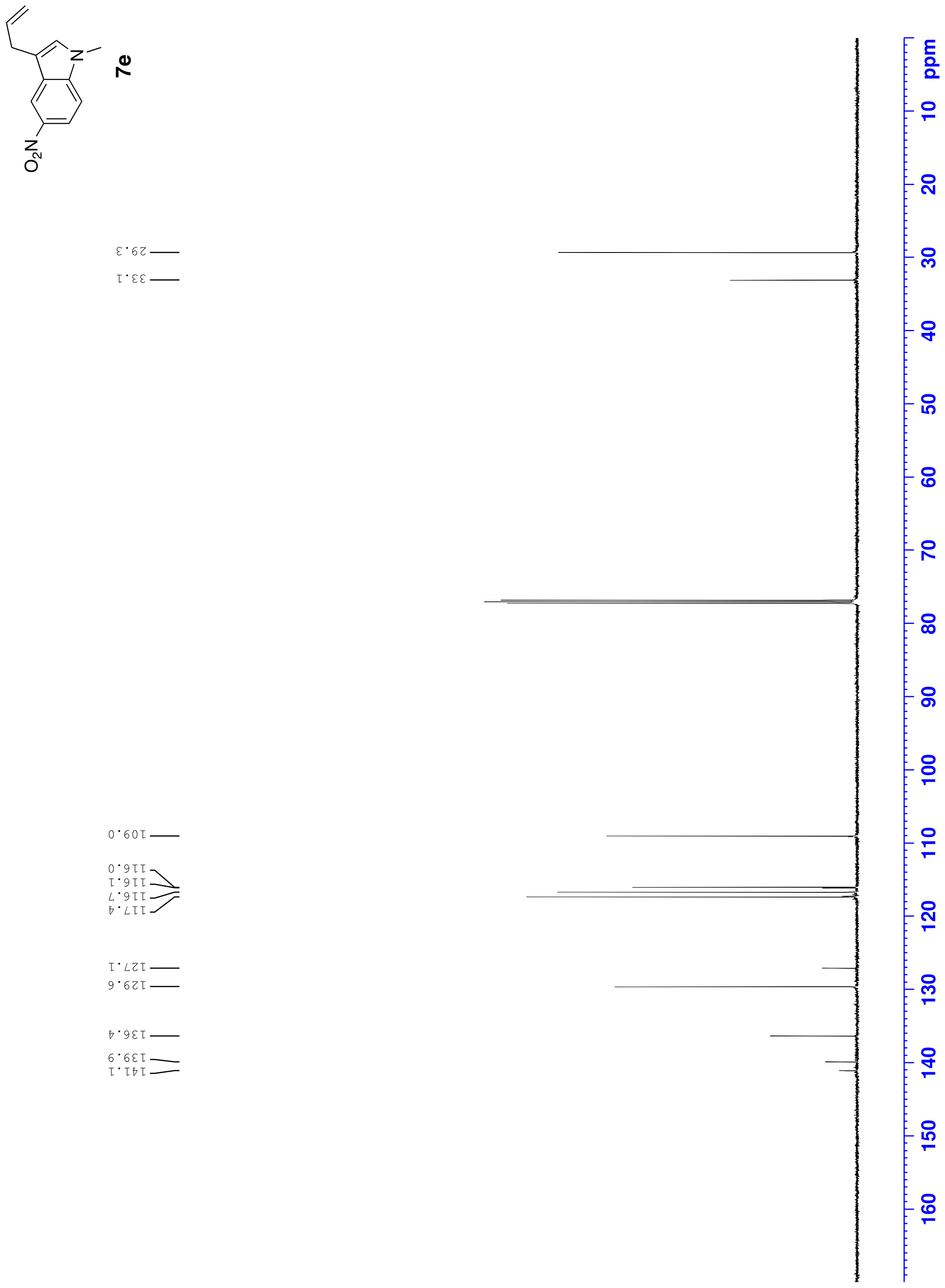
O2N
N
7e
29.3
33.1
109.0
116.0
116.1
116.7
117.4
127.1
129.6
136.4
139.9
141.1
ppm
10
20
30
40
50
60
70
80
90
100
110
120
130
140
150
160

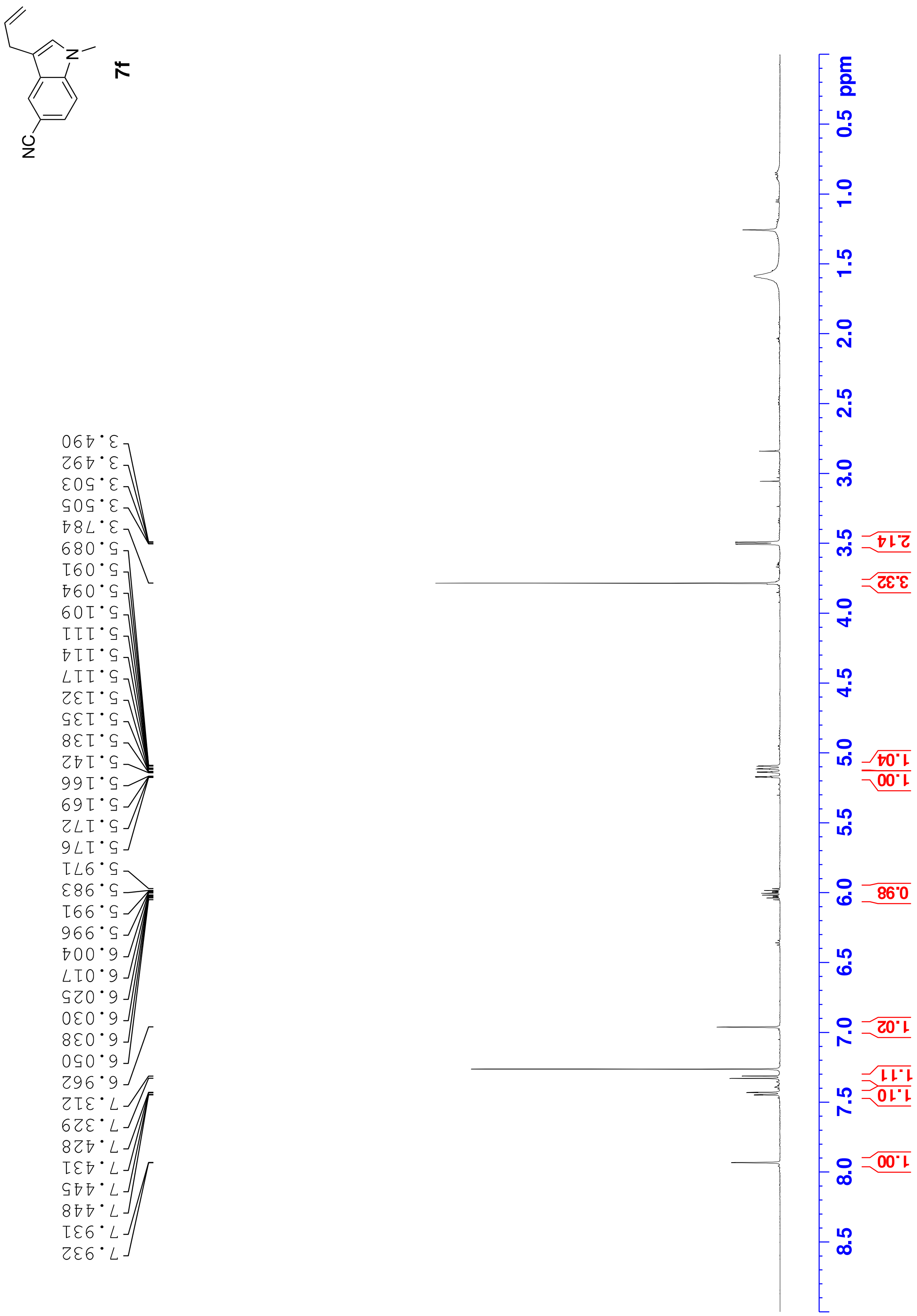
NC
N
7f
3.490
3.492
3.503
3.505
3.784
5.089
5.091
5.094
5.109
5.111
5.114
5.117
5.132
5.135
5.138
5.142
5.166
5.169
5.172
5.176
5.971
5.983
5.991
5.996
6.004
6.017
6.025
6.030
6.038
6.050
6.962
7.312
7.329
7.428
7.431
7.445
7.448
7.931
7.932
2.14
3.32
1.04
1.00
0.98
1.02
1.11
1.10
1.00
ppm

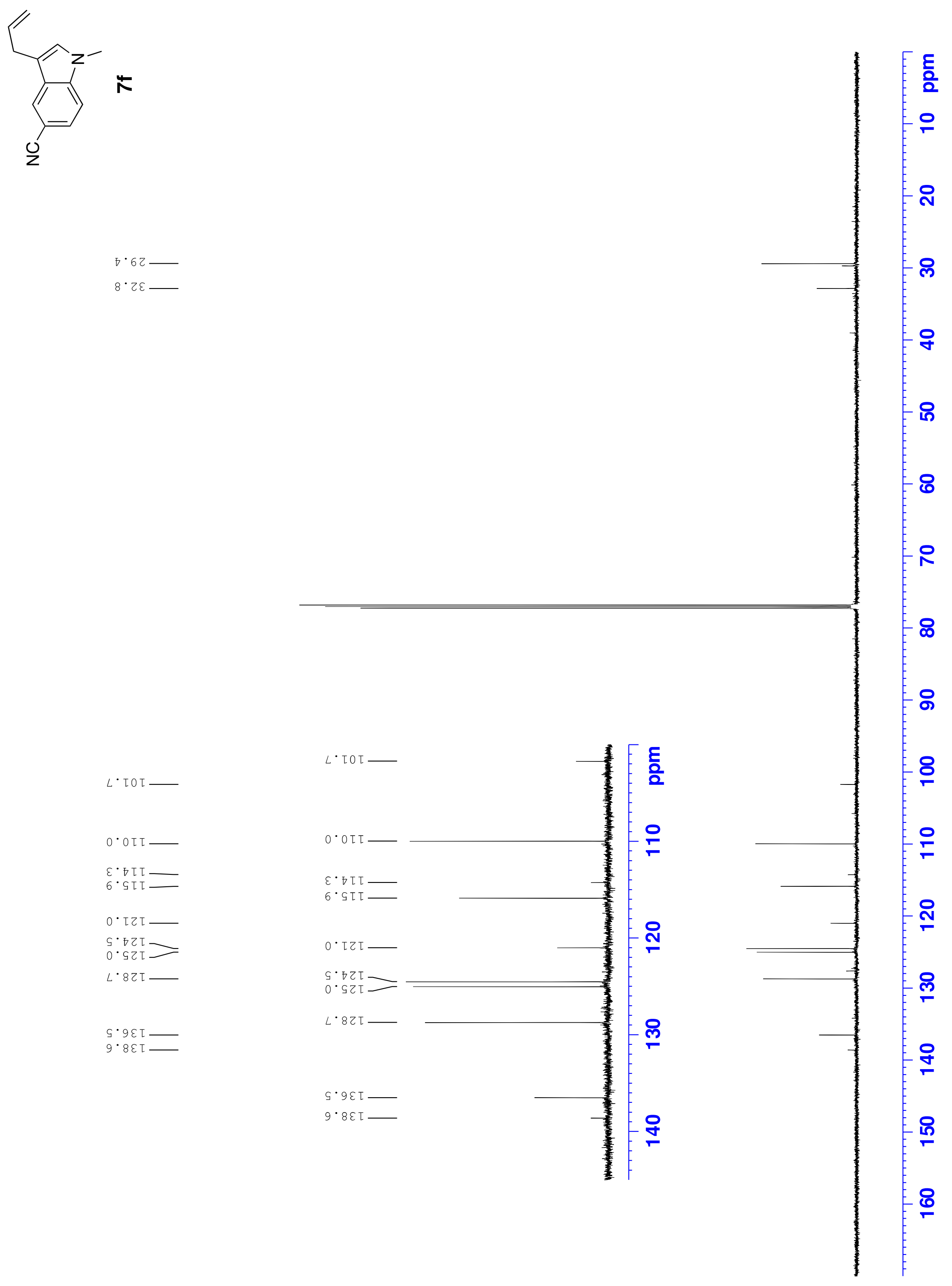
NC
N
7f
29.4
32.8
101.7
110.0
114.3
115.9
121.0
124.5
125.0
128.7
136.5
138.6
ppm

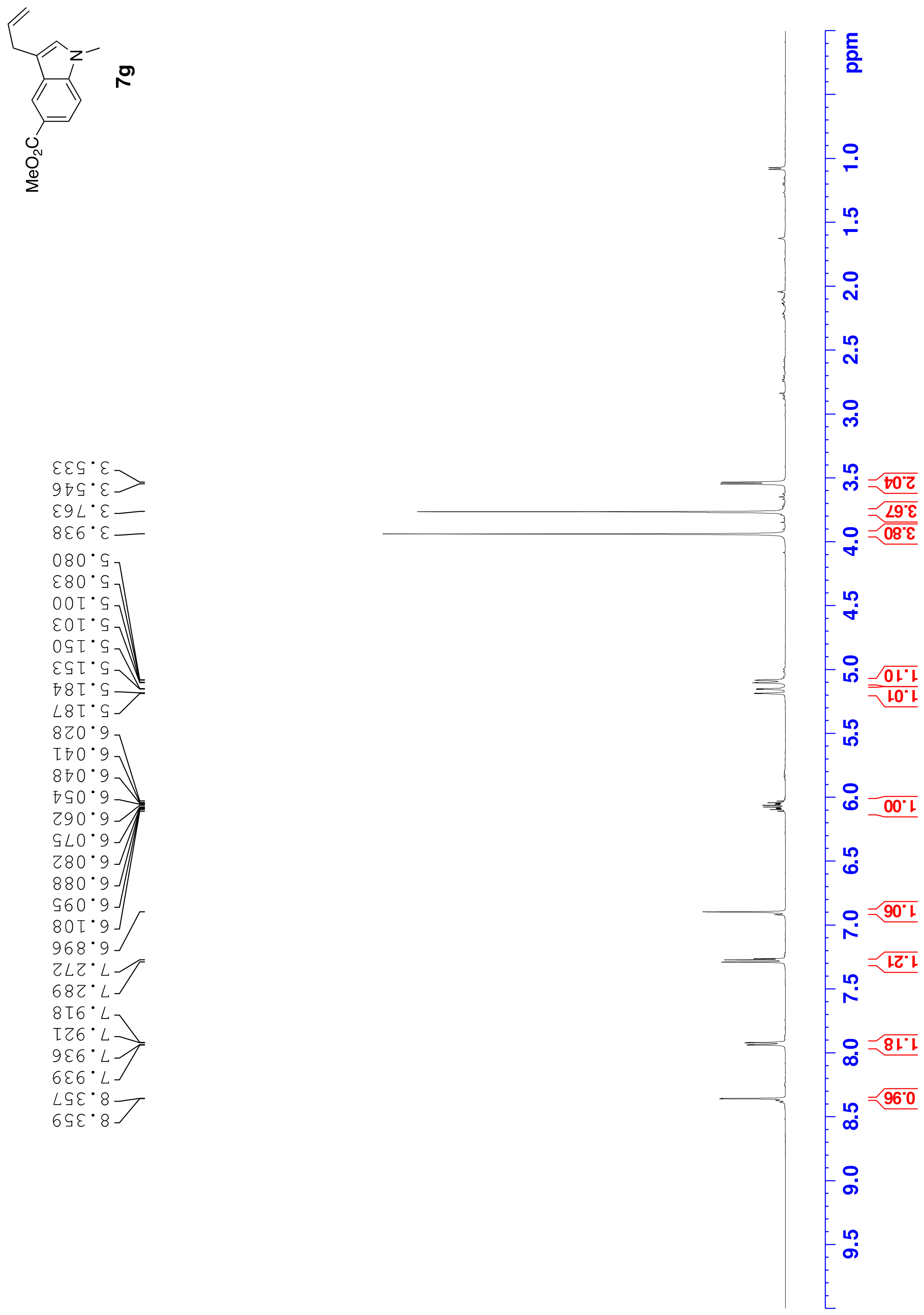
MeO2C
N
7g
3.533
3.546
3.763
3.938
5.080
5.083
5.100
5.103
5.150
5.153
5.184
5.187
6.028
6.041
6.048
6.054
6.062
6.075
6.082
6.088
6.095
6.108
6.896
7.272
7.289
7.918
7.921
7.936
7.939
8.357
8.359
2.04
3.67
3.80
1.10
1.01
1.00
1.06
1.21
1.18
0.96
ppm
1.0
1.5
2.0
2.5
3.0
3.5
4.0
4.5
5.0
5.5
6.0
6.5
7.0
7.5
8.0
8.5
9.0
9.5

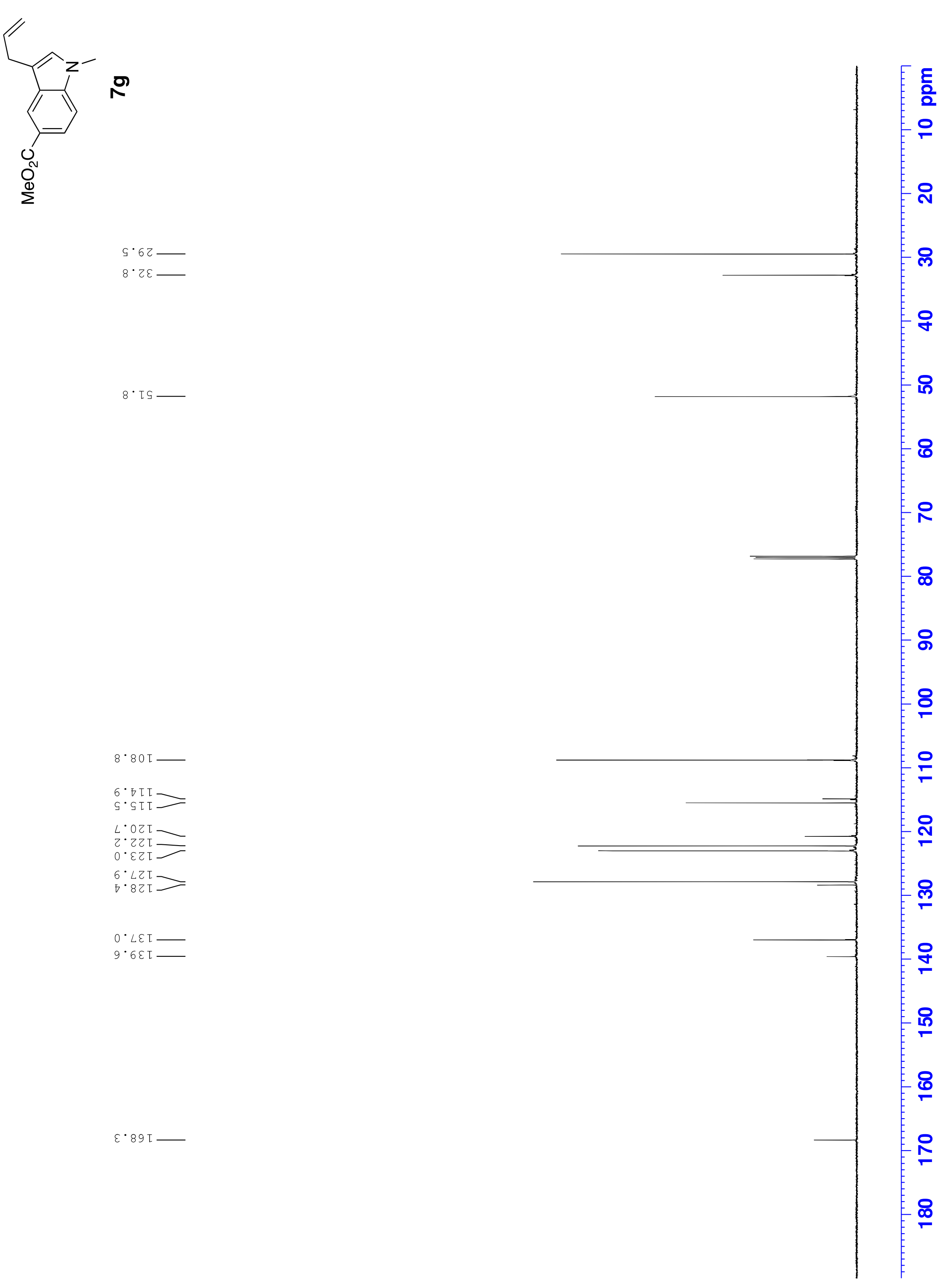
MeO2C
N
7g
29.5
32.8
51.8
108.8
114.9
115.5
120.7
122.2
123.0
127.9
128.4
137.0
139.6
168.3
ppm
10
20
30
40
50
60
70
80
90
100
110
120
130
140
150
160
170
180

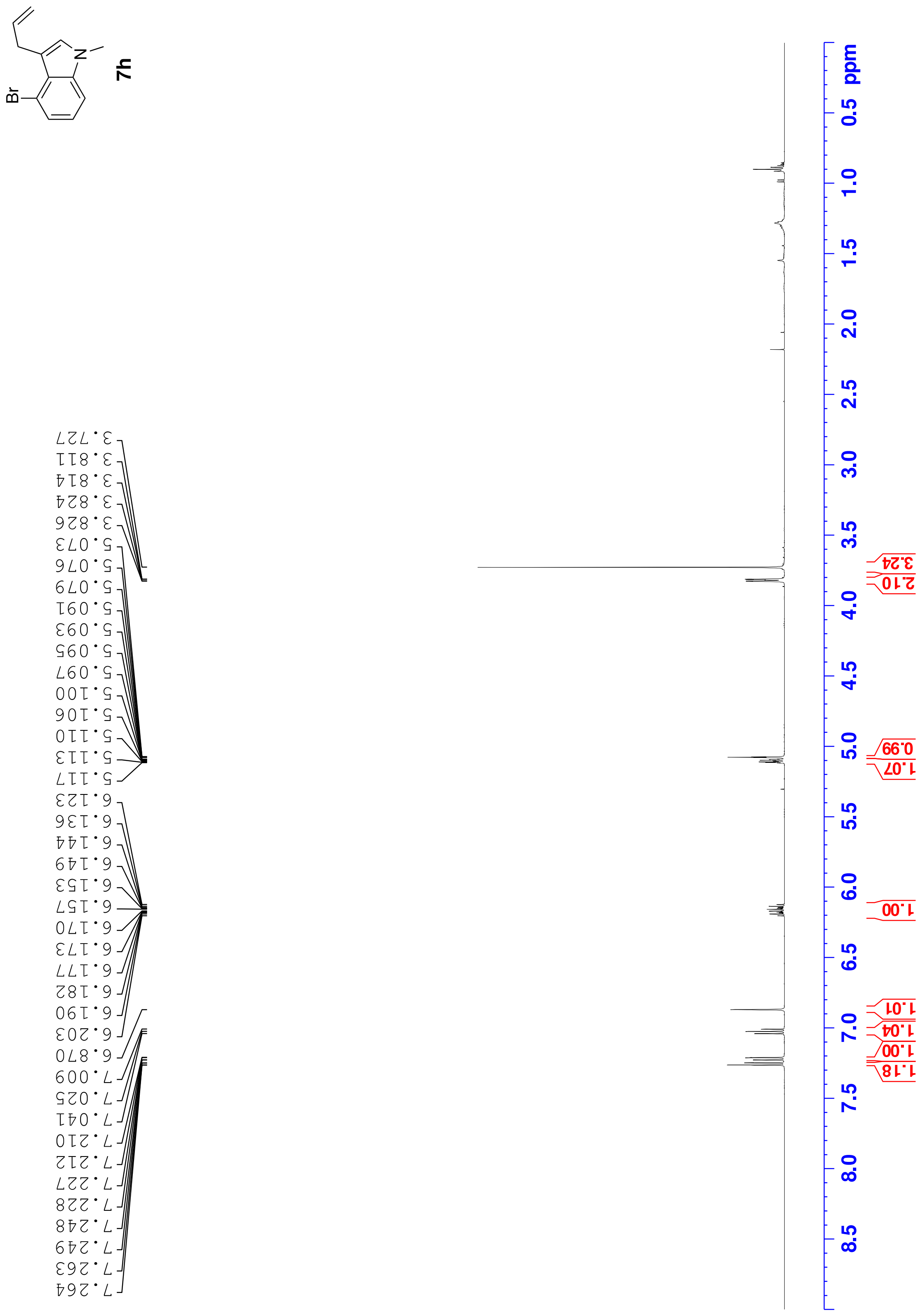

Br
N
7h
3.727
3.811
3.814
3.824
3.826
5.073
5.076
5.079
5.091
5.093
5.095
5.097
5.100
5.106
5.110
5.113
5.117
6.123
6.136
6.144
6.149
6.153
6.157
6.170
6.173
6.177
6.182
6.190
6.203
6.870
7.009
7.025
7.041
7.210
7.212
7.227
7.228
7.248
7.249
7.263
7.264
3.24
2.10
0.99
1.07
1.00
1.01
1.04
1.00
1.18
ppm
0.5
1.0
1.5
2.0
2.5
3.0
3.5
4.0
4.5
5.0
5.5
6.0
6.5
7.0
7.5
8.0
8.5

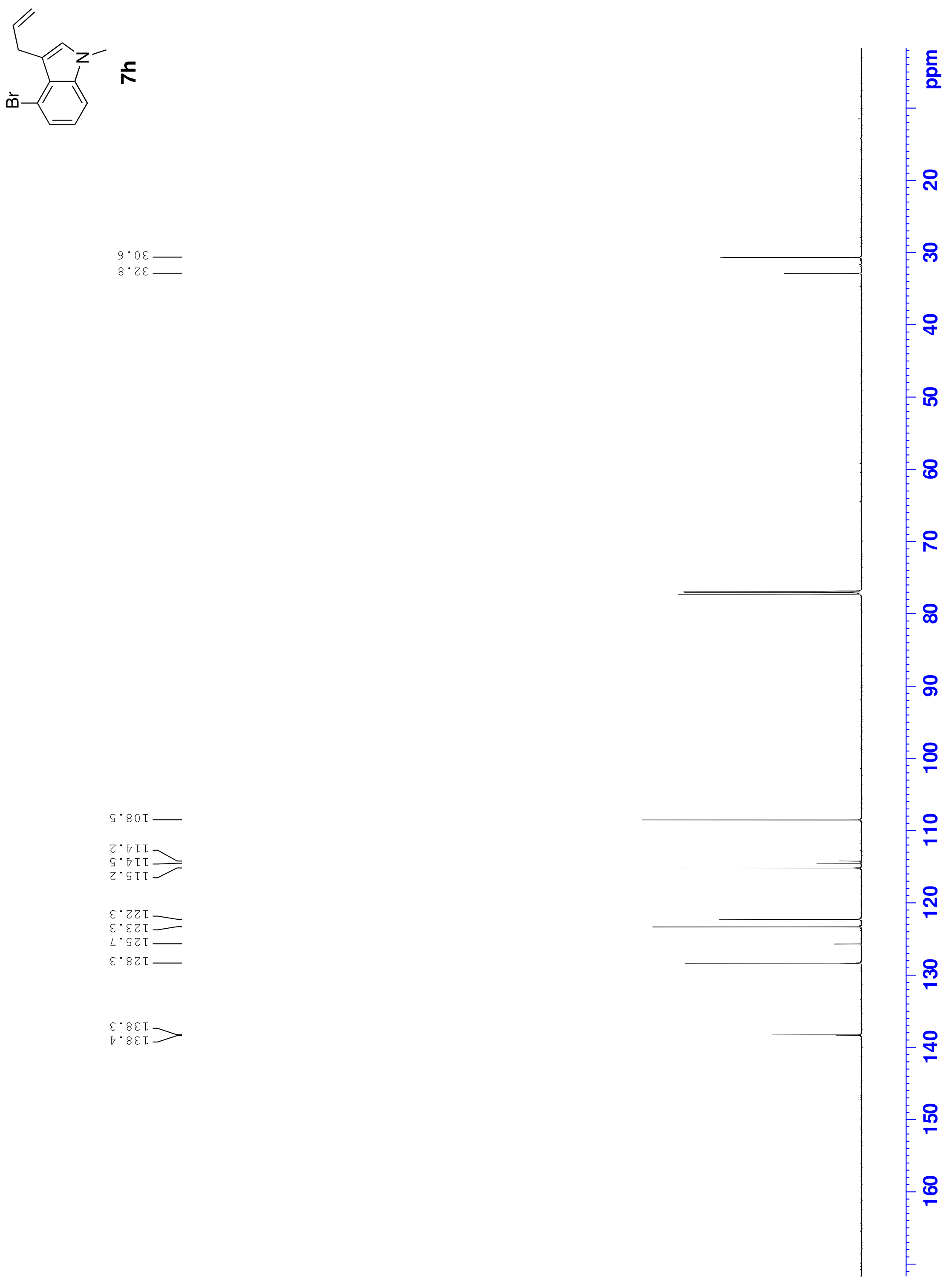
Br
N
7h
30.6
32.8
108.5
114.2
114.5
115.2
122.3
123.3
125.7
128.3
138.3
138.4
ppm
20
30
40
50
60
70
80
90
100
110
120
130
140
150
160

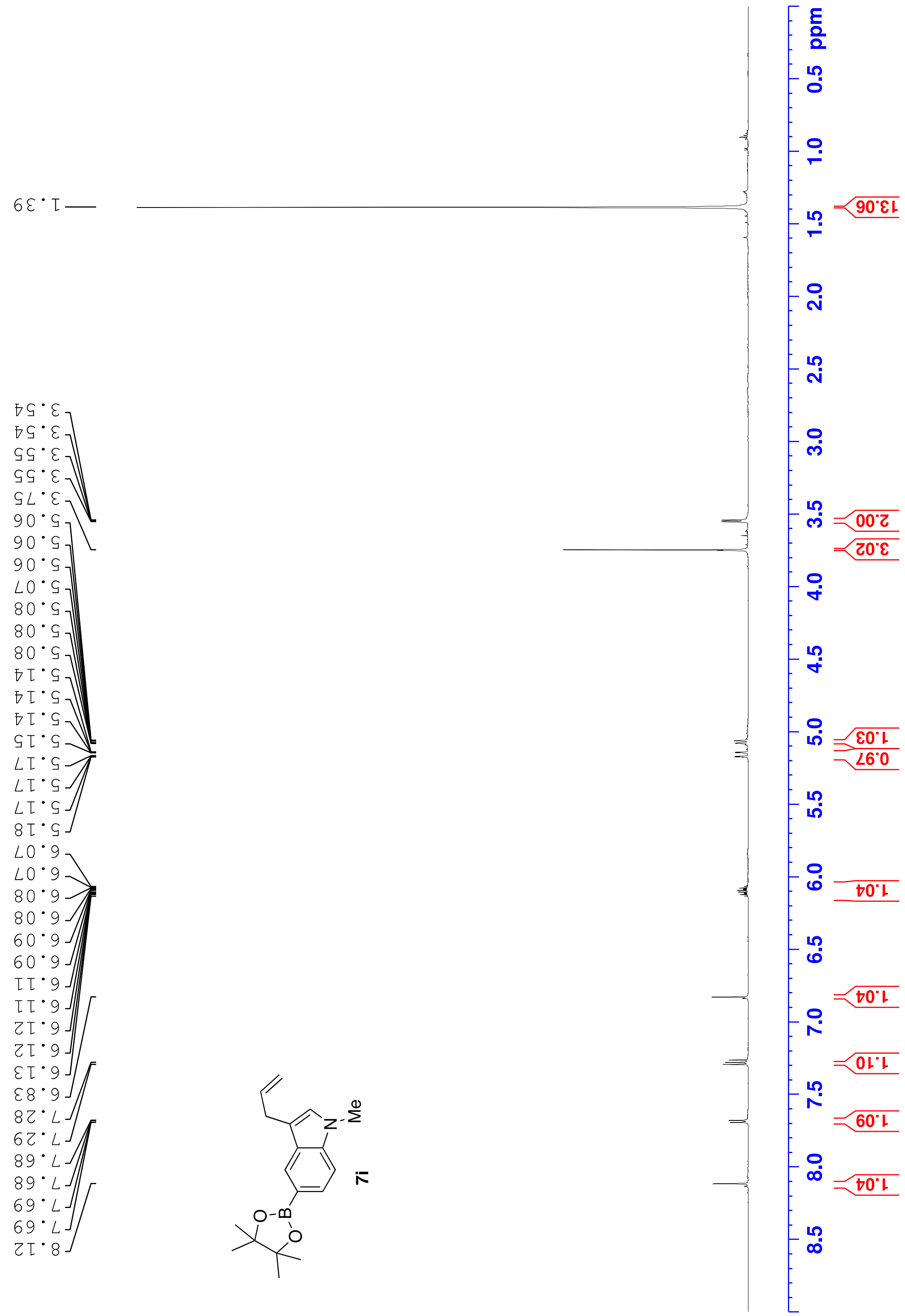
1.39
3.54
3.54
3.55
3.55
3.75
5.06
5.06
5.06
5.07
5.08
5.08
5.08
5.14
5.14
5.14
5.15
5.17
5.17
5.17
5.18
6.07
6.07
6.08
6.08
6.09
6.09
6.11
6.11
6.12
6.12
6.13
6.83
7.28
7.29
7.68
7.68
7.69
7.69
8.12
N
Me
B
O
O
7i
ppm
0.5
1.0
1.5
2.0
2.5
3.0
3.5
4.0
4.5
5.0
5.5
6.0
6.5
7.0
7.5
8.0
8.5
13.06
2.00
3.02
1.03
0.97
1.04
1.04
1.10
1.09
1.04

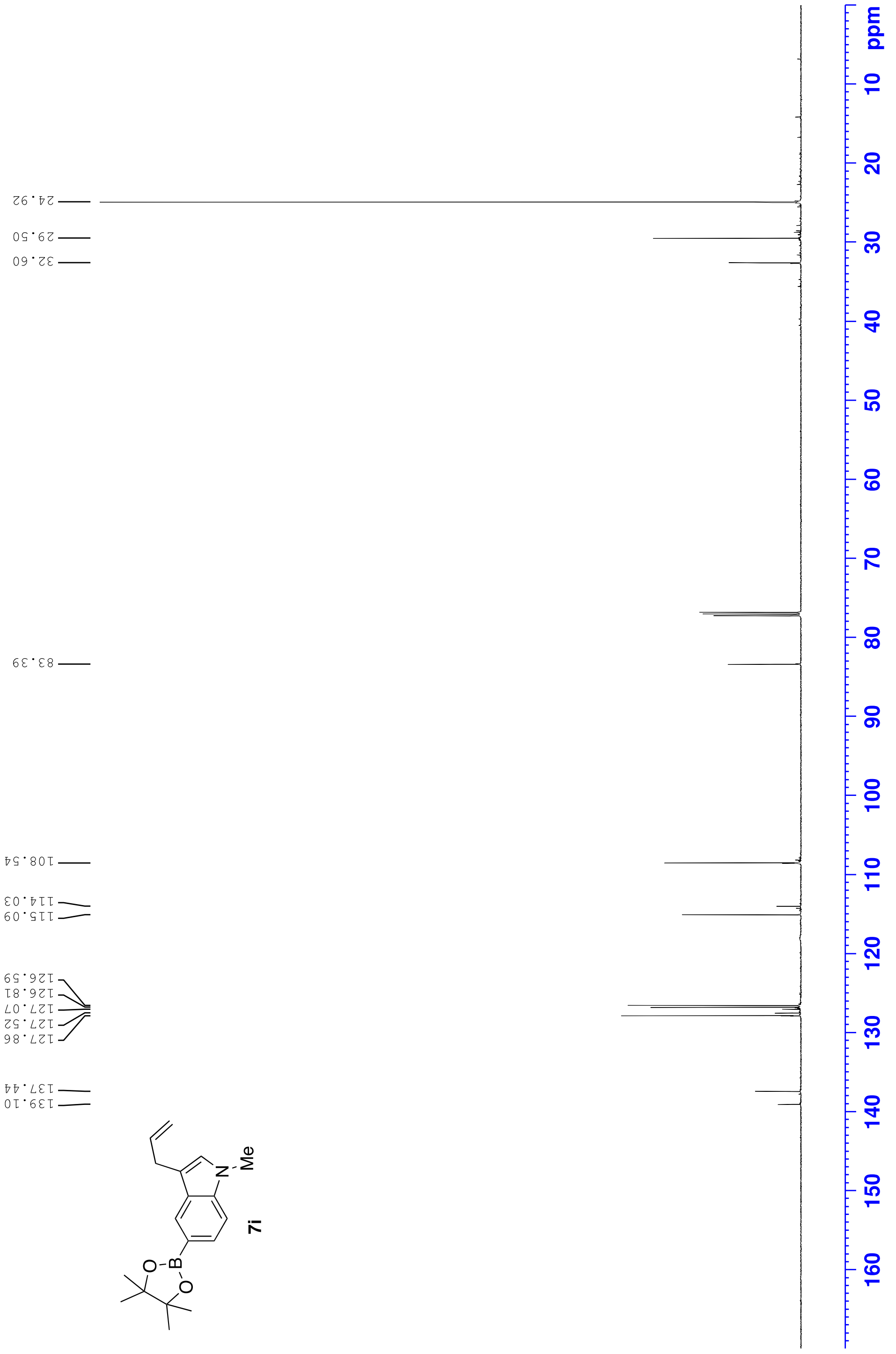
24.92
29.50
32.60
83.39
108.54
114.03
115.09
126.59
126.81
127.07
127.52
127.86
137.44
139.10
7i
ppm

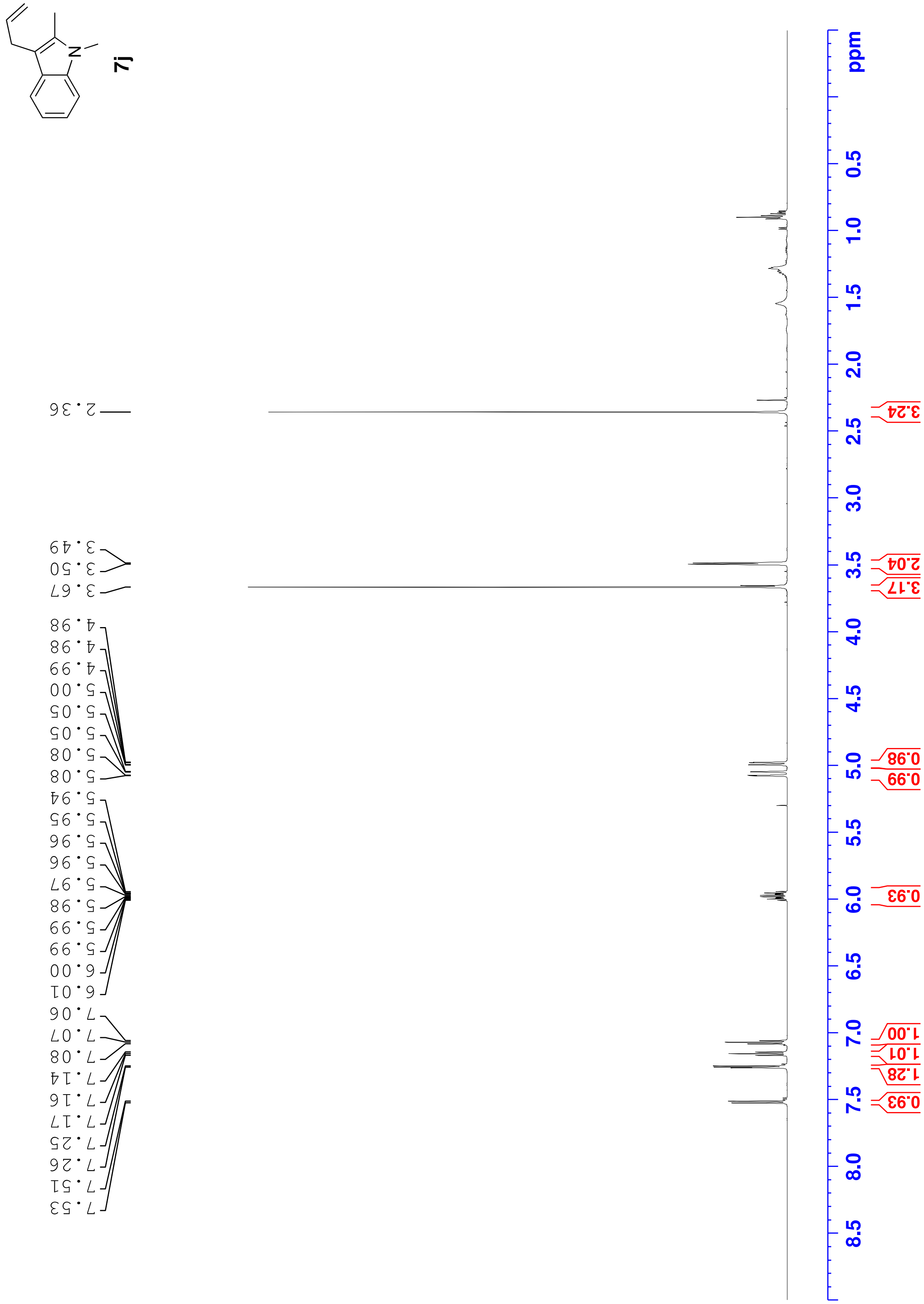
7j
2.36
3.49
3.50
3.67
4.98
4.98
4.99
5.00
5.05
5.05
5.08
5.08
5.94
5.95
5.96
5.96
5.97
5.98
5.99
5.99
6.00
6.01
7.06
7.07
7.08
7.14
7.16
7.17
7.25
7.26
7.51
7.53
3.24
2.04
3.17
0.98
0.99
0.93
1.00
1.01
1.28
0.93
ppm
0.5
1.0
1.5
2.0
2.5
3.0
3.5
4.0
4.5
5.0
5.5
6.0
6.5
7.0
7.5
8.0
8.5

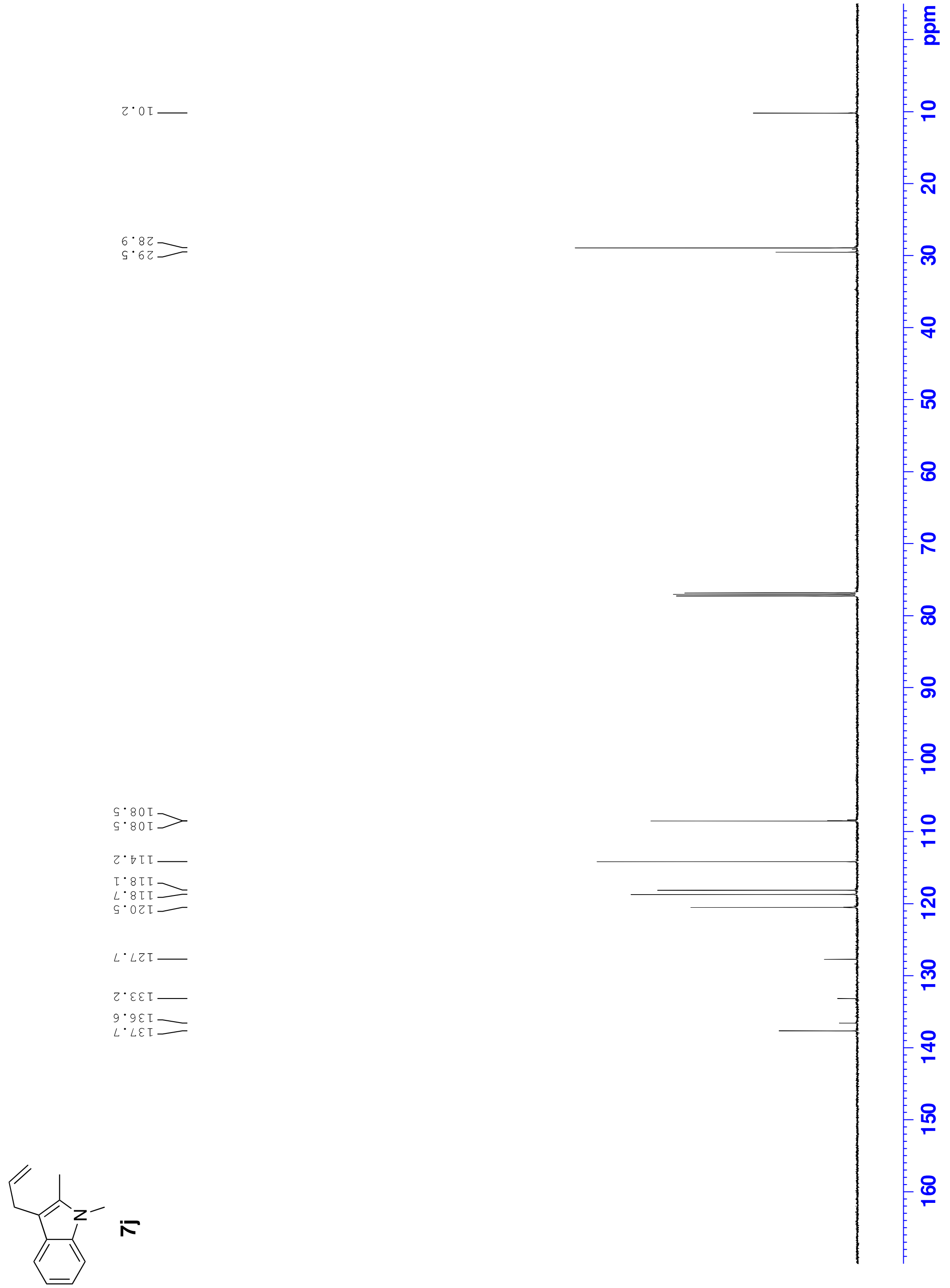

10.2
28.9
29.5
108.5
108.5
114.2
118.1
118.7
120.5
127.7
133.2
136.6
137.7
7j
ppm
10
20
30
40
50
60
70
80
90
100
110
120
130
140
150
160

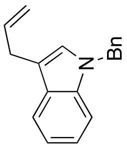
N
Bn

7k

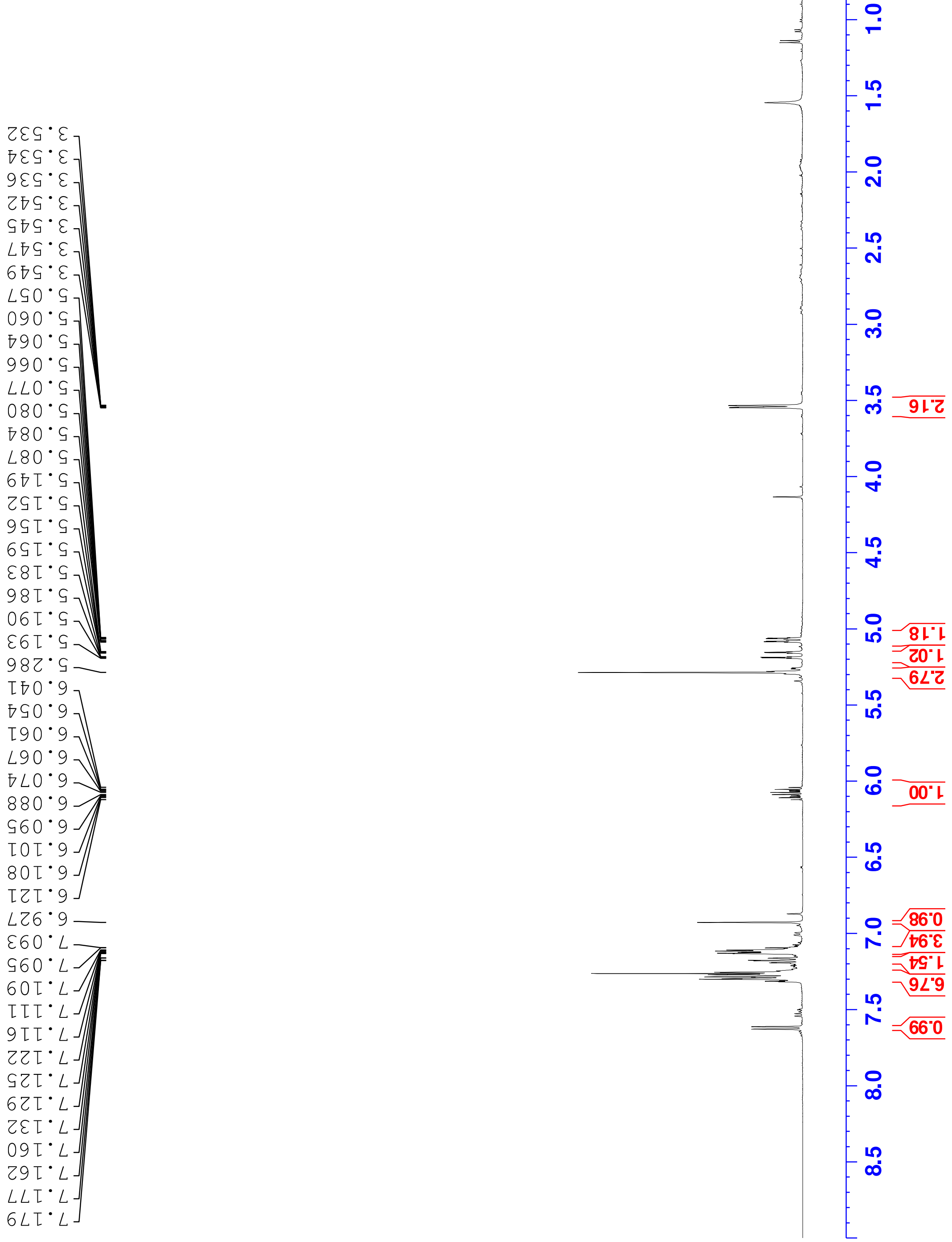
3.532
3.534
3.536
3.542
3.545
3.547
3.549
5.057
5.060
5.064
5.066
5.077
5.080
5.084
5.087
5.149
5.152
5.156
5.159
5.183
5.186
5.190
5.193
5.286
6.041
6.054
6.061
6.067
6.074
6.088
6.095
6.101
6.108
6.121
6.927
7.093
7.095
7.109
7.111
7.116
7.122
7.125
7.129
7.132
7.160
7.162
7.177
7.179
2.16
1.18
1.02
2.79
1.00
0.98
3.94
1.54
6.76
0.99
ppm
0.5
1.0
1.5
2.0
2.5
3.0
3.5
4.0
4.5
5.0
5.5
6.0
6.5
7.0
7.5
8.0
8.5

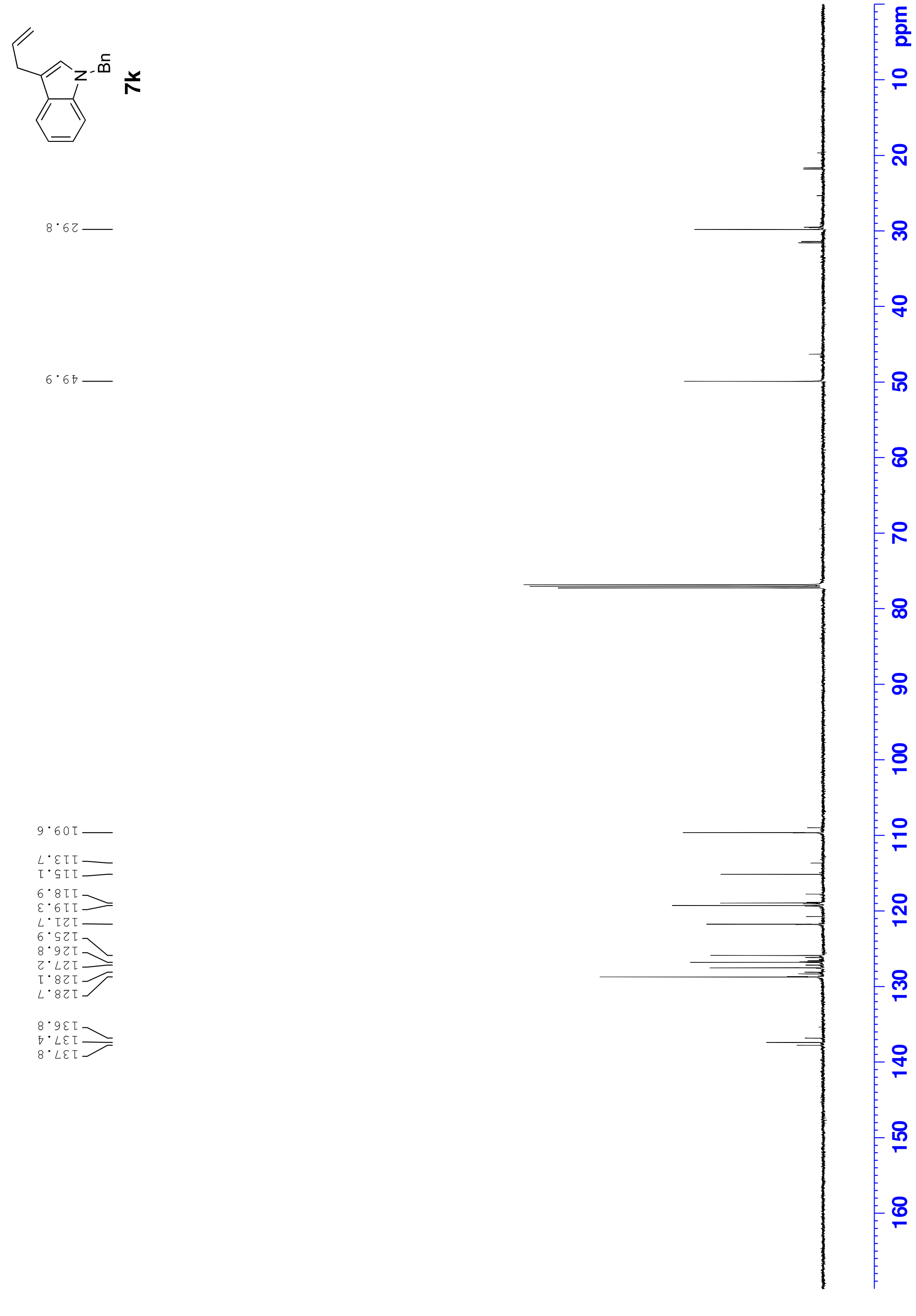
N
Bn
7k
29.8
49.9
109.6
113.7
115.1
118.9
119.3
121.7
125.9
126.8
127.2
128.1
128.7
136.8
137.4
137.8
ppm
10
20
30
40
50
60
70
80
90
100
110
120
130
140
150
160

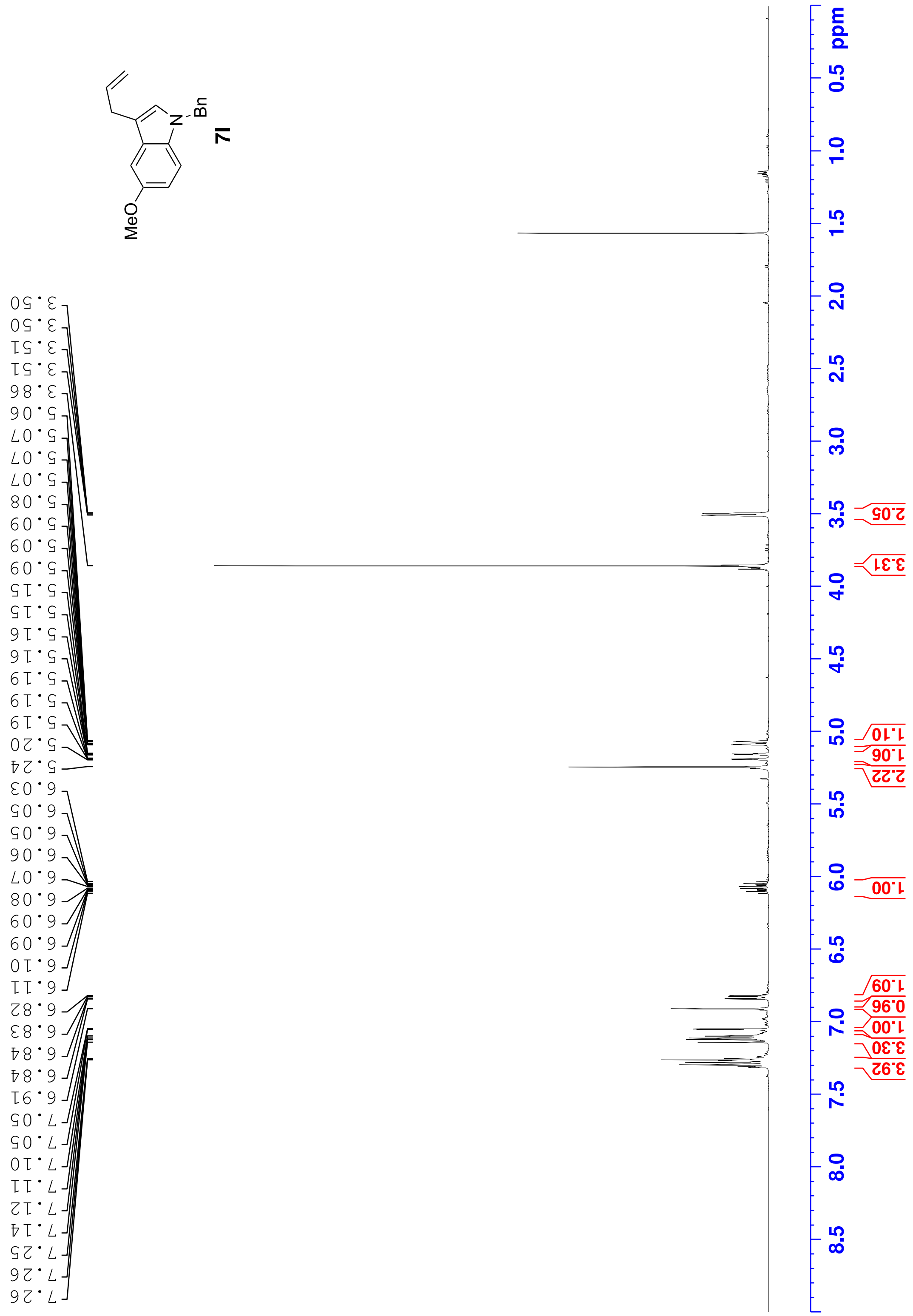
MeO
N
Bn
7l
3.50
3.50
3.51
3.51
3.86
5.06
5.07
5.07
5.07
5.08
5.09
5.09
5.09
5.15
5.15
5.16
5.16
5.19
5.19
5.19
5.20
5.24
6.03
6.05
6.05
6.06
6.07
6.08
6.09
6.09
6.10
6.11
6.82
6.83
6.84
6.84
6.91
7.05
7.05
7.10
7.11
7.12
7.14
7.25
7.26
7.26
2.05
3.31
1.10
1.06
2.22
1.00
1.09
0.96
1.00
3.30
3.92
0.5
1.0
1.5
2.0
2.5
3.0
3.5
4.0
4.5
5.0
5.5
6.0
6.5
7.0
7.5
8.0
8.5
ppm

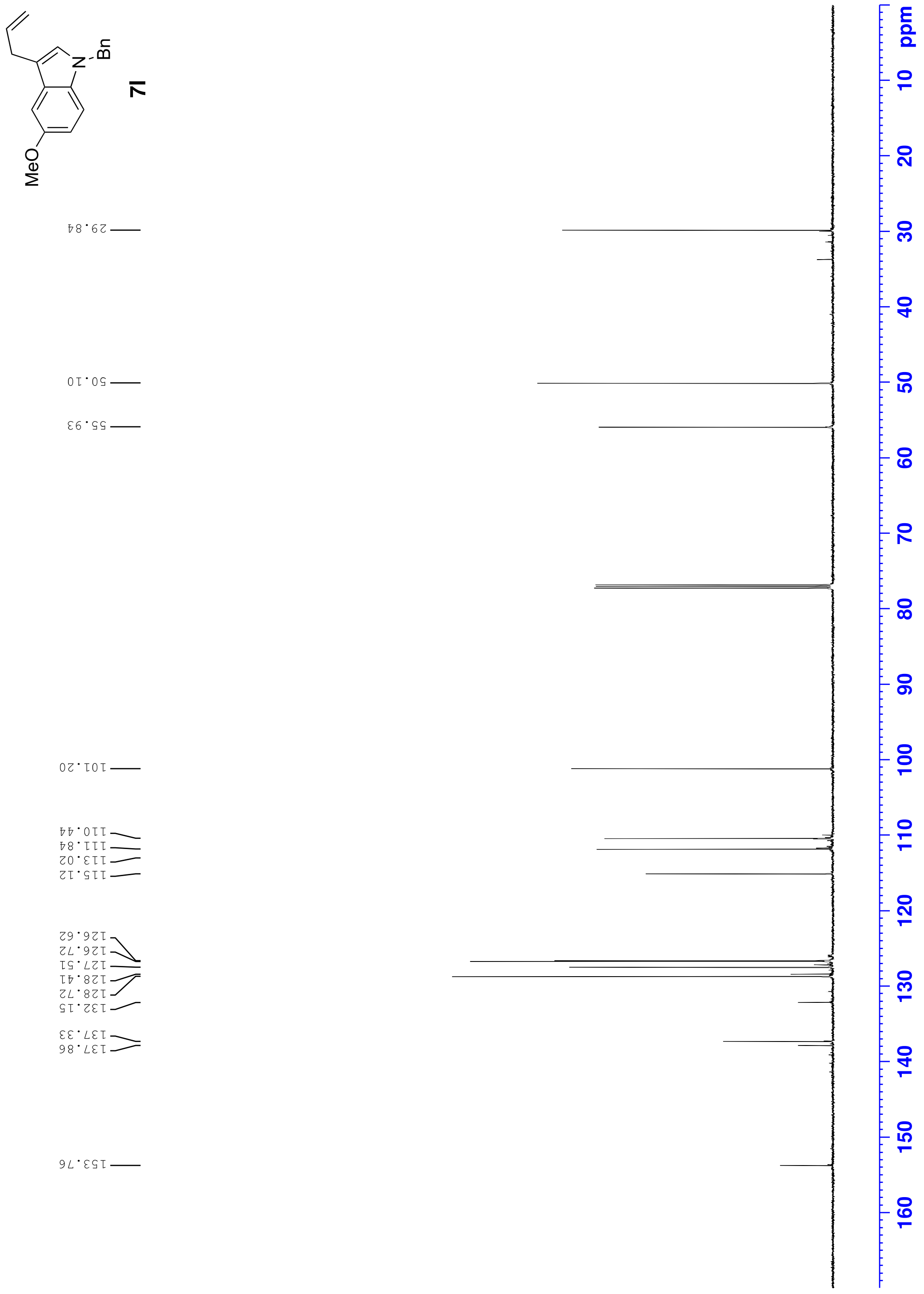
MeO
N
Bn
7l
29.84
50.10
55.93
101.20
110.44
111.84
113.02
115.12
126.62
126.72
127.51
128.41
128.72
132.15
137.33
137.86
153.76
ppm
10
20
30
40
50
60
70
80
90
100
110
120
130
140
150
160

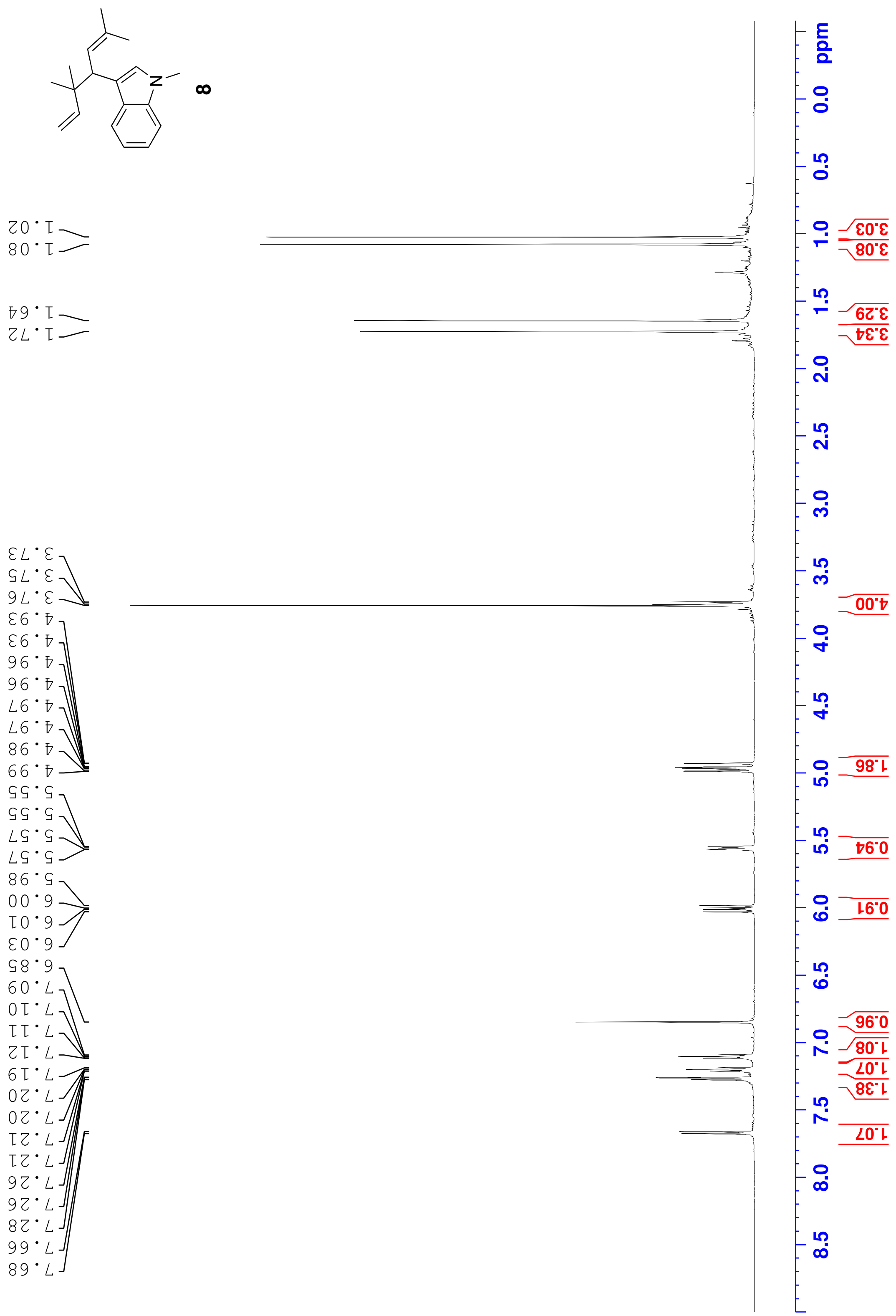
8
ppm
1.02
1.08
1.64
1.72
3.73
3.75
3.76
4.93
4.93
4.96
4.96
4.97
4.97
4.98
4.99
5.55
5.55
5.57
5.57
5.98
6.00
6.01
6.03
6.85
7.09
7.10
7.11
7.12
7.19
7.20
7.20
7.21
7.21
7.26
7.26
7.28
7.66
7.68
3.03
3.08
3.29
3.34
4.00
1.86
0.94
0.91
0.96
1.08
1.07
1.38
1.07

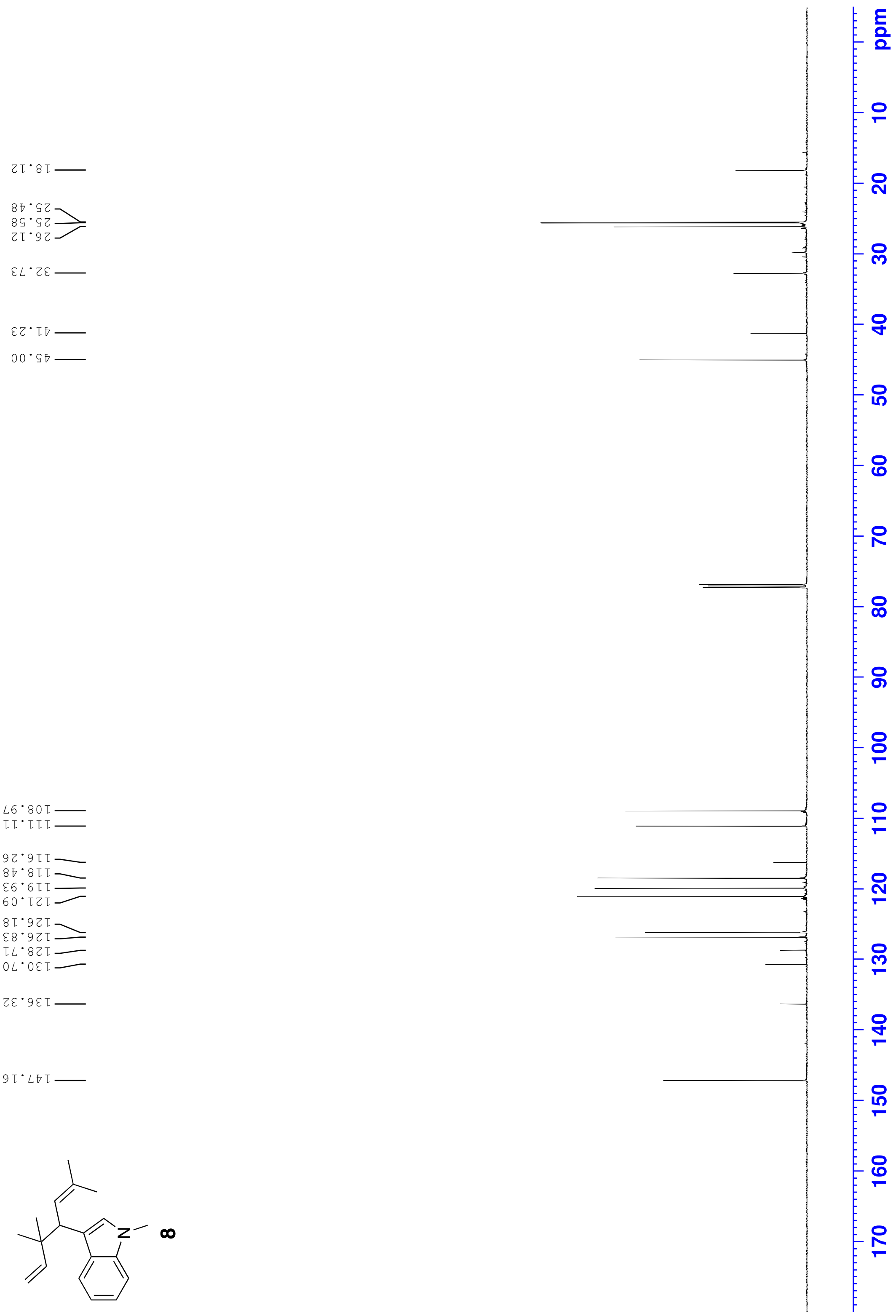

18.12
25.48
25.58
26.12
32.73
41.23
45.00
108.97
111.11
116.26
118.48
119.93
121.09
126.18
126.83
128.71
130.70
136.32
147.16
N
8
ppm
10
20
30
40
50
60
70
80
90
100
110
120
130
140
150
160
170

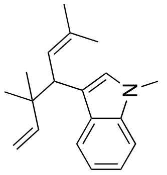

8

COSY

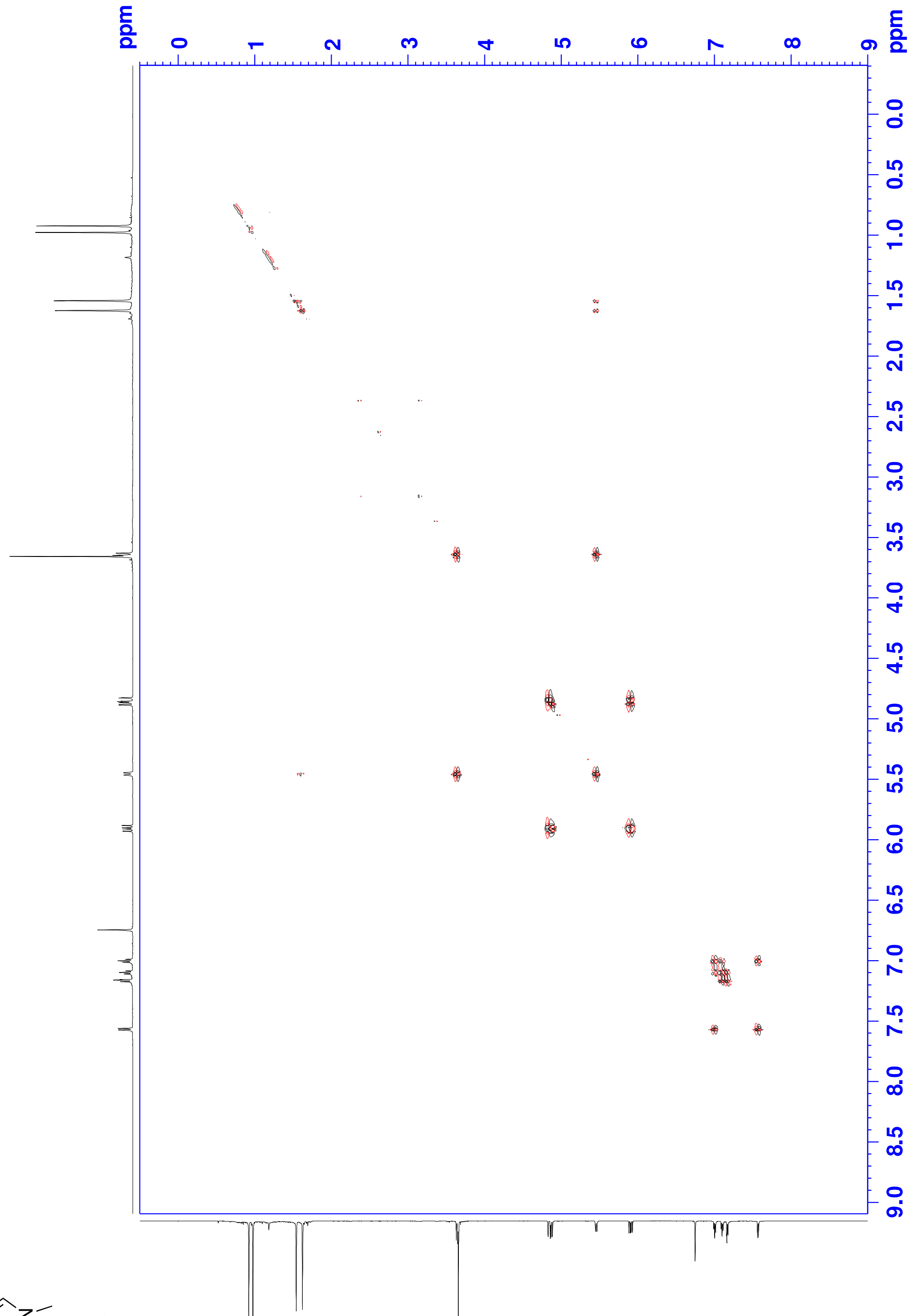

ppm
0
1
2
3
4
5
6
7
8
9
ppm
0.0
0.5
1.0
1.5
2.0
2.5
3.0
3.5
4.0
4.5
5.0
5.5
6.0
6.5
7.0
7.5
8.0
8.5
9.0

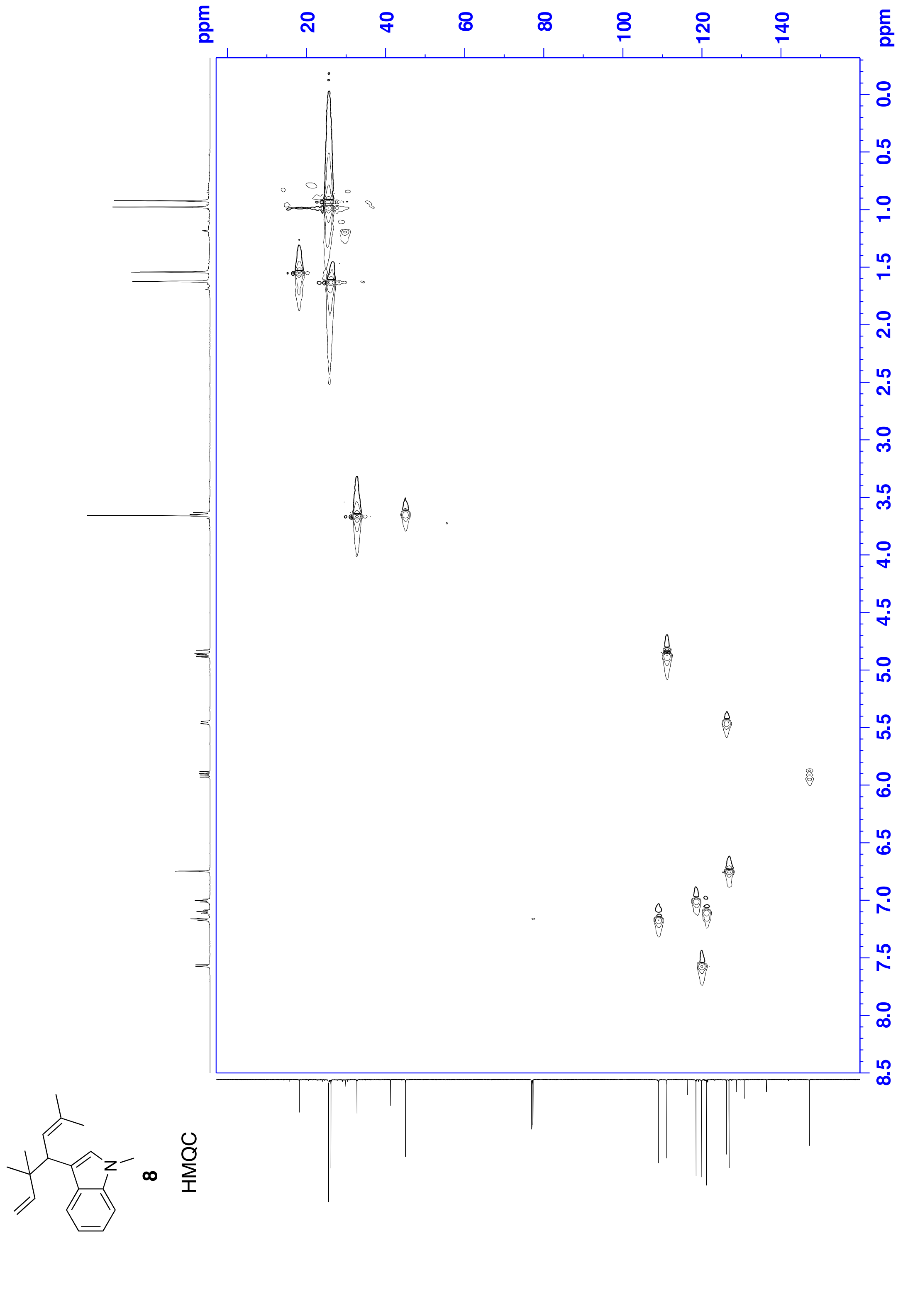
8
HMQC
ppm
20
40
60
80
100
120
140
ppm
0.0
0.5
1.0
1.5
2.0
2.5
3.0
3.5
4.0
4.5
5.0
5.5
6.0
6.5
7.0
7.5
8.0
8.5

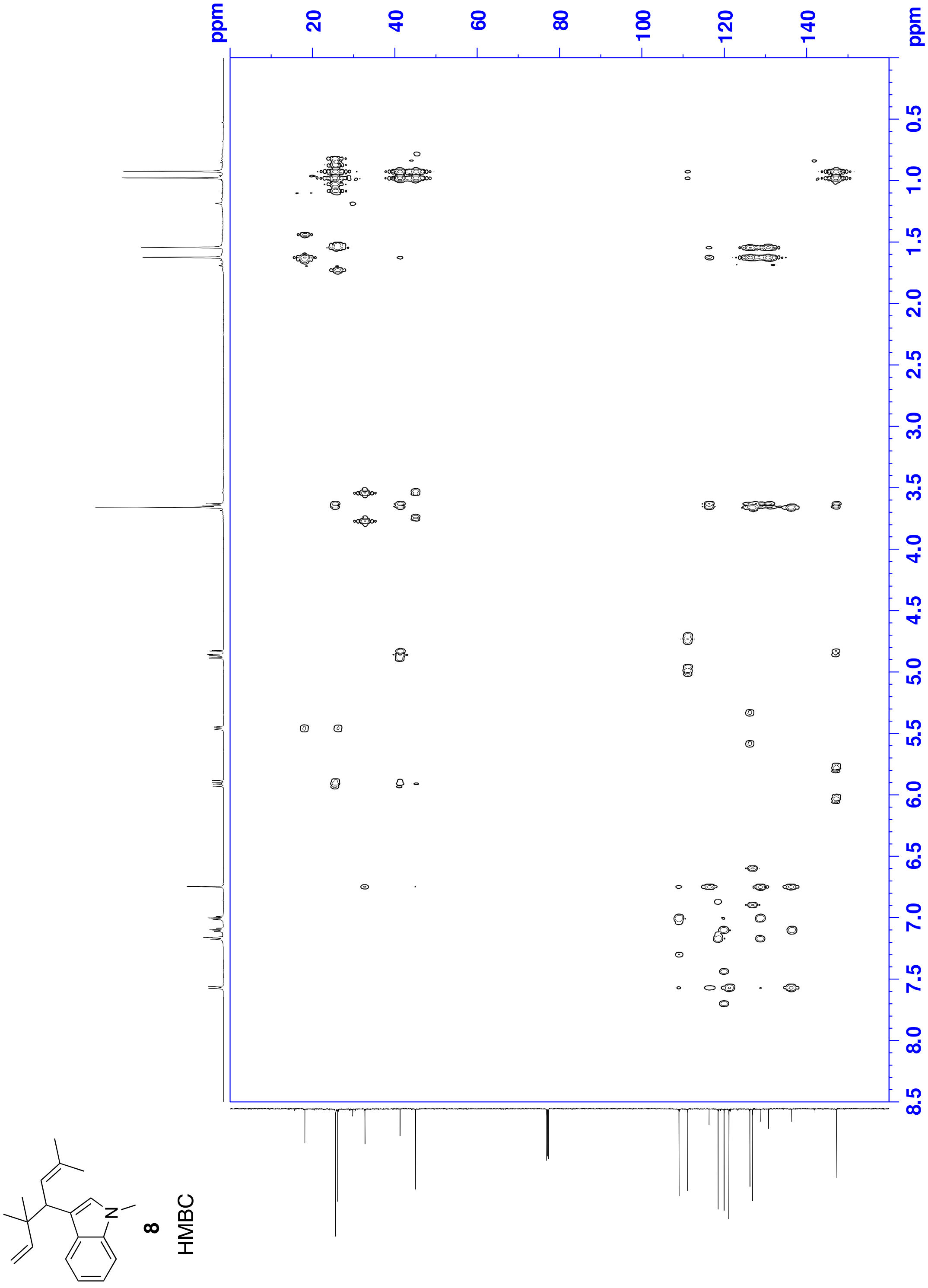

8

HMBC

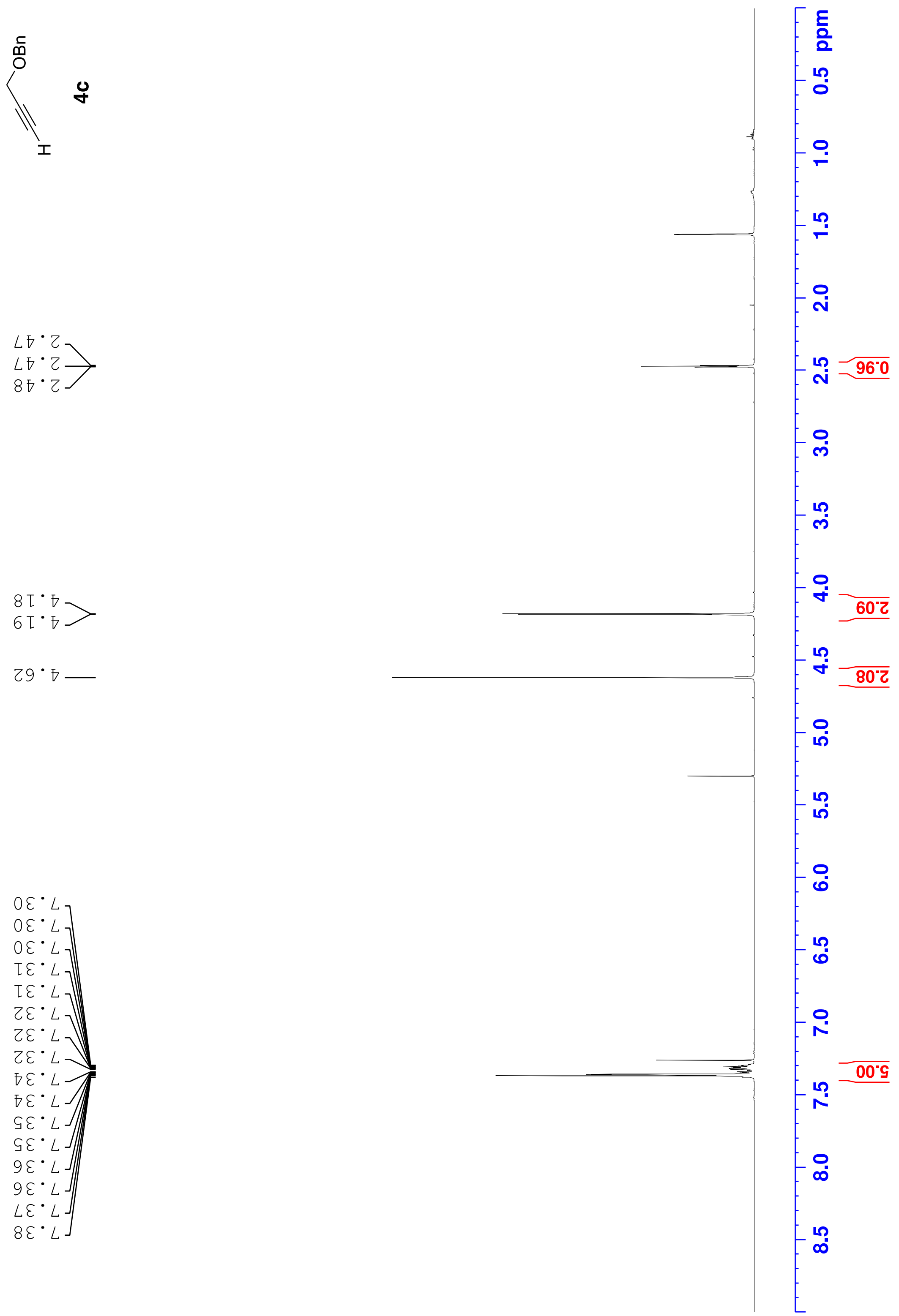

H
OBn
4c
7.38
7.37
7.36
7.36
7.35
7.35
7.34
7.34
7.32
7.32
7.32
7.31
7.31
7.30
7.30
7.30
4.62
4.19
4.18
2.48
2.47
2.47
5.00
2.08
2.09
0.96
ppm

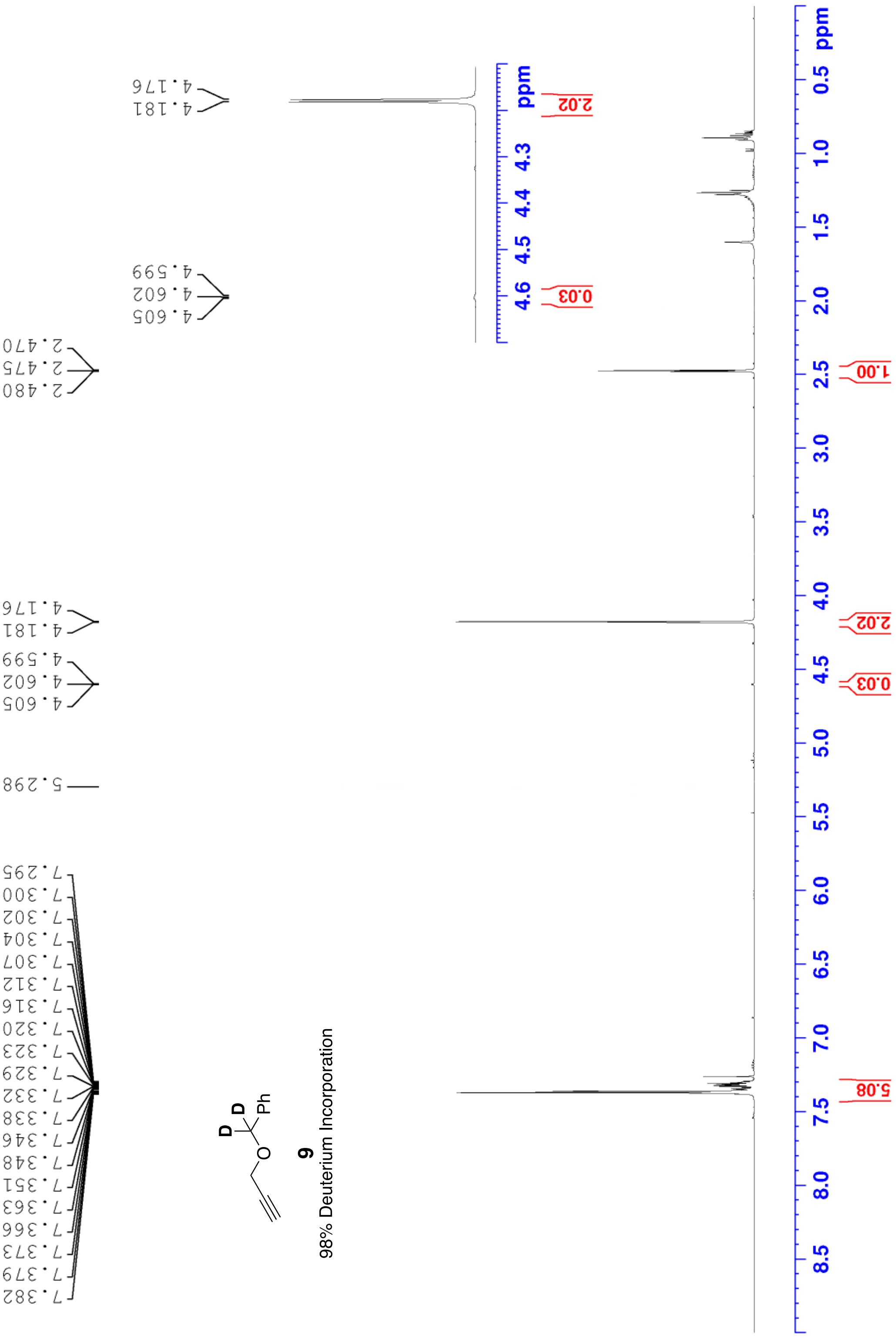

7.382
7.379
7.373
7.366
7.363
7.351
7.348
7.346
7.338
7.332
7.329
7.323
7.320
7.316
7.312
7.307
7.304
7.302
7.300
7.295
5.298
4.605
4.602
4.599
4.181
4.176
2.480
2.475
2.470
4.605
4.602
4.599
4.181
4.176
D
D
Ph
O
9
98% Deuterium Incorporation
ppm
4.6
4.5
4.4
4.3
0.03
2.02
5.08
0.03
2.02
1.00
8.5
8.0
7.5
7.0
6.5
6.0
5.5
5.0
4.5
4.0
3.5
3.0
2.5
2.0
1.5
1.0
0.5
ppm

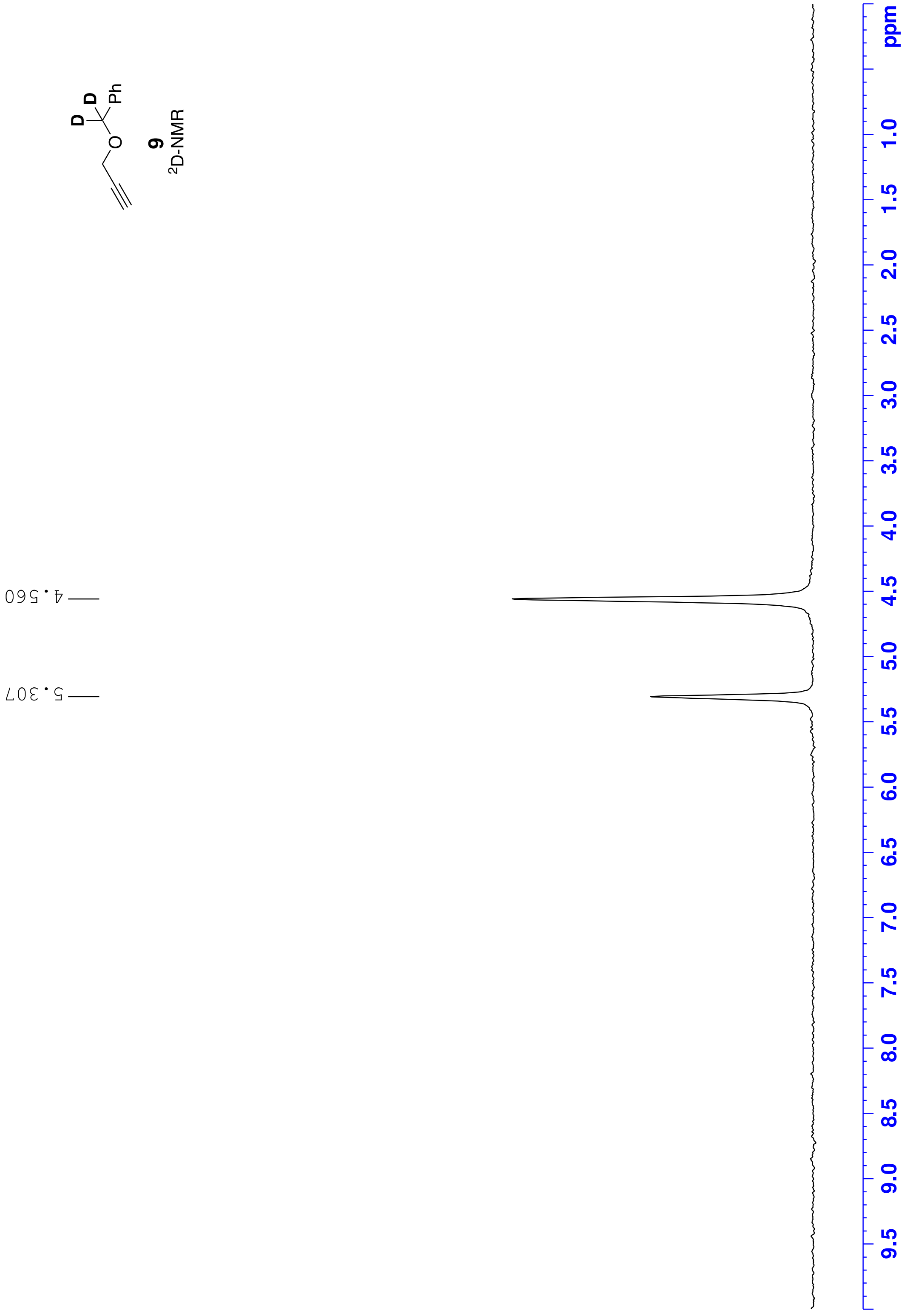
D
D
O
Ph
9
2D-NMR
4.560
5.307
ppm
1.0
1.5
2.0
2.5
3.0
3.5
4.0
4.5
5.0
5.5
6.0
6.5
7.0
7.5
8.0
8.5
9.0
9.5

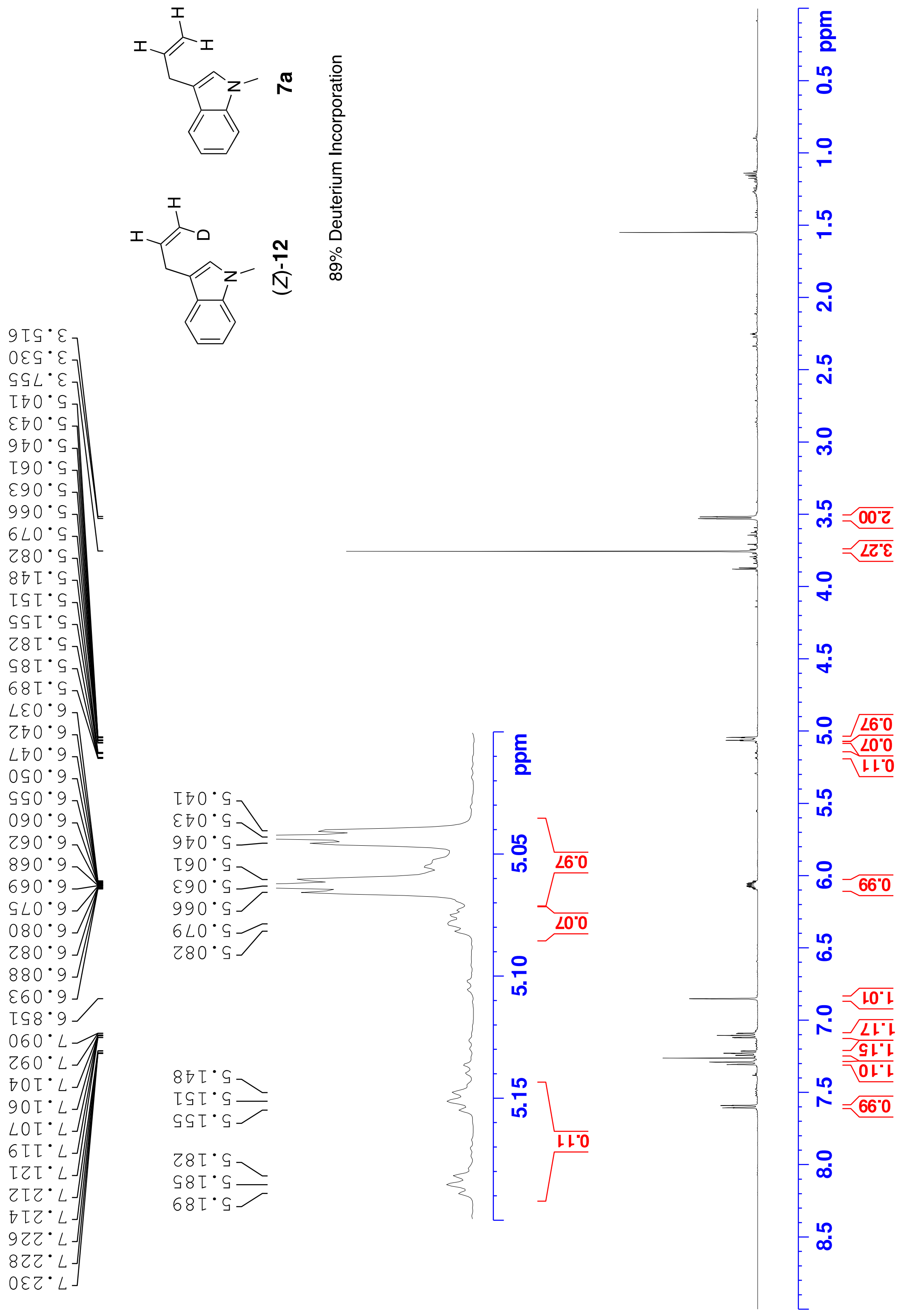
(Z)-12
7a
89% Deuterium Incorporation
ppm

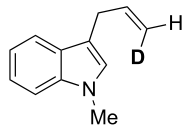
H
D
N
Me

(*Z*)-**12**
^{2}D-NMR

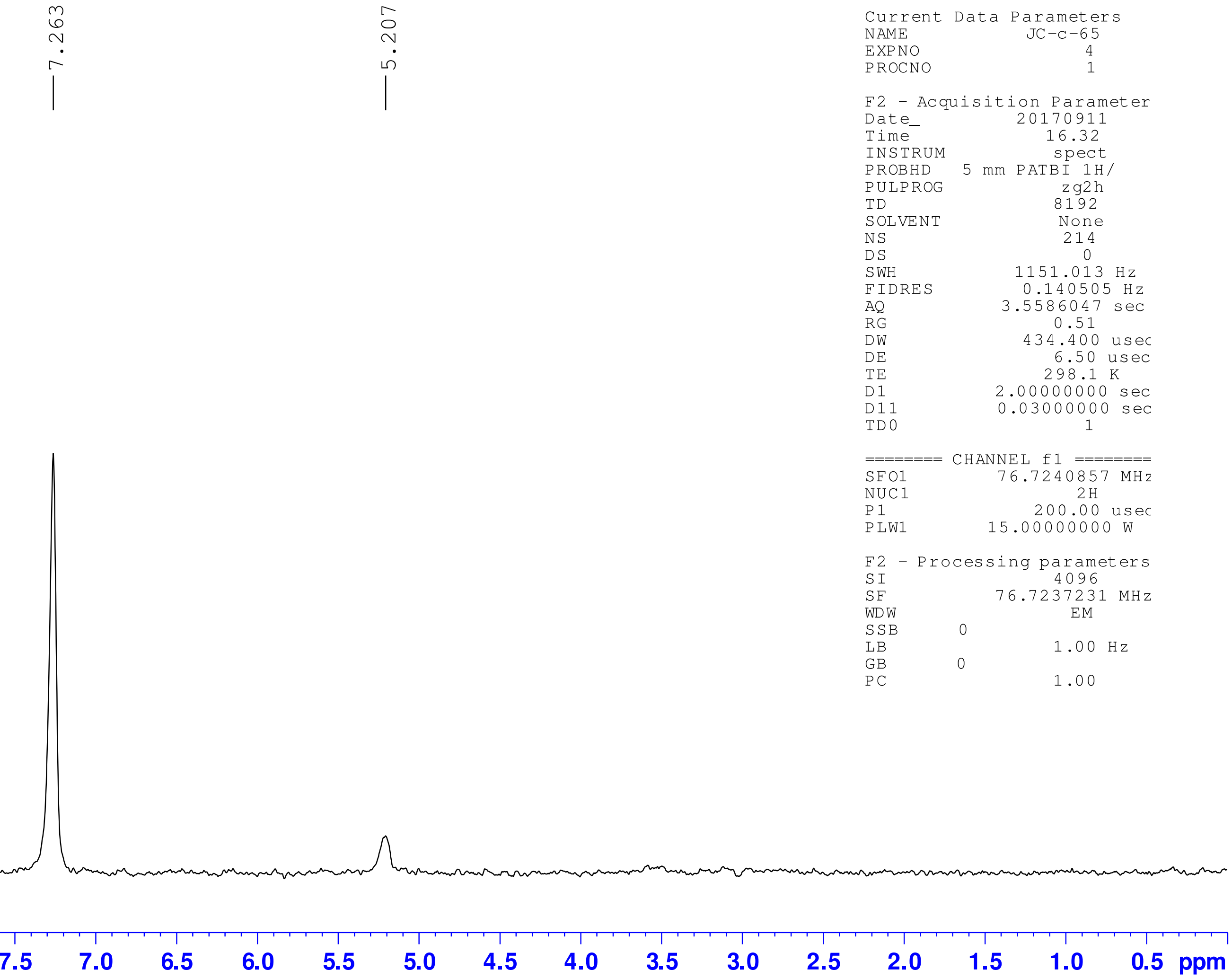
7.263
5.207
Current Data Parameters
NAME JC-c-65
EXPNO 4
PROCNO 1
F2 - Acquisition Parameter
Date_ 20170911
Time 16.32
INSTRUM spect
PROBHD 5 mm PATBI 1H/
PULPROG zg2h
TD 8192
SOLVENT None
NS 214
DS 0
SWH 1151.013 Hz
FIDRES 0.140505 Hz
AQ 3.5586047 sec
RG 0.51
DW 434.400 usec
DE 6.50 usec
TE 298.1 K
D1 2.00000000 sec
D11 0.03000000 sec
TD0 1
======== CHANNEL f1 ========
SFO1 76.7240857 MHz
NUC1 2H
P1 200.00 usec
PLW1 15.00000000 W
F2 - Processing parameters
SI 4096
SF 76.7237231 MHz
WDW EM
SSB 0
LB 1.00 Hz
GB 0
PC 1.00
8.5
8.0
7.5
7.0
6.5
6.0
5.5
5.0
4.5
4.0
3.5
3.0
2.5
2.0
1.5
1.0
0.5 ppm

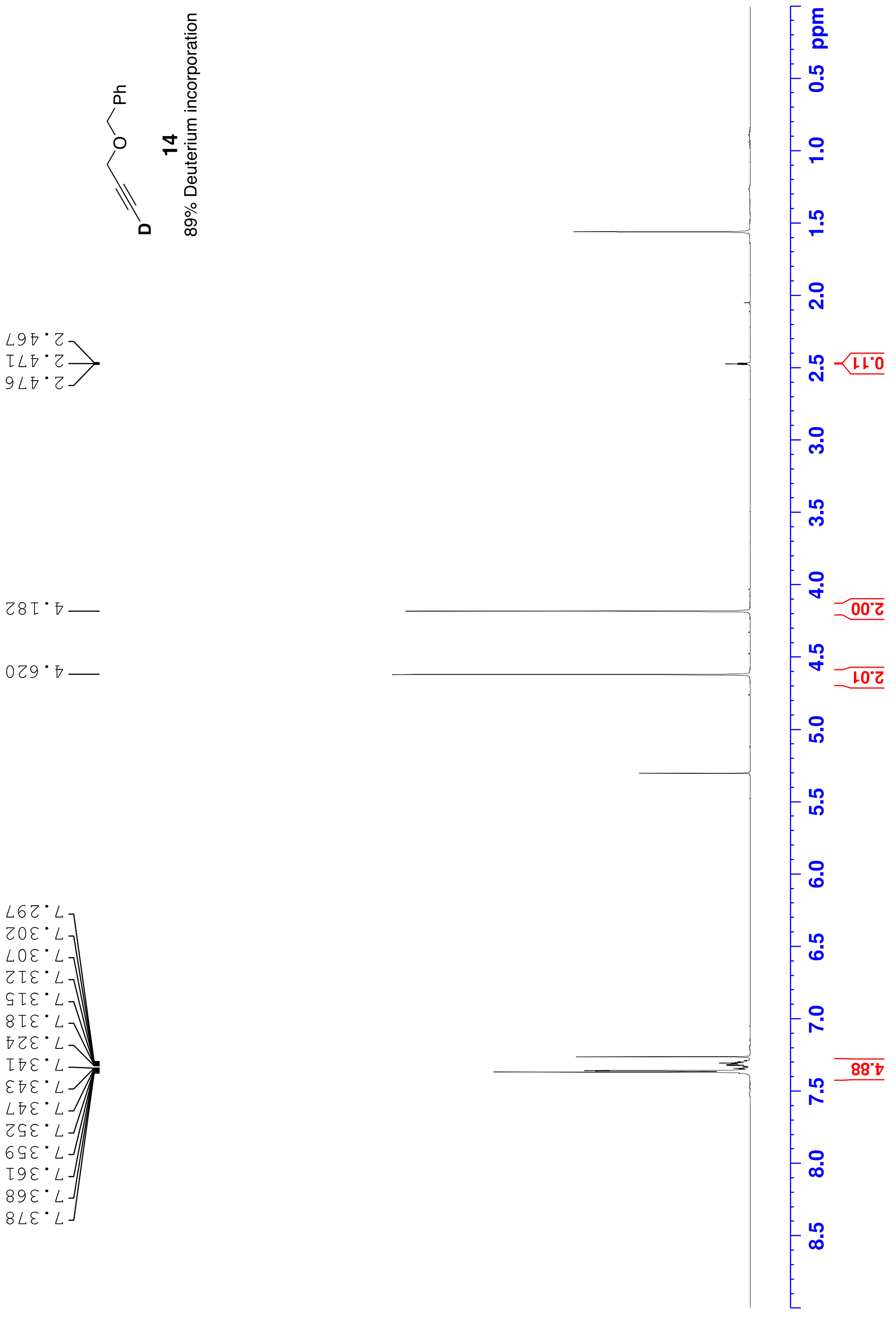
D
O
Ph
14
89% Deuterium incorporation
2.467
2.471
2.476
4.182
4.620
7.297
7.302
7.307
7.312
7.315
7.318
7.324
7.341
7.343
7.347
7.352
7.359
7.361
7.368
7.378
0.11
2.00
2.01
4.88
0.5
1.0
1.5
2.0
2.5
3.0
3.5
4.0
4.5
5.0
5.5
6.0
6.5
7.0
7.5
8.0
8.5
ppm

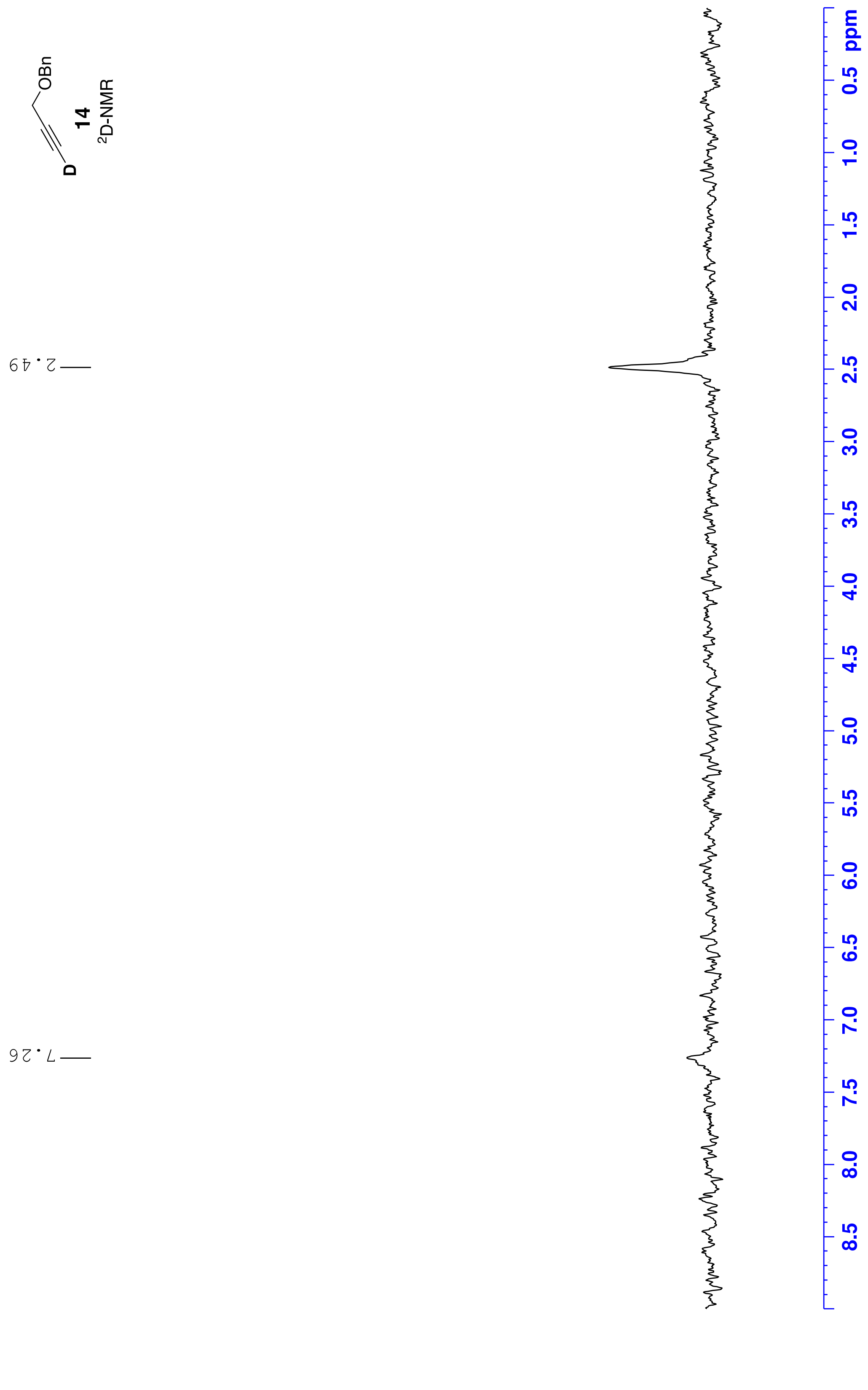
OBn
D
14
^{2}D-NMR
2.49
7.26
ppm

H-NMR of the 3h Aliquot

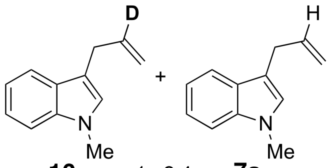

16 1 : 2.4 7a

with inseparable unreacted benzyl propargyl ether

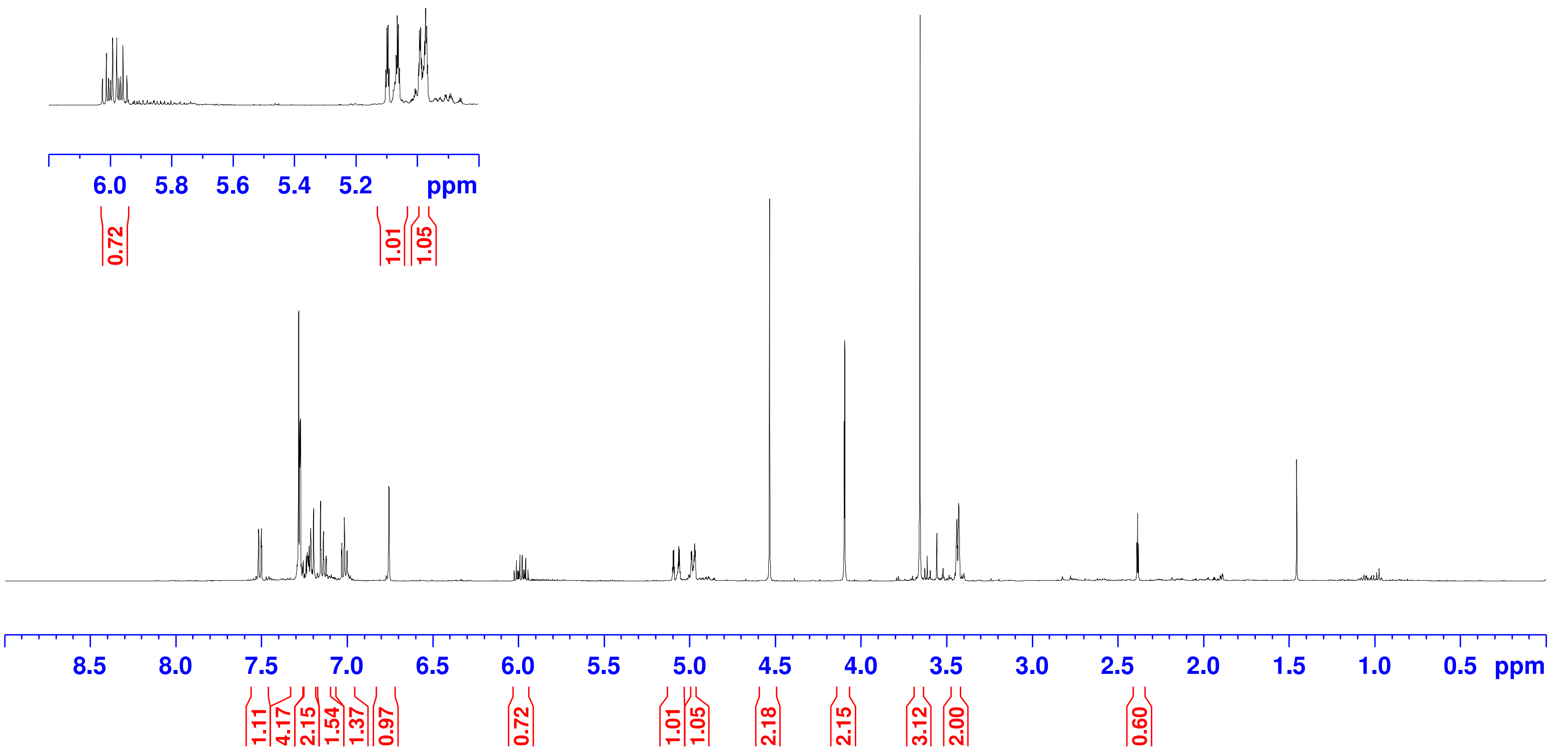

^{2}D-NMR of the purified 3h aliquot

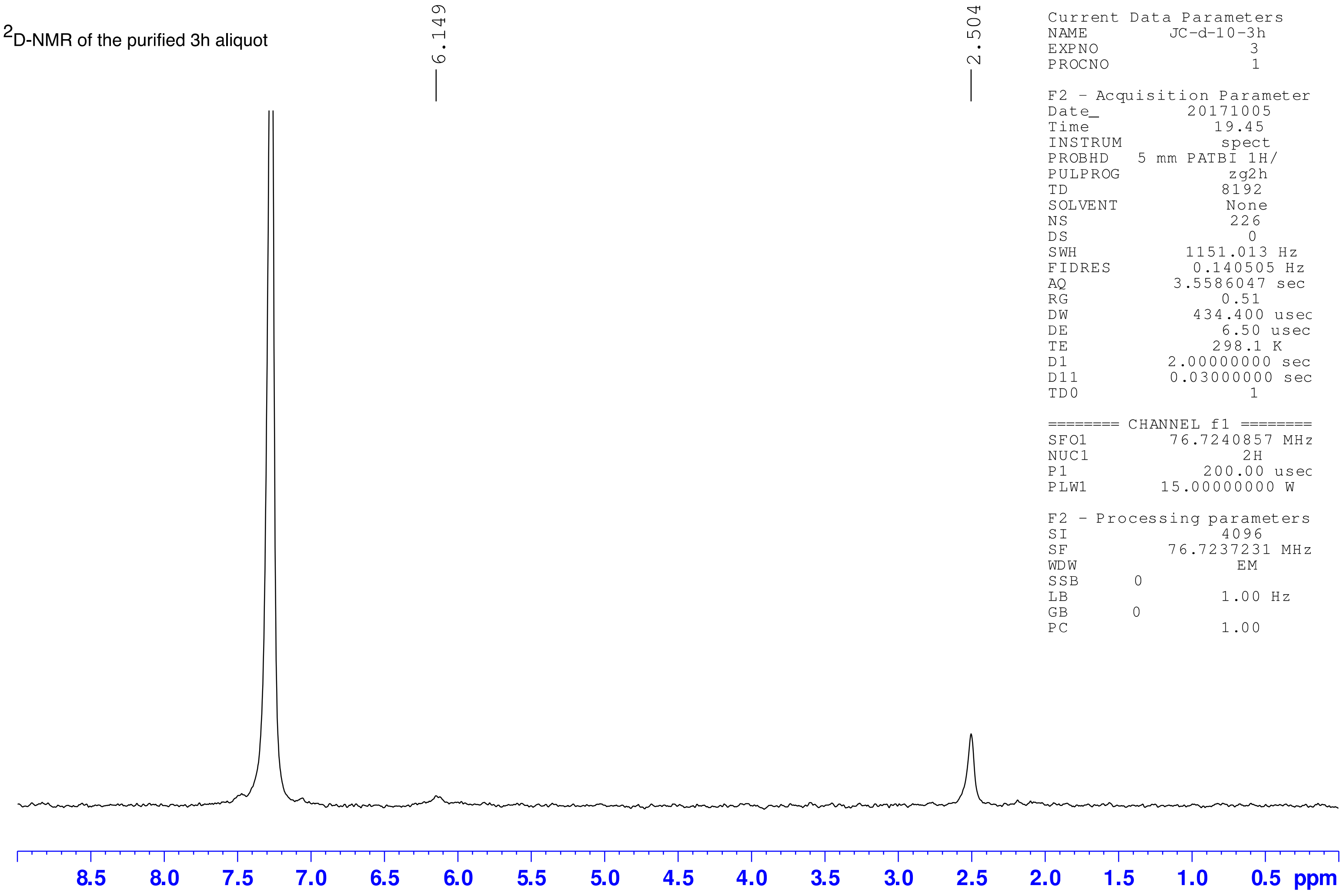

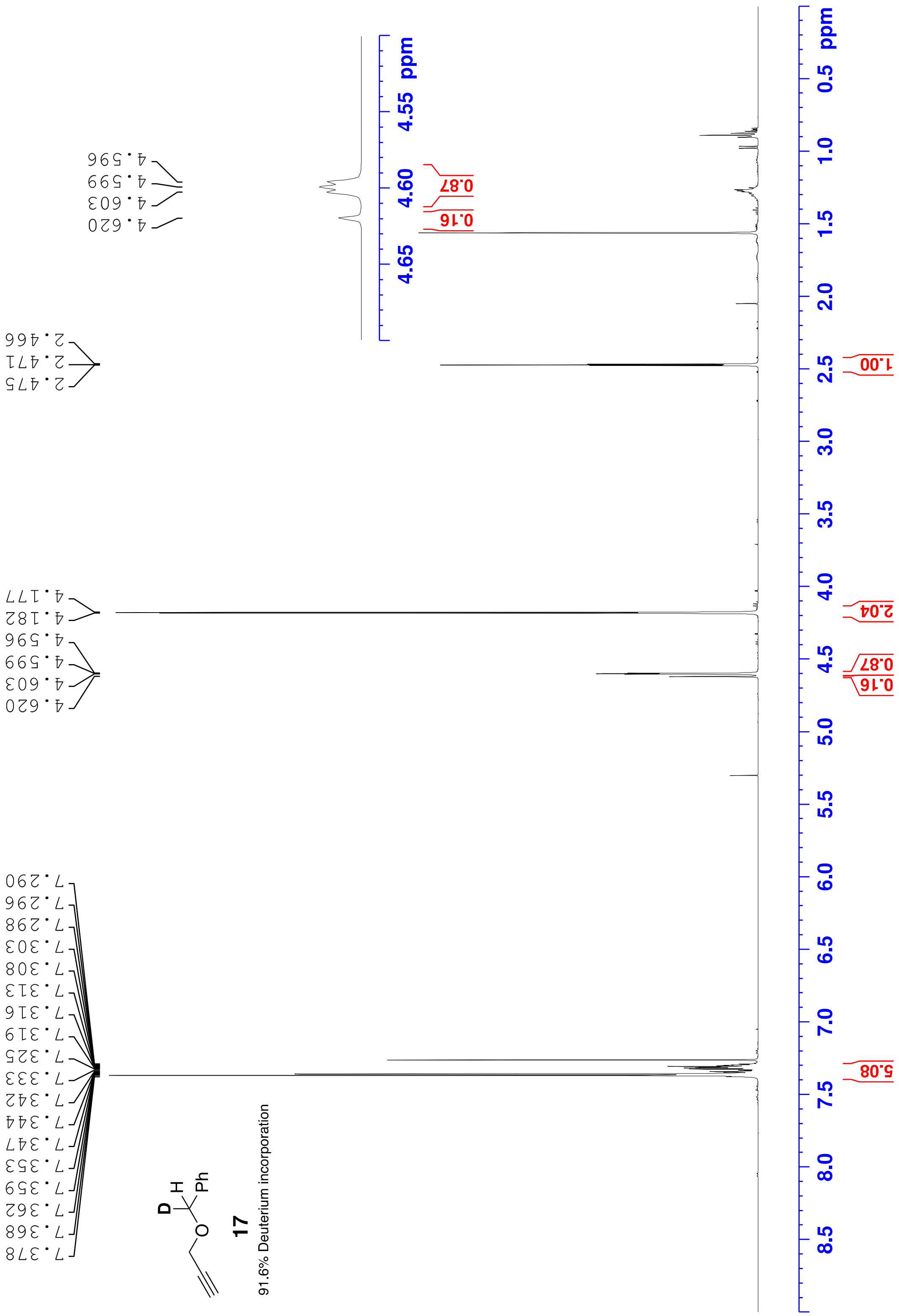
7.378
7.368
7.362
7.359
7.353
7.347
7.344
7.342
7.333
7.325
7.319
7.316
7.313
7.308
7.303
7.298
7.296
7.290
4.620
4.603
4.599
4.596
4.182
4.177
2.475
2.471
2.466
D
H
O
Ph
17
91.6% Deuterium incorporation
5.08
0.16
0.87
2.04
1.00
0.16
0.87
4.55
4.60
4.65
ppm
8.5
8.0
7.5
7.0
6.5
6.0
5.5
5.0
4.5
4.0
3.5
3.0
2.5
2.0
1.5
1.0
0.5
ppm

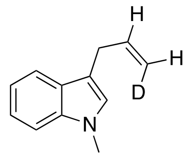

(*Z*)-**12**

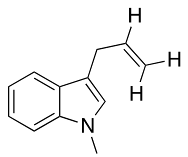

7a

Product Ratio: 1 : 2.1

Calculated KIE = 1.9

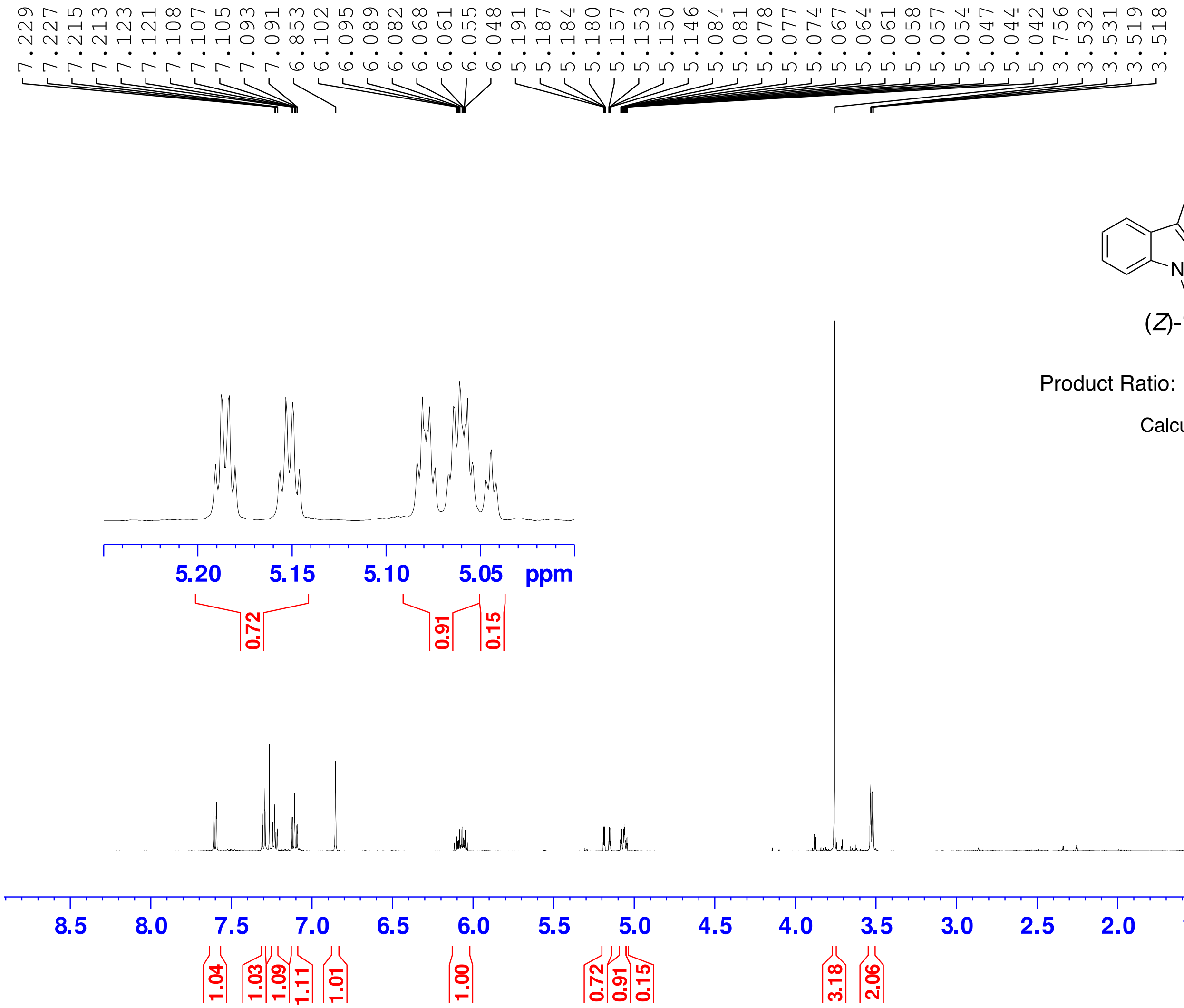

Supplement: Supplementary file 1 [file SC-009-C7SC05477G-s001.pdf]
